# A Systematic Review and Meta-Analysis of Multiple Airborne Pollutants and Autism Spectrum Disorder

**DOI:** 10.1371/journal.pone.0161851

**Published:** 2016-09-21

**Authors:** Juleen Lam, Patrice Sutton, Amy Kalkbrenner, Gayle Windham, Alycia Halladay, Erica Koustas, Cindy Lawler, Lisette Davidson, Natalyn Daniels, Craig Newschaffer, Tracey Woodruff

**Affiliations:** 1 Department of Obstetrics, Gynecology & Reproductive Sciences, Program on Reproductive Health and the Environment, University of California, San Francisco, CA, United States of America; 2 Department of Obstetrics, Gynecology & Reproductive Sciences, Program on Reproductive Health and the Environment, University of California, San Francisco, CA, United States of America; 3 Joseph J. Zilber School of Public Health, University of Wisconsin-Milwaukee, Milkwaukee, WI, United States of America; 4 Division of Environmental and Occupational Disease Control, California Department of Public Health, Richmond, CA, United States of America; 5 Autism Science Foundation, New York, NY, United States of America; 6 Department of Pharmacology and Toxicology, Rutgers University, New Brunswick, NJ, United States of America; 7 Scientific consultant to the University of California, San Francisco, CA, United States of America; 8 Division of Extramural Research and Training, National Institute of Environmental Health Sciences, Research Triangle Park, NC, United States of America; 9 Department of Obstetrics and Gynecology, Kaiser Permanente, Oakland, CA, United States of America; 10 Department of Obstetrics, Gynecology & Reproductive Sciences, Program on Reproductive Health and the Environment, University of California, San Francisco, CA, United States of America; 11 Department of Epidemiology and Biostatistics, Drexel University, Philadelphia, PA, United States of America; 12 Department of Obstetrics, Gynecology & Reproductive Sciences, Program on Reproductive Health and the Environment, University of California, San Francisco, CA, United States of America; CSIR-Indian Institute of Toxicology Research, INDIA

## Abstract

**Background:**

Exposure to ambient air pollution is widespread and may be detrimental to human brain development and a potential risk factor for Autism Spectrum Disorder (ASD). We conducted a systematic review of the human evidence on the relationship between ASD and exposure to all airborne pollutants, including particulate matter air pollutants and others (e.g. pesticides and metals).

**Objective:**

To answer the question: “is developmental exposure to air pollution associated with ASD?”

**Methods:**

We conducted a comprehensive search of the literature, identified relevant studies using inclusion/exclusion criteria pre-specified in our protocol (registered in PROSPERO, CRD # 42015017890), evaluated the potential risk of bias for each included study and identified an appropriate subset of studies to combine in a meta-analysis. We then rated the overall quality and strength of the evidence collectively across all air pollutants.

**Results:**

Of 1,158 total references identified, 23 human studies met our inclusion criteria (17 case-control, 4 ecological, 2 cohort). Risk of bias was generally low across studies for most domains; study limitations were related to potential confounding and accuracy of exposure assessment methods. We rated the quality of the body of evidence across all air pollutants as “moderate.” From our meta-analysis, we found statistically significant summary odds ratios (ORs) of 1.07 (95% CI: 1.06, 1.08) per 10-μg/m^3^ increase in PM_10_ exposure (n = 6 studies) and 2.32 (95% CI: 2.15, 2.51) per 10-μg/m^3^ increase in PM_2.5_ exposure (n = 3 studies). For pollutants not included in a meta-analysis, we collectively evaluated evidence from each study in rating the strength and quality of overall evidence considering factors such as inconsistency, imprecision, and evidence of dose-response. All included studies generally showed increased risk of ASD with increasing exposure to air pollution, although not consistently across all chemical components.

**Conclusion:**

After considering strengths and limitations of the body of research, we concluded that there is “limited evidence of toxicity” for the association between early life exposure to air pollution as a whole and diagnosis of ASD. The strongest evidence was between prenatal exposure to particulate matter and ASD. However, the small number of studies in the meta-analysis and unexplained statistical heterogeneity across the individual study estimates means that the effect could be larger or smaller (including not significant) than these studies estimate. Our research supports the need for health protective public policy to reduce exposures to harmful airborne contaminants among pregnant women and children and suggests opportunities for optimizing future research.

## Introduction

Air pollution is a serious public health issue, responsible for over seven million deaths a year worldwide [1]. In addition to mortality, cardiovascular and respiratory diseases have been identified as primary health concerns related to exposure [2–4]. More recently, the central nervous system was proposed as another organ negatively affected by air pollutants [5], and prenatal air pollution has been identified as having potentially greater impacts than adult exposures [6].

Air pollution is composed of a large number of compounds coming from a wide variety of sources, notably vehicle and powerplant emissions. Compounds include well-characterized pollutants such as particulate matter (PM) and ozone, and lesser-characterized airborne chemicals like metals and pesticides. Studies of individual components of air pollution have found links to neurodevelopmental outcomes, including effects on intelligence quotient (IQ), language development, executive function, and psychomotor development [7–10]. Studies in animals have also found that developmental exposure to air pollution is related to functional and structural brain effects [11–15].

In particular, a number of studies have reported a relationship to Autism Spectrum Disorder (ASD). ASD is a group of complex neurodevelopmental disabilities defined by a spectrum of behaviors, characterized generally by difficulties with social interaction and communication accompanied by restricted or repetitive behaviors or interests. ASD has well-characterized comorbidities and increasing prevalence, estimated within the United States (US) at 1 in 68 children in 2010 (1.5%) [16, 17]. ASD has few and limited effective treatments and is associated with considerable financial and medical burden; research into its etiology has increased dramatically over the last decade to address the increasing prevalence. Many genetic, lifestyle, and environmental factors are being explored—current thinking suggests that multiple causes are likely to blame, including a number of genetic pathways and environmental chemical exposures, or their interaction [18–20]. The widespread availability of air pollution exposure data generated from decades of interest in air quality’s other health effects has led to air pollution as one of the more-studied candidate ASD environmental risk factors.

However, a systematic review and meta-analysis of the evidence for the relationship between air pollution and ASD had been lacking. While systematic review methods have been used for decades in the clinical sciences [21, 22], such methods have only recently been developed and utilized in environmental health sciences [23–30]. Therefore, we applied the Navigation Guide review methodology [23, 26] to answer the question “does developmental exposure to air pollution affect diagnosis of ASD?”

## Methods

### The Navigation Guide Systematic Review Methodology

To conduct our review of ASD and air pollution we applied the Navigation Guide, a systematic and transparent methodology for synthesizing the available scientific evidence [23, 26]. The Navigation Guide systematic review methodology is based on Cochrane/GRADE methods [21, 22] and includes all the same elements (protocol, development, risk of bias evaluation, evidence evaluation, etc.) but accounts for the differences in evidence and decision context inherent to environmental health assessments, i.e., the reliance on human observational studies in the absence of randomized controlled trials (RCTs), and the fact that population exposure to exogenous chemicals precedes evidence of their safety. To date, the Navigation Guide method has been used in 3 case studies [25, 27, 28].

We assembled a diverse team of reviewers in August 2014 with expertise in epidemiology, air pollution/exposure assessment, ASD outcome assessment, biostatistics, library sciences, and/or systematic review methodology. We developed a protocol to outline the process for conducting the systematic review prior to initiating the study and registered the protocol with an international database for systematic reviews in March 2015. Each member of the review team also filled out a conflict of interest statement at the initiation of their involvement with the protocol to document any potential financial or other conflicts of interest, and these were appended to the protocol. Each of the protocol steps are described below and the protocol is available on PROSPERO (http://www.crd.york.ac.uk/PROSPERO/; CRD # 42015017890).

#### Specify the study question

Our objective was to answer the question: “Does developmental exposure to air pollution affect diagnosis of Autism Spectrum Disorder (ASD)?”

We developed a “Participants”, “Exposure,” “Comparator” and “Outcomes” (PECO) statement, as follows:

**P**articipants: Humans

**E**xposure: Any developmental exposure to air pollution that occurred prior to the ASD assessment.

- *“Any developmental exposure” is defined as maternal or paternal exposure incurred any time “in proximity to” conception (as defined by authors of the included study)*, *or exposures to offspring incurred in utero or in the perinatal or childhood period*.
- *“Air pollution” is defined as any indoor or outdoor source of any inhaled airborne environmental chemical*, *EXCLUDING active and passive smoking*.
- *Exposures “prior to the ASD assessment” include direct and proxy measures for this time period*.

**C**omparator: Humans exposed to lower levels of air pollution than the more highly exposed humans.

- *This definition is intended to include groups defined by ASD case-control studies; for instance comparing the air pollution* *exposure levels for people with ASD versus those without*.

**O**utcome: Any clinical diagnosis or other continuous or dichotomous scale assessment of ASD.

- *Clinical ASD diagnosis can be based on the International Classification of Diseases (ICD) 9*, *ICD 10*, *Diagnostic and Statistical Manual of Mental Disorders (DSM) 5*, *or DSM-IV criteria*, *including difficulties in social interaction*, *verbal and nonverbal communication and repetitive behaviors*.

#### Select the Evidence

*Search methods*. Our search was not limited by language or start-date of publication. We searched several online databases (PubMed, ISI Web of Science, Biosis Previews, Embase, Google Scholar, and Toxline) between November 3–5, 2014 using the search terms in S1 Table. We used the Medical Subject Headings (MeSH) database to compile synonyms for air pollution and ASD-related outcomes (http://www.ncbi.nlm.nih.gov/mesh/68000397, http://www.ncbi.nlm.nih.gov/mesh/68001321). We separated the exposure-related search into two general categories: one based on the route of exposure (air inhalation, along with appropriate synonyms in a Boolean search using the “OR” statement) and the other based on typical chemical composition of air pollution (ozone, particulate matter, etc. in a Boolean search using the “OR” statement). We intentionally crafted a broad search strategy for exposure to capture all studies evaluating any indoor/outdoor chemical air pollutant for inclusion, excluding those related to cigarette smoke. These two categories of search terms were then combined in a Boolean search using the “OR” statement to create the collection of exposure search terms. For the outcome, we combined “autism spectrum disorder” and its synonyms in a Boolean search using the “OR” statement (S1 Table).

We then combined the exposure and outcome terms using a Boolean search using the “AND” statement. We searched for terms in titles and abstracts (using the [tiab] function in PubMed, topic search in Web of Science and Biosis Previews; “ti,ab.” function in Embase) or in MeSH headings (using the [mh] function in PubMed). We searched additional toxicological websites and grey literature databases (S2 Table) intended to capture papers and reports from the non-peer reviewed literature (November 6–13, 2014). We performed “snowball searching,” which included hand-searching the reference lists of all included studies as well as review articles identified in the screening process, and using Web of Science to search for articles that cited the included studies. We also reached out to a group of recognized experts in this field from the Environmental Epidemiology of Autism Research Network (EEARN) in March 2015 to request review of the included studies to identify whether we had missed any relevant studies.

*Study selection criteria*. We included studies only if: 1) the report contained original data from human studies; 2) there was a measure or report of air pollution exposure prior to the diagnosis or assessment of ASD; and 3) there was a comparator (control group or exposure range comparison). References that did not meet all of these criteria were excluded from consideration (S3 File).

We screened references in duplicate for inclusion using structured forms in DRAGON (ICF International; available at: http://www.icfi.com/insights/products-and-tools/dragon-online-tool-systematic-review) and DistillerSR (Evidence Partners; available at: http://www.systematic-review.net). To determine eligibility, each reference from the literature search had the title and abstract independently reviewed by two of the five reviewers (ND, AH, AK, LD, GW) in a non-random assignment to ensure that the same two authors did not always screen the same references. In the event that an abstract was missing or there were discrepancies between the two reviewers, the default was to move the reference forward for full text review. Two of the five reviewers (ND, AH, AK, LD, GW) then independently performed a full-text review to evaluate inclusion criteria of each reference not excluded by title/abstract screening. An additional reviewer (PS) screened five percent of the titles/abstracts and full-texts for quality assurance.

*Data collection and management*. Four authors (ND, LD, PS, EK) and a UCSF research assistant (HT) independently extracted data relating to study characteristics and outcome measures (S3 Table) from all included articles into a DRAGON database. We contacted the corresponding author when information pertinent to our study question was missing or. A third author (JL) performed QA/QC on all of the studies by reviewing the studies and its extracted data to check for accuracy.

#### Reviewing the evidence

*Assessing the risk of bias for each included study*. We assessed risk of bias for each included study using a modified instrument we developed based on the Cochrane Collaboration’s “Risk of Bias” tool and the Agency for Healthcare Research and Quality’s (AHRQ) domains, i.e., selection bias, confounding, performance bias, attrition bias, detection bias, and reporting bias [21, 31]. We modified the wording and instructions for several of these domains beforehand to make it specific for our study question and the types of evidence or study characteristics that we anticipated.

In particular, because of the complexity of methods for assessing exposure to air pollution, we ultimately developed a novel risk of bias instructions for exposure assessment that specified a list of important considerations, i.e., modeling, monitoring, biomarkers, etc. Review authors developed this tool collectively and in collaboration with a known expert in the air pollution field (HC) as well as the EEARN working group. Review authors were instructed to separately rate the exposure assessment risk of bias for each air pollutant chemical or classes of chemicals reported in the study under review. The justification for this was based on empirical evidence demonstrating that similar exposure assessment methods for different air pollutant compounds are heterogeneous in terms of their internal validity [32, 33]. We also elicited expert opinions from the EEARN working group to develop a list of potentially important confounders or effect modifiers to include in the analysis. These are outlined in the protocol in the risk of bias tool, under the confounding domain, along with justification for inclusion and relevant citations.

We assigned each risk of bias domain as “low,” “probably low,” “probably high,” or “high” risk of bias, or “not applicable” (risk of bias area not applicable to study) according to specific criteria as described in our risk of bias instruments (S2 File). Two of the eight review authors with subject-matter expertise (GW, AH, CN, AK, CL, TW, PS, EK) independently recorded risk of bias determinations for each included study. In the event that one of the review authors was a coauthor of the study in question, they recused themselves from rating the risk of bias for that particular study. We held an in-person meeting to review rationales and ratings for each study and come to consensus. Based on this discussion, two review authors (JL, EK) subsequently reviewed ratings for all included studies to ensure consistency across studies with similar study populations or study design.

*Statistical analyses*. We assessed study characteristics from included articles to evaluate comparability of findings based on pre-determined features as outlined in our protocol (i.e., study features, study population, exposure assessment method, and outcome assessment method) to determine which study results were potentially suitable for meta-analysis. In particular, review authors had decided beforehand that different air pollutant chemicals or classes of compounds (i.e., heavy metals, pesticides, and criteria air pollutants) should be analyzed separately in the absence of empirical evidence to suggest that we could combine these effect estimates. From the assessment of specified characteristics, we determined that two subsets of studies in which exposure was measured during pregnancy or prior to assessment of ASD met these criteria. The first was for particulate matter less than 10 μm (PM_10_) (comprised of six studies), and the second was for fine particulate matter, less than 2.5 μm (PM_2.5_) (comprised of three studies), from studies where exposure was measured during pregnancy prior to diagnosis or assessment of ASD.

PM_10_ or PM_2.5_ concentrations and their standard errors (reported in the study or calculated from reported 95% confidence interval and sample sizes) were extracted from each study for the meta-analysis. We extracted adjusted odds ratio (OR) estimates reported for a continuous increment increase in exposure, then standardized effect estimates across all studies by computing adjusted OR estimates per 10 μg/m^3^ increase in PM. In the event that such an estimate was unavailable or could not be calculated from the data available in the published article, we contacted study authors to request these data be made available to us. We then combined the standardized effect estimates from each study in a random effects model with inverse variance weighting. The result was an estimate of the combined summary OR per 10 μg/m^3^ increase in PM, accounting for within- and between-study variability. We used R statistical software (version 3.0.1) and the “metafor” package for analyses.

We evaluated statistical heterogeneity across study estimates in the meta-analysis using Cochran’s Q statistic with p≤0.05 as our cut off for statistical significance and I^2^ [21], as previously described [24, 27, 30]. If statistical heterogeneity was present, we used leave-one-out analysis to identify the study or studies contributing, evaluated potential study characteristics (for example, study location, study population, study design, adjusted confounders, timing of exposure, etc.) to explain the source, and incorporated hierarchical cluster structures in the data analysis to statistically account for the heterogeneity.

Although data for other contaminants were not amenable to a meta-analysis due to insufficient number of studies and/or the existence of heterogeneity across study characteristics, we created scatterplots of effect estimates by contaminant to visually inspect these results and evaluate associations.

*Rating the* ***quality*** *of evidence across all included studies*. We rated the quality of the overall body of evidence as either”high,”“moderate” or”low.” The Navigation Guide approach follows that established by the Grading of Recommendations Assessment Development and Evaluation (GRADE) method used in the clinical field; i.e., by first assuming an initial quality rating to the body of evidence and then considering adjustments (“downgrades” or “upgrades”) based on the characteristics of the included studies to reach a final quality rating [34].

We assumed an initial rating of “moderate” quality to the human bodies of evidence (observational studies), based on previously described rationale [26] and consistent with previous case studies [27, 28, 30], based on consideration of both the values and limitations of observational data in assessing associations between exposure and health outcomes in environmental health. We considered downgrades and upgrades to this initial quality rating based on 8 specific factors and instructions for consideration (S4 Table): risk of bias, indirectness of evidence, inconsistency of evidence, imprecision of evidence, potential for publication bias, large magnitude of effect, dose response relationship, and whether residual confounding would minimize the overall effect estimate. Possible ratings were 0 (no change from initial quality rating), -1 (1 level downgrade) or– 2 (2 level downgrade); +1 (1 level upgrade) or + 2 (2 level upgrade). Review authors independently evaluated the quality of the evidence and then compared their ratings and rationale for each quality category. We discussed our ratings as a group and recorded our rationale. Consistent with GRADE, we did not automatically add together the ratings for each downgrade and upgrade factor to create a score, e.g., a (-1) downgrade for each of 2 factors does not necessarily translate into a (-2) downgrade overall. We used our judgment to decide the weight of each downgrade or upgrade in the final overall quality rating.

*Rating the* ***strength*** *of the evidence across all included studies*. We rated the overall strength of the body of evidence based on 4 considerations: (1) Quality of body of evidence (i.e., the rating from the previous step); (2) Direction of effect; (3) Confidence in effect (likelihood that a new study would change our conclusion); and (4) Other compelling attributes of the data that may influence certainty. We used these considerations to assign the overall strength rating, according to the definitions specified in the Navigation Guide for “sufficient evidence of toxicity,” “limited evidence of toxicity,” “inadequate evidence of toxicity,” or “evidence of lack of toxicity” (Table 1), which are based on categories used by the International Agency for Research on Cancer (IARC) [35], with the definitions of each category based on IARC, the U.S. Preventive Services Task Force, and U.S. EPA for evidence integration [35–38]. Review authors independently evaluated the strength of the evidence and compared their evaluations, resolved discrepancies by discussion, and recorded the collective rationale for decisions.

**Table 1:** Strength of evidence definitions.

Strength Rating	Definition
Sufficient evidence of toxicity	A positive relationship is observed between exposure and outcome where chance, bias, and confounding can be ruled out with reasonable confidence.^a^ The available evidence includes results from one or more well-designed, well-conducted studies, and the conclusion is unlikely to be strongly affected by the results of future studies.^b^
Limited Evidence of Toxicity	A positive relationship is observed between exposure and outcome where chance, bias, and confounding cannot be ruled out with reasonable confidence. Confidence in the relationship is constrained by such factors as: the number, size, or quality of individual studies, or inconsistency of findings across individual studies^a^. As more information becomes available, the observed effect could change, and this change may be large enough to alter the conclusion.
Inadequate Evidence of Toxicity	The available evidence is insufficient to assess effects of the exposure. Evidence is insufficient because of: the limited number or size of studies, low quality of individual studies, or inconsistency of findings across individual studies.^a^ More information may allow an assessment of effects.
Evidence of Lack of Toxicity	No relationship is observed between exposure and outcome, and chance, bias and confounding can be ruled out with reasonable confidence. The available evidence includes consistent results from more than one well-designed, well-conducted study at the full range of exposure levels that humans are known to encounter, and the conclusion is unlikely to be strongly affected by the results of future studies.^a^ The conclusion is limited to the age at exposure and/or other conditions and levels of exposure studied.

^a^ Language for the definitions of the rating categories were adapted from descriptions of levels of certainty provided by the U.S. Preventive Services Task Force Levels of Certainty Regarding Net Benefit.[35]

^b^The Navigation Guide rates the quality and strength of evidence of human and non-human evidence streams separately as “sufficient”, “limited”, “inadequate” or “evidence of lack of toxicity” and then these two ratings are combined to produce one of five possible statements about the overall strength of the evidence of a chemical’s reproductive/developmental toxicity. The methodology is adapted from the criteria used by the International Agency for Research on Cancer (IARC) to categorize the carcinogenicity of substances [35] except as noted.

## Results

### Included studies

**Fig 1:**
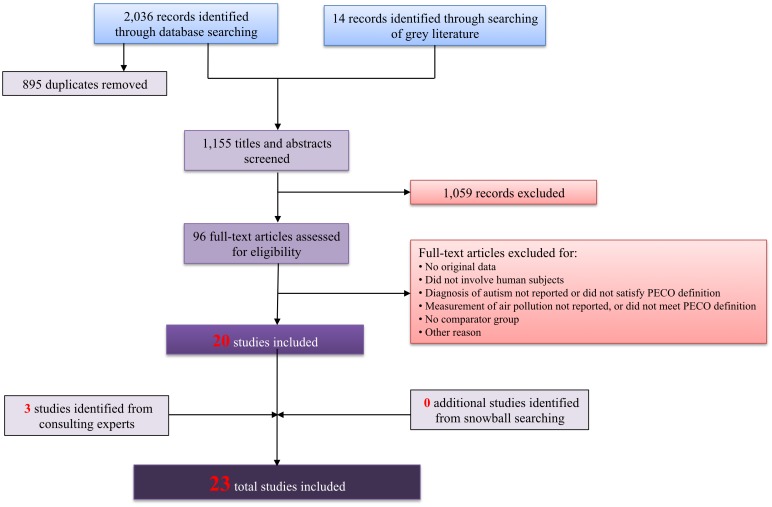
Search results for studies relevant to air pollution exposure and ASD outcome.

We retrieved a total of 1,155 references from our literature search, of which 96 met inclusion criteria based on title and abstract screening and 20 further met inclusion criteria based on full text screening [39–58]. We also identified three additional studies [59–61] by consulting with experts in the field (those affiliated with the EEARN listserve) who reviewed our list of included studies to determine whether there were additional studies that had not yet been identified, bringing the total number of screened references to 1,158 and the total number of included studies to 23 (Fig 1). Some of these studies initially had multiple separate records—for instance, a conference abstract plus a subsequently published manuscript; the information from these records was ultimately collectively assessed using information from the peer-reviewed manuscript supplemented by further relevant details from the additional records if applicable. The 23 included studies were all published between the years 2006 to 2015, included 174 to 4,057,712 participants born between the early 1990s through 2005, were based in 4 different countries, and involved case-control, cohort, and ecological study designs (Table 2). All but one study published in Spanish [47] were in English.

**Table 2:** Characteristics of included human studies on air pollution and ASD, by publication date and study design.

Study	Study design	Study population & location	Sample size	Exposure assessment	Outcome assessment
Windham et al. 2006 [55]	Case-control	Children born in 1994 in 6 counties in the San Francisco Bay area	284 children with ASD and 657 controls	Modeled concentrations of 29 hazardous air pollutants in 1996,^a^ assigned by census tract of maternal residence at delivery (birth cert)	ASD cases ascertained from multi-source records-based surveillance of children, conducted by California CADDRE (within CDPH)
Roberts et al. 2007 [48]	Case-control	Children born between 1996–1998 in 19 counties in the Central Valley of California	465 children with ASD and 6,975 controls	Modeled concentrations of 54 pesticides applied between 1995–1998,^b^ assigned by maternal residence of maternal residence at delivery (birth cert)	ASD cases identified (by CDPH) from CA Dept of Developmental Services (DDS) files
Windham et al. 2007 [56]	Case-control	Children born between 1996–1998 in 4 counties in Southern California	3,400 children with ASD and controls frequency matched on last menstrual period in a 1:10 ratio	Modeled concentrations of 29 hazardous air pollutants in 1996,^a^ assigned by census tract of maternal residence at delivery (birth cert)	ASD cases identified (by CDPH) from CA Dept of Developmental Services (DDS) client files
Lewandowski et al. 2009 [45]	Ecological	Students in Texas school districts for academic years 2000–2001 through 2005–2006	7,022 children with ASD and 4,050,690 controls for 2001; numbers not reported for other years	Modeled concentrations of 11 toxic release pollutants in 2001 and of mercury only between 2000–2005,^c^ assigned by school district	Prevalence of ASD and other special education categories obtained from the Texas Education Agency Academic Excellence Indicator System
Kalkbrenner et al. 2010 [43]	Case-control	Children aged 8 years in North Carolina (born in 1994 and 1996) and West Virginia (born in 1992 and 1994)	383 children with ASD and 2,829 children with speech and language impairment as controls	Modeled concentrations of 35 hazardous air pollutants in 1996,^a^ assigned by census tract of birth residence	ASD cases and controls with speech and language impairment identified from records-based surveillance of children conducted by ADDM
Trousdale et al. 2010 [50]	Case-control	All children aged 8 years in the US (specifically in MD for sub-analysis) during school years 2004–2005 and 2007–2008	Not reported	Modeled concentrations of 34 hazardous air pollutants in 1996 and 89 hazardous air pollutants in 1999,^a^ assigned over entire U.S (and for MD state by county in sub-analysis)	ASD prevalence calculated using data from the U.S. Department of Education, Office of Special Education Programs and control numbers using data from the National Center for Education Statistics enrollment data (Maryland sub-analysis from Maryland State Department of Education)
Blanchard et al. 2011 [40]	Ecological	Students in Bexar County, TX (all ages) and Santa Clara County, CA (elementary school ages) in 2008	Not reported	Modeled concentrations of mercury in 2002,^a^ assigned by city block-level school districts	ASD rates obtained from the Texas Education Association and from http://www.kidsdata.org for California
Volk et al. 2011 [51]	Case-control	Children enrolled in the CHARGE study and born between 1997–2006 in California	304 children with ASD and 259 typically developing controls	Distance to freeways and major roads as proxy for traffic-related pollutant exposure; assigned by residential address during pregnancy and at birth	ASD cases identified from California DDS and children evaluated and diagnosed by study staff using the ADI-R and ADOS tools; controls were selected based on SCQ
McCanlies et al. 2012 [46]	Case-control	Children enrolled in the CHARGE study and born between 1998–2003 in California	93 children with ASD and 81 typically developing controls	Self-reported and industrial hygienist-assessed parental occupational exposures to 49 chemical agents from three months prior to conception through to either birth or weaning for breast-fed children	ASD cases recruited by California DDS and children evaluated and diagnosed using the ADI-R and ADOS tools; controls were selected based on SCQ
Becerra et al. 2013 [39]	Case-control	Children born in 1994–2006 in Los Angeles County, CA	7,603 children with ASD and 75,782 controls	Modeled concentrations of 6 pollutants between 1993–2006,^d^ assigned by residential address at delivery/birth	Autistic disorder cases identified from records of California DDS
Pino-Lopez and Romero-Asuyo 2013 [47]	Case-control	Children aged 12–36 months evaluated by the Early Intervention Service in Ciudad Real, Spain between January 2009 and February 2011	70 children with ASD and 136 unaffected controls	Self-reported parental occupation to evaluate exposure to solvents during pregnancy	ASD cases and unaffected controls identified through the Early Intervention Service of Ciudad Real
Volk et al. 2013 [52]	Case-control	Children enrolled in the CHARGE study and born between 1997–2006 in California	279 children with ASD and 245 typically developing controls	Modeled concentrations to traffic-related air pollution between 1997–2008 and monitoring data for 4 pollutants using regional air quality data between 1997–2009,^d^^,^^e^ assigned by self-reported residence history address during pregnancy and the first year of the child’s life	ASD cases identified from California DDS files, children evaluated and diagnosed by study staff using the ADI-R and ADOS tools; controls were selected based on SCQ
Windham et al. 2013 [57]	Case-control	Children born in 1994 in 6 counties in the San Francisco Bay area	284 children with ASD and 659 controls	Self-reported parental occupation on birth certificate, coded by occupational medicine-certified physician to categorize broad chemical exposures	ASD cases ascertained from multi-source records-based surveillance of children conducted by California CADDRE
Jung et al. 2013 [42]	Cohort	Children aged less than 3 years in 2000 enrolled in prospective cohort study in Taiwan	342 children with ASD and 48,731 non-ASD controls	Modeled concentrations of pollutants between 1996–2009,^d^ assigned by post-code levels in the 1–4 years preceding ASD diagnosis	ASD and non-ASD children in cohort identified based on diagnosis codes provided in the Taiwan National Insurance Research Database
Roberts et al. 2013 [49]	Cohort	Children of Nurses’ Health Study II participants born between 1987–2002 in the US	325 children with ASD and 22,098 controls	Modeled concentrations of 14 ambient hazardous air pollutants between 1990–2002,^a^ assigned by census tract by mother’s address approximately around the year of birth	ASD cases identified based on Nurses’ Health Study II participant’s response to questionnaire, validated by administration of the ADI-R to a small, random subset of case mothers
Gong et al. 2014 [41]	Case-control	Twins born after July 1, 1992 and enrolled in the CATSS longitudinal study in Stockholm, Sweden	109 children with ASD and 3,051 healthy controls	Modeled historical emissions to estimate exposures for two pollutants (PM_10_ and NO_x_) between 1992–2009, assigned by residential address during pregnancy, child’s first year of life, and the year before ASD diagnosis	ASD cases and controls identified after assessment using A-TAC tool at 9 and 12 years of age conducted by the CATSS
Shelton et al. 2014 [58]	Case-control	Children enrolled in the CHARGE study and born after 2003 in California	486 children with ASD and 315 typically developing children as controls	Modeled concentrations of 4 classes of pesticides between 1997–2008,^b^ assigned by prenatal and birth residential address	ASD cases identified from California DDS files, children evaluated and diagnosed using the ADI-R and ADOS tools by study staff; controls were selected based on SCQ
Volk et al. 2014 [53]	Case-control	Children enrolled in the CHARGE study in California	251 children with ASD and 156 controls	Modeled concentrations to traffic-related air pollution and monitoring data for 4 pollutants using regional air quality data between 1997–2009,^e^^,^^f^ assigned by prenatal and birth residential address	ASD cases identified from California DDS files, children evaluated and diagnosed by study staff using the ADI-R and ADOS tools; controls were selected based on SCQ
von Ehrenstein et al. 2014 [54]	Case-control	Children born between 1995–2006 in Los Angeles County	768 children with ASD and 147,954 controls	Monitoring data for 24 hazardous air pollutants within a 5-km radius of birth address	Cases identified from California DDS files of children served for autistic disorder
Raz et al. 2014 [61]	Case-control	Children of Nurses’ Health Study II participants born between 1990–2002 in the US	245 children with ASD and 1,522 controls	Modeled concentration from monitoring data for two pollutants (PM_10_ and PM_10-2.5_),^f^ Assigned by prenatal, pregnancy and birth residential address	ASD cases identified based on Nurses’ Health Study II participant’s response to questionnaire, and validated by administration of the ADI-R to a random subset of case mothers
Kalkbrenner et al. 2015 [44]	Case-control	Children born in North Carolina in 1994 (8 counties), 1996 (8 counties), 1998 (9 counties), and 2000 (10 counties) and born in 6 San Francisco Bay area counties in 1996	645 children with ASD and 12,434 controls for North Carolina and 334 children with ASD and 2,232 controls for California	Modeled concentration of one pollutant from monitoring data,^f^ assigned by birth certificate address	ASD cases identified from multi-source records-based surveillance of children conducted by the ADDM in North Carolina and California CADDRE
Dickerson et al. 2015 [59]	Ecological	Children 8 years of age in 2000, 2002, 2004, 2006 and 2008 from Arizona, Maryland, New Jersey, South Carolina, and Utah	4,486 children with ASD from 2489 census tracts	Modeled concentrations of 3 toxic release pollutants between 1991–1999,^c^ assigned by census tract using residence at the time of surveillance (8 years of age)	ASD cases identified from records-based surveillance of children conducted by ADDM network
Dickerson et al. 2016 [60]	Ecological	Children 8 years of age in 2000, 2002, 2004, 2006 and 2008 from Arizona, Maryland, New Jersey, South Carolina, and Utah	4,486 children with ASD from 2489 census tracts	Modeled concentrations of 3 hazardous air pollutants in 1999,^a^ assigned by census tract at the time of surveillance (8 years of age)	ASD cases identified from records-based surveillance of children conducted by ADDM network

^a^ Data from US EPA National-scale Air Toxics Assessment (NATA)^;^

^b^ Data from California Department of Pesticide Regulation (DPR)^;^

^c^ Data from US Toxic Release Inventory (TRI)^;^

^d^ Data from nearest air monitoring stations^;^

^e^ Data from CALINE4 dispersion model^;^

^f^ Data from US EPA Air Quality System (AQS)

Abbreviations: ADDM, Autism and Developmental Disabilities Monitoring; ADI-R, Autism Diagnostic Interview, Revised; ADOS, Autism Diagnostic Observation Schedule; AQS, Air Quality System; ASD, autism spectrum disorder; A-TAC, Autism Tics, ADHD, and other Comorbidities inventory; CADDRE, Centers for Autism and Developmental Disabilities Research and Epidemiology; CALINE4, California Line Source Dispersion Model, version 4; CATSS, Children from the Child and Adolescent Twin Study in Sweden; CHARGE, Childhood Autism Risks from Genetics and the Environment; CDPH, CA Department of Public Health; DDS, Department of Developmental Services; NIOSH, National Institute for Occupational Safety and Health; SCQ, Social Communication Questionnaire; TRI, Toxics Release Inventory; USC, University of Southern California

### Risk of bias assessment for individual studies

**Fig 2:**
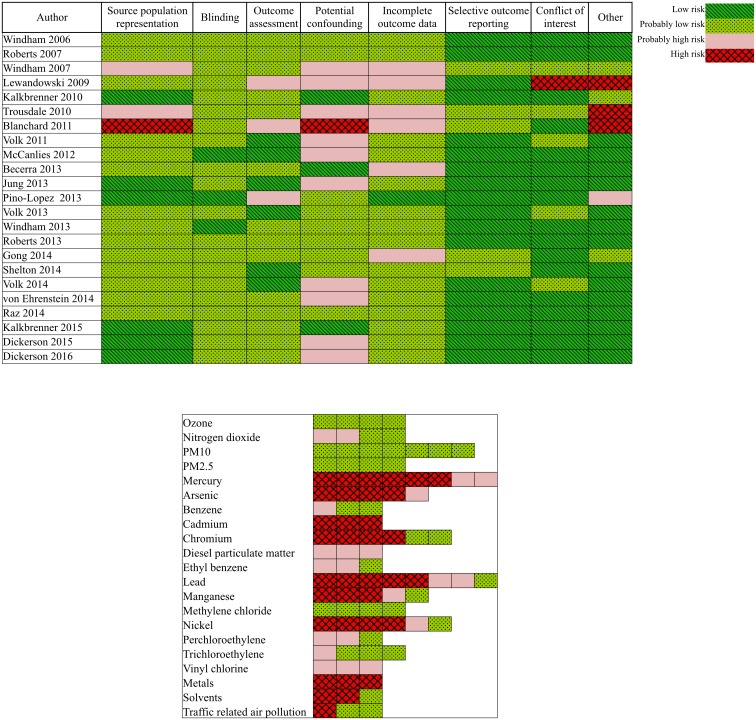
Risk of bias ratings for included human studies relevant to air pollution exposure and ASD outcome. A, All criteria except exposure assessment criteria. B, Exposure assessment criteria.

Overall, most studies were rated as “low” or “probably low” risk of bias in most domains other than the confounding and exposure assessment domains (Fig 2). Many of the studies rated as “probably high” (with one “high”) for potential confounding were due to the failure to adjust for many of the important confounders that we had established beforehand in our protocol. We rated exposure assessment risk of bias separately for each air pollutant or class of components within each study. Overall, we rated 103 different air pollutant chemicals (such as formaldehyde, PM_2.5_, ozone, etc.) and chemical classes (such as pesticides, fumigants, traffic-related pollutants, etc.). Only 21 of these chemicals or chemical classes were reported in ≥3 studies. Many of the contaminants that were ultimately rated as having “high” risk of exposure assessment bias used data from the US EPA National-scale Air Toxics Assessment (NATA), Toxic Release Inventory (TRI), or self-reported from surveys. We rated risk of exposure assessment bias for each air pollutant that used NATA data following published guidance that had already established the degree of confidence in each individual NATA contaminant estimate by comparing to monitored air pollution values [62, 63]. Following this guidance, our initial exposure assessment risk of bias ratings for NATA data were assigned as “probably low” for those with “higher confidence” (i.e., benzene, acrylonitrile, carbon tetrachloride, etc.), “probably high” for those with “medium” confidence (i.e., coke oven emissions, vinyl chloride, chloroform, ethylene dibromide, etc.), and “high” for those with “lower” confidence (i.e., arsenic compounds, beryllium compounds, cadmium compounds, lead compounds, etc.). All the air pollutants that were based on data modeled from Toxic Release Inventory (TRI) data were rated as “probably high” risk of exposure assessment based on concerns regarding the validity of extrapolating emission quantity data to individual or community-level exposures. Ultimately, only the studies involving air pollutants of PM_10_, PM_2.5_, ozone and methylene chloride were rated as “probably low” risk of bias (Fig 2b). Additional detail on individual study characteristics and risk of bias designation/rationale is presented in the S5 Table.

### Statistical analysis

Of the 23 included studies, six studies that measured PM_10_ and three that measured PM_2.5_ (a subset of the six with PM_10_ data) were amenable to meta-analyses. One additional included study [53] also reported effect estimates for both PM_10_ and PM_2.5_ but ultimately was not included in the meta-analysis because upon personal communication with the study authors [64] it was determined that the study population was a subset of the population reported in a previous article [52] that was already included in the meta-analyses. Furthermore, although included studies evaluating ozone and methylene chloride exposure were rated as “probably low” risk of bias and therefore potentially could have been incorporated into a meta-analysis, upon review there were limited studies (4 each) measuring exposure using varied metrics (environmental monitoring, emissions-based modeling, using occupation as surrogate for exposure) that were deemed too heterogeneous to combine into a meta-analysis.

All studies included in the meta-analyses measured PM exposure levels either through national or state air quality monitoring stations (i.e., US Environmental Protection Agency Air Quality System, California Air Resources Board monitor stations, or Taiwan EPA monitoring stations) or through historical emissions databases, combined with dispersion models to estimate residential levels of pollutants. Study population sizes ranged from 524–83,229. Study populations came from a variety of cohorts and regions, primarily in the U.S., such as the Nurses’ Health Study II, U.C. Davis’ Childhood Autism Risks from Genetics and the Environment (CHARGE) study, the Center for Diseases Control and Prevention’s (CDC) Autism and Developmental Disabilities Monitoring (ADDM) Network, Child and Adolescent Twin Study in Sweden (CATSS), or identified through insurance claims or state departments tracking ASD diagnoses. The majority of studies adjusted for maternal age, parental level of education, race/ethnicity, gender of child, household income, and some measure of socio-economic status. We contacted the corresponding author from four of the six included studies to request additional information and received responses from all four authors.

**Fig 3:**
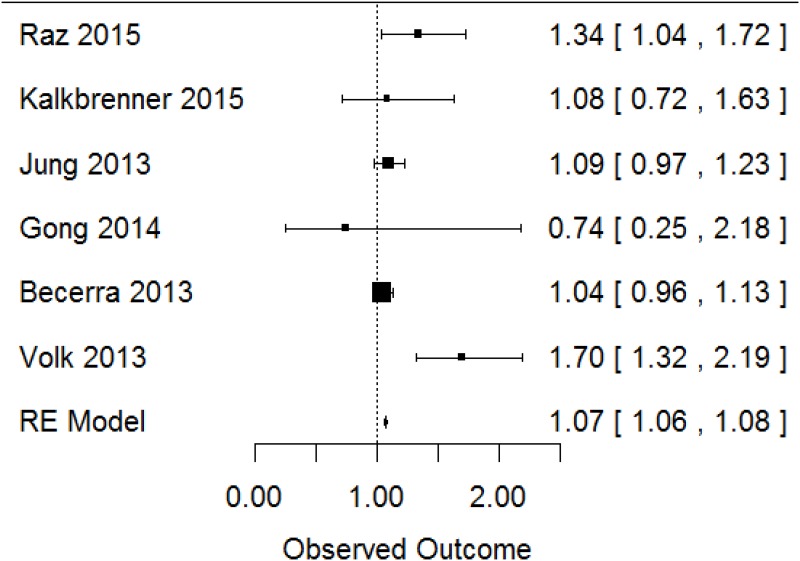
Meta-analysis of human studies; reported effect estimates [95% confidence interval] from individual studies (inverse-variance weighted, represented by size of rectangle) and overall pooled estimate from random effects (RE) model for PM_10_ exposure and ASD.

The initial meta-analyses of PM_10_ yielded a pooled OR = 1.20 (95% CI: [1.00, 1.42]). However, there was considerable statistical heterogeneity in the pooled estimate (I^2^ = 77%, p-value = 0.007). Although the overall number of studies was small, leading to uncertainty in the heterogeneity estimate, the 95% confidence interval for the I^2^ estimate (20–98%) suggested that heterogeneity was of concern. Using a leave-one-out analysis, we identified one study in particular [52] that was contributing to the majority of this heterogeneity. We contrasted design characteristics of this study with those of the other included studies to determine if the statistical heterogeneity could be explained by differences on a key characteristic; however, we concluded that the statistical heterogeneity was largely unexplained. We added in a hierarchical cluster structure to include this study as a separate cluster from all other studies, and this greatly reduced the unexplained heterogeneity (I^2^ = 2%, p-value = 0.41), indicating that with this clustered analysis statistical heterogeneity was of minimal concern. Based on this clustered meta-analysis, we found an overall effect estimate of OR = 1.07 (95% CI: [1.06, 1.08]) per 10-μg/m3 increase in PM_10_ (Fig 3).

We also conducted sensitivity analyses to assess the robustness of the positive relationship between PM_10_ exposure and risk for ASD. We estimated the hypothetical effect estimates needed from a single additional included study that would shift our meta-analysis to where: 1) the 95% confidence interval of the meta-analysis overlaps the value 1 (loses statistical significance), and 2) the summary effect estimate becomes less than 1. For both scenarios we assumed that the additional study would have a standard error of 0.04, equal to the smallest in our group of studies [39]. We found that an additional new study would need to have an effect estimate of OR = 1.03 to enlarge our confidence interval to overlap 1, and OR = 0.44 to shift our overall effect estimate to OR<1. The former OR estimate is reasonably within the range of that reported by existing studies (thus, it might be possibly for a new study to easily shift our interpretation of statistical significance of the overall effect estimate), but the OR required to shift the overall effect estimate to OR<1 seemed rather unlikely.

**Fig 4:**
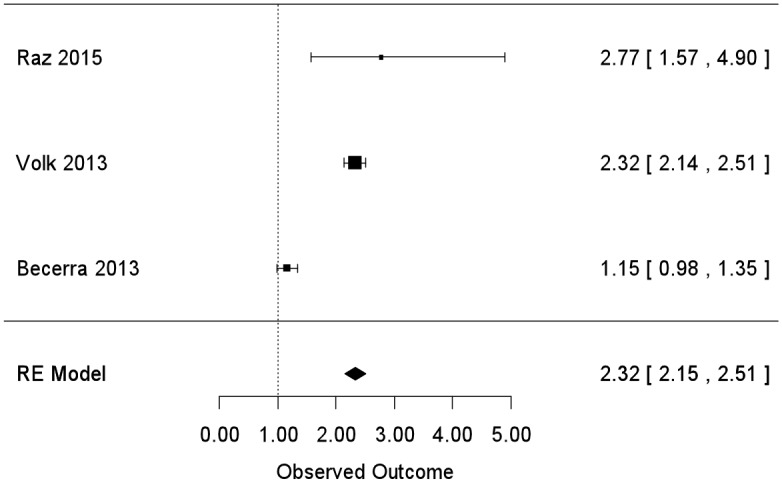
Meta-analysis of human studies; reported effect estimates [95% confidence interval] from individual studies (inverse-variance weighted, represented by size of rectangle) and overall pooled estimate from random effects (RE) model for PM_2.5_ exposure and ASD.

For PM_2.5,_ the summary effect estimate was OR = 1.88 (95% CI: [1.11, 3.20]) but, similar to PM_10_ analyses, we found evidence of considerable statistical heterogeneity (I^2^ = 96%; p-value<0.0001). Again using a leave-one-out analysis, we identified one study in particular [39] that was contributing to the majority of the heterogeneity and we could not identify particular methodological features driving the heterogeneity so we classified it as unexplained. Adding in a hierarchical cluster structure here greatly reduced the unexplained heterogeneity (I^2^ = 0%, p-value = 0.54), indicating that with this clustered analysis statistical heterogeneity was of minimal concern. Based on this clustered meta-analysis, we found a pooled effect estimate of OR = 2.32 (95% CI 2.15 to 2.51) per 10-μg/m3 increase in PM_2.5_ (Fig 4). Sensitivity analyses here (assuming the added hypothetical study would have a standard error of 0.04, equal to the smallest in our group of studies [39]) found that an added study would need an effect estimate of OR = 1.02 to enlarge the confidence interval to overlap 1, and an OR = 0.14 to shift the overall effect estimate to OR<1. The former OR estimate is outside the range of that reported by existing studies, but not unreasonably so; however, the OR required to shift the overall effect estimate to OR<1 seems quite unlikely.

For all other air pollutant chemicals or classes of chemicals with effect estimates reported for ≥3 studies, we generated scatter plots of reported data (S1 Fig). Odds ratio (OR) or relative risk (RR) estimates were plotted on the log-transformed scale by chemical and separated by categories of “general air pollutants” (including diesel particulate matter, nitrogen dioxide, and ozone), “industrial chemical pollutants” (including benzene, ethylbenzene, methylene chloride, perchloroethylene, styrene, toluene, trichloroethylene, and vinyl chloride), “heavy metal air pollutants” (including arsenic, cadmium, chromium, lead, nickel, manganese, and mercury). We also created a scatterplot for “pesticide air pollutants,”, combining all individual pesticides (e.g., acrolein) with grouped categories (e.g., organophosphates). We generally observed a trend towards positive effects (increasing exposure associated with increased autism risk), although there were limited data and confidence intervals commonly overlapped the null. The other air pollutants most consistently reporting statistically significant associations with of ASD included heavy metals with exposures assessed using NATA data. However, for many of these air pollutants, serious risk of bias existed for exposure assessment with one or more studies rated as “high” or “probably high” (Fig 2).

### Quality and strength of the overall body of evidence

We rated the quality of the human evidence as “moderate.” Our decisions leading to this rating were primarily based on the concern that many of the air pollutant chemicals or classes had exposure assessment methods that were rated “high” or “probably high” for risk of bias for exposure assessment methods (rated as between “0 to -1”) (Table 3). There was insufficient number of studies to utilize funnel plot analyses to assess publication bias quantitatively; so we based our decision to not downgrade for publication bias on the fact that we conducted a comprehensive search, found studies from the grey literature, and found studies of variable sizes, designs, and funding sources that had similar findings. We found insufficient evidence to upgrade the body of evidence. Ultimately, although there was concern regarding the risk of bias domain, we did not judge this to be sufficient enough to warrant downgrading the evidence and therefore remained at the initial “moderate” rating.

**Table 3:** Summary of rating quality and strength of the human evidence.

Category	Downgrades	Rationale
Risk of bias (ROB)	0 to -1	We rated overall risk of bias across all studies between 0 (no downgrade) and -1 (downgrade 1 level). Our rationale was that many studies had probably high or high risk of bias, mostly driven by exposure assessment methods. The lack of specificity across different pollutant classes was also a concern. Because of the heterogeneity in individual study ratings across all air pollutant contaminants, we found it impossible to assign one overall rating that would be relevant across all studies for all contaminants.
Indirectness	0	Exposures were not directly measured (lacking biomarkers or individual measurement of air pollutants); however this was accounted for in the ROB rating and no other areas of concern existed for indirectness.
Inconsistency	0	Effect estimates across studies were mostly positive (showing increased risk) and small (OR<2) and confidence intervals overlapped across studies for the majority of estimates.
Imprecision	0	No concern regarding the imprecision in effect estimates across studies.
Publication bias	0	The number of studies included in the meta-analysis were too small (i.e., <10) for a statistical evaluation of potential publication bias. We identified several findings from the grey literature through our comprehensive search, and two studies did find negative findings.
	**Upgrades**	
Large magnitude of effect	0	All of the studies found null or minimal effects only (i.e., OR<2).
Dose-response	0	Coauthors felt there was some evidence of a dose-response relationship, but not enough to warrant upgrading of the evidence.
Confounding minimizes effect	0	There was no evidence that residual confounding influenced results.
Overall Quality of Evidence	Moderate	Initial rating of “moderate” neither downgraded nor upgraded.
Overall Strength of Evidence	Limited	A positive relationship was observed between exposure and outcome where chance, bias, and confounding could not be ruled out with reasonable confidence. Confidence in the relationship is constrained by such factors as: the number, size, or quality of individual studies, or inconsistency of findings across individual studies. With more information, the observed effect could change, and this change may be large enough to alter the conclusion.

We rated the final overall strength of the evidence as “limited” (Table 2). While the meta-analysis suggested statistically significant effects for PM, these were based on very few studies and sensitivity analysis suggested that even a single added study within the range of ORs reported in the body of research to date could lead to a summary effect evidence that did not attain statistical significance (although an extreme OR estimate would be require to change the direction of the overall effect estimate to below 1). Further the occurrence of heterogeneity that lacked a clear methodological rationale is suggestive of randomness in the available data, was concerning to review authors, and this was influential in our final rating of “limited” overall evidence.

## Discussion

We conducted the first systematic review and meta-analysis of the body of human evidence to assess whether early life exposure to ambient air pollutants is associated with ASD. We concluded that there was “limited evidence of toxicity” for the association between early life exposure to air pollution as a whole and diagnosis of ASD. The strongest evidence supported an association between exposure to particulate matter and ASD. We utilized six robust studies (five case-control and one cohort) with minimal risk of bias concerns that represented a total of 9,557 children with autism and 143,997 controls reporting on PM_10_ exposure, and a subset of these that also reported on PM_2.5_. These studies reported effect estimates similar enough to be combined into a meta-analysis we found statistically significant pooled effect estimates for both PM components, with a stronger effect demonstrated for PM_2.5_ than for PM_10_. We identified statistical heterogeneity in our meta-analysis that could be minimized through incorporating a clustered data analysis structure, but not explained through differences in study design. We determined through sensitivity analysis that future studies comparable to the ones included in our review (i.e., with similar effect estimates and uncertainty) could potentially change the strength of the relationship between PM and ASD estimated from the meta-analysis. In other words, the effect of particulate matter on ASD may be stronger or weaker than these results indicate. However, we found that it would be unlikely that a future study would change the direction of the association between PM and ASD, for example moving air pollution from being a risk factor for ASD to being health protective for ASD. Generally, the other air pollutants that could not be combined into a meta-analysis (such as some of the metal air pollutants) supported positive and statistically significant effects, although the effects were generally small and not consistent. Collectively, these findings led to our conclusion that positive relationships were observed between air pollution exposures generally and ASD, but that chance, bias, and confounding could not be ruled out with reasonable confidence because of the limitations present in the available data. This met our definition of “limited evidence of toxicity” for the association between early life exposure to air pollution generally and ASD.

Our systematic review and meta-analysis had several strengths. This is the first example of applying a systematic approach and combining evidence into a meta-analysis to evaluate the body of evidence for all types of air pollutants collectively. The Navigation Guide approach is based on the GRADE principles [22] for rating the quality and strength of the evidence and incorporates similar criteria and considerations for evaluation, with slight modifications for application to environmental health evidence—in particular, the lack of randomized control human trials. The GRADE approach requires judicious consideration of the contribution of each study to addressing the study question, with general guidance to focus on the high-quality studies—in this case the evidence supporting the link between PM and ASD. However, evidence from all other air pollutant studies were also considered when evaluating the quality and strength of the evidence—for instance, determining whether the studies overall contributed direct evidence to answer the study question or whether the study estimates demonstrated a large magnitude of effect.

Our systematic review results were generally in concert with previous expert-based narrative reviews that addressed the association of ASD and environmental chemical exposures more broadly [18, 65–67]. The only prior review of air pollutants and neuropsychological development that followed a prescribed systematic methodology was limited to published English language studies appearing during or after 2012 and did not include a meta-analysis [68]. The authors of this review rated evidence based on IARC classifications and concluded that there was “sufficient” evidence for an association of ASD with PM_2.5._ The Navigation Guide classifications are based largely on the IARC classifications. However, our review additionally included a meta-analysis, the results of which further demonstrated an association between air pollution (PM) and ASD, but also revealed some uncertainties in the body of evidence that would not have been apparent except for the meta-analysis. The unexplained heterogeneity in the meta-analysis led us to conclude that the body of evidence fit the definition of “limited toxicity” better than the definition of “sufficient toxicity.”

Several newer studies have been published since the end-date of our search, including a European multi-site study combining different scale measurements of ASD symptoms, which reported no association between particulate matter or nitrogen dioxide and autistic traits [69] and two studies from a case-control sample in Pennsylvania, reporting an association between PM_2.5_ and ASD [70] and between some air toxics and ASD [71]. We do not know for certain how these studies would impact our review results without a formal evaluation of their risk of bias and integration of these studies into the overall rating of quality and strength of the evidence. However, we note that the effect estimates reported for PM in these studies generally fall within the range of those reported by the studies included in our meta-analysis.

Together, these findings support the hypothesis that early life exposure to air pollution may contribute to ASD. While some studies have suggested a strong genetic heritability contribution to ASD development, these do not fully explain the recent increases in ASD prevalence and thus environmental risk factors are recognized as playing a strong contribution to the increase in ASD [72–76]. Exposure to chemicals in air pollution may act through several potential pathophysiological mechanisms related to immune function, endocrine disruption, and epigenetic alterations [71]. These mechanisms vary greatly by chemical air pollutant—for instance, particulate pollutants (complex chemical mixtures solely defined by size, such PM_2.5_) are able to penetrate deep into the lungs and can enter blood circulation, where they can induce oxidative stress leading to inflammatory responses that subsequently result in the perturbation of neurodevelopment [77]. In contrast, other specific chemical constituents of air pollution, such as metals, may impact neurodevelopment of the developing fetus through direct exposure, or cause an elevation in inflammatory cytokines in the maternal circulation that subsequently impacts neurodevelopment [66, 78, 79]. While there are many outstanding questions about the nature and extent of the association of air pollution and ASD specifically, there is already strong evidence that certain air pollutants such as PM, lead, and mercury impact brain development [76, 80–82]. Our review identified research gaps that should inform future work on this topic. Further, we provide a concise statement of the strength of the evidence, which decision-makers and policymakers can use to integrate with other factors that are important in setting decisions, such as values and preferences about the outcome, alternatives to avoiding the outcome, and the costs and benefits of action.

### Advancing ASD and Air Pollution Research

The limitations identified in this body of evidence reveal gaps in the current scientific literature and point the way for optimizing future research related to the environmental contributors to ASD specifically, and air pollution in general. In particular, future research should account for the following:

1. ***Challenges in assessing exposure to the complex mixture that defines air pollution*.** The available evidence was not readily combinable in a meta-analysis because air pollution exposure was assessed through many different and non-standardized metrics and included data on over 100 different chemical components or surrogate measures of air pollution. While the PM data and several other pollutants were rated as low risk of bias (i.e., ozone, methylene chloride), only the PM data was sufficient for inclusion in a meta-analysis. Many of the other air pollutant exposures were classified as probably high or high risk of bias, with some being based on surrogate measures (i.e., distance to freeway) or lacking accounting for time-activity patterns or spatial accuracy. Studies also varied widely in terms of the methods (monitoring, modeling, etc.) and data source (NATA, TRI, etc.) used to assess exposure. Most studies assigned exposure based on birth address, which implies prenatal exposure, but may not reflect addresses earlier in pregnancy. Notably, some studies improved on this by obtaining a residence history from participants; future studies could potentially utilize tracing services to gather this important information. This is a generic challenge that will likely be present for the majority of air pollution studies, but recent advances in assessment methods such as use of portable personal sensors and exposomic technologies could make a significant impact. To increase the usefulness of study results to decision-making, investigators need to directly address these challenges and maximize the accuracy and applicability of exposure measurements to the target population.
2. ***Challenges related to study design*.** The included studies varied in the adjusted confounders considered, the timing of exposure or outcome measurement, and the method used for assessing the ASD outcome. Such heterogeneity makes it difficult to combine the results of multiple studies, as there is little empirical basis available to inform how variations in these study characteristics might impact the reported effect estimates. There is a need to establishing basic criteria for environmental studies of ASD based on an improved understanding of how different adjustment factors and timing or method of exposure and outcome in the same population impact effect estimated to maximize the potential for combining studies relevant to the same research question.
3. ***Challenges related to reporting*.** Many effect estimates were reported on different scales or categorized the exposures using different ranges. As such, with the exception of PM, where estimates could fairly easily be standardized, the body of evidence was disparate and largely not combinable. Future studies should increase availability of raw data or broaden reporting of effect estimates using different metrics to ensure statistical combinability in future meta-analyses.
4. ***Challenges related to unexplained heterogeneity*.** In meta-analyses, PM demonstrated statistically significant summary associations with ASD. We observed a larger summary effect estimate for PM_2.5_ (OR = 2.32) than for PM_10_ (OR = 1.20). Because smaller particles have the ability to penetrate into the circulatory system and are thought to be more biologically active [83–86], this pattern of association is not unexpected. Alternatively, the larger effect for PM_2.5_ could be related to the limited available sample of effect estimates as the Volk et al. [52] and Becerra et al. [39] studies, which generated the highest estimates for both PM_10_ and PM_2.5,_ comprised two of three included studies for PM_2.5_ but two of six included studies for PM_10._ There are possible study design characteristics that differed (for instance, the availability and quality of individual home addresses to estimate individual-level exposures), but it is difficult to assign an exact explanation for the observed heterogeneity in results. Unexplained heterogeneity is troubling but could be resolved with additional studies that could reduce overall variability of estimates and which provide insight into methodological reasons behind the heterogeneity.
5. ***The interplay between genes and the environment*.** One of the included studies reported an elevated risk of ASD only in those with a mutation in the MET tyrosine kinase receptor and those in the highest exposure level category [53]. While interactions between genes and the environment are known to play a key role in ASD, relatively few studies have been able to adequately power such analyses. The Volk et al. [53] study illustrates the importance of considering moderating variables to evaluate heterogeneity in examining ASD etiology. This will be an important consideration in future reviews as more data emerges on this topic.

### Synthesizing the Evidence for Decision-Making in Environmental Health

Our case study of applying the Navigation Guide systematic review method to synthesize the science related to ASD and air pollution underscored several key directions for evidence-based decision-making in environmental health.

1. ***Use of novel tools for assessing risk of bias in air pollution studies*.** Due to the complexity of assessing the risk of bias of air pollution exposure assessments, we developed a novel risk of bias tool and piloted its use for this study. This tool is now available [87] and can be adapted and implemented for evaluating exposure assessment of future systematic reviews and can also serve as a guide to strategically incorporate methods that reduce potential risks of bias in future air pollution studies.
2. ***The need for consistent and complete reporting of research results*.** Without the cooperation of individual study authors, it would have been impossible to complete the meta-analysis. This observation is consistent with our previous systematic reviews, [24, 27, 28, 30], and underscores the need for journal editors to routinely request consistent information from authors when manuscripts are accepted for publication to advance the capacity to conduct robust systematic reviews. To this end, several high-impact journals have already adopted the ARRIVE guidelines for animal studies (http://www.nc3rs.org.uk/ARRIVE/) [88, 89] or MOOSE guidelines for human observational studies [90]. Our experience supports these approaches, and we recommend reporting guidelines be expanded to consider other elements pertinent to high throughput *in vitro* studies and other types of experimental and observational evidence.
3. ***The need to mechanize and automate data synthesis*.** As with our previous systematic reviews, [24, 27, 28, 30], we found efficient ways to sort through a large number of studies captured through a broad search. Keys to this efficiency were implementing explicit predetermined inclusion/exclusion criteria and user-friendly software. We began the review using DRAGON software to perform title and abstract screening of studies but we found DistillerSR to be more flexible and easy to use and discontinued use of DRAGON. The development of increasingly capable software and other tools, including natural language processing and machine reading, [91] will be critical to advancing systematic reviews in clinical and environmental health.
4. ***Time sensitive nature of systematic reviews*.** We had completed our review but had not yet published our results when three new relevant studies were published [69]. This highlights the practice of establishing stopping dates for a review’s literature search, which is essential for assessments in regulatory and policy decision-making. The nature of scientific information means knowledge is always evolving, yet it is important to use stopping dates to evaluate what is known in the scientific literature at that time and use the information to make a decision based on this knowledge at hand. In the future we recommend re-running the search immediately before data analysis and planning for a very short time between data analysis and the review team’s rating of the quality and strength of the evidence. This will become increasingly feasible as more and more scientists are trained in the method and it becomes more efficient. It also highlights the need to conduct cumulative meta-analyses as more data become available, a common practice in the clinical sciences. A future research project could involve updating the search and investigating how new available studies might change our ratings and decision.

## Conclusion

In summary, we conducted the first systematic review and meta-analysis of the literature on the association between ASD and air pollution and concluded that there was “limited evidence of toxicity.” We found the strongest available evidence was supporting associations between PM and ASD, which was supported by the results of our meta-analysis. The available body of evidence on air pollution in general and ASD was wide, shallow, and except for PM, limited in their ability to strongly support a relationship if one exists. Our rating of the quality and strength of the evidence for this study question provides insight on the current state of the science but also the research gaps to address in future studies. Accurate measurement of human exposures to air pollutants during developmentally relevant time periods remains a key limitation in this research area, calling for continued work to improve air models, explore biomarkers, and pool and expand study samples to ameliorate effects from exposure measurement error. Identifying the environmental contributors to ASD and neurodevelopment in general is a critical unmet clinical and public health need, as recognized by a recent consensus statement published by leading scientific and medical experts, along with children’s health advocates, which identifies the importance of environment as a risk factor for neurodevelopment [92]. Furthermore, the strength of the scientific evidence is but one component of decision-making and other factors such as the co-benefits of reducing air pollution exposures and the severity of potential health outcomes should be taken into consideration when making policy and regulatory decisions. Our research findings and recommendations can support researchers, clinicians, impacted individuals, families, communities, policy-makers, and funding agencies in expediting the scientific discovery in this field as well as advancing evidence-based decision-making on how to take action to prevent future harm.

## Supporting Information

S1 Fig Reported effects estimates scatterplots. (DOCX)

S1 File PRISMA Checklist. (PDF)

S2 File Instructions for making risk of bias determinations. (DOCX)

S3 File List of excluded studies. (DOCX)

S1 Table Database-specific search terms. (DOCX)

S2 Table Toxicological websites and grey literature databases searched. (DOCX)

S3 Table Data extraction fields and description. (DOCX)

S4 Table Factors for evaluating the overall quality of a body of evidence. (DOCX)

S5 Table Individual study characteristics. (DOCX)
