# Supplementary material for: A Systematic Review and Meta-Analysis of Multiple Airborne Pollutants and Autism Spectrum Disorder
Source: PLoS One. 2016 Sep 21;11(9):e0161851. doi: 10.1371/journal.pone.0161851 (PMC5031428; doi:10.1371/journal.pone.0161851)
Supplement: S1 Fig — (DOCX) [file pone.0161851.s001.docx]

**S1 Fig. Reported effects estimates scatterplots**

Figure A. Reported effects estimates from studies reporting on general air pollutant and ASD associations


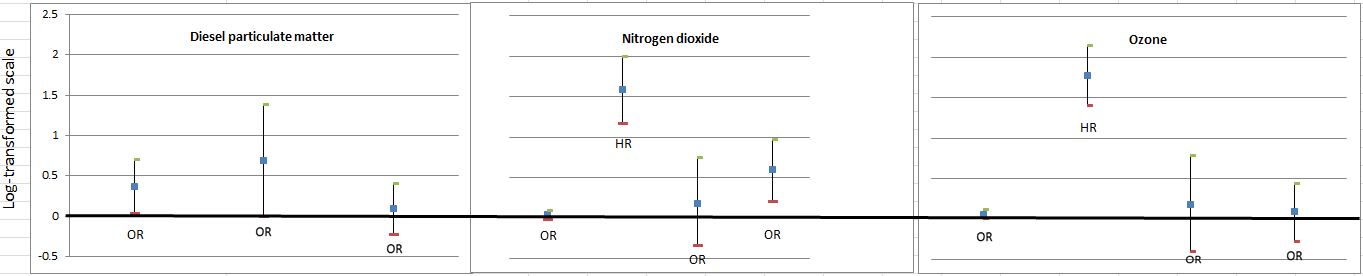


**References and source of data:**

*--US EPA National-scale Air Toxics Assessment (NATA) [1-3]*

*--Nearest air monitoring stations [4-6]*

*-- CALINE4 dispersion model and US EPA Air Quality System (AQS) [7]*

Figure B. Reported effect estimates from studies reporting on industrial chemical air pollutant and ASD associations


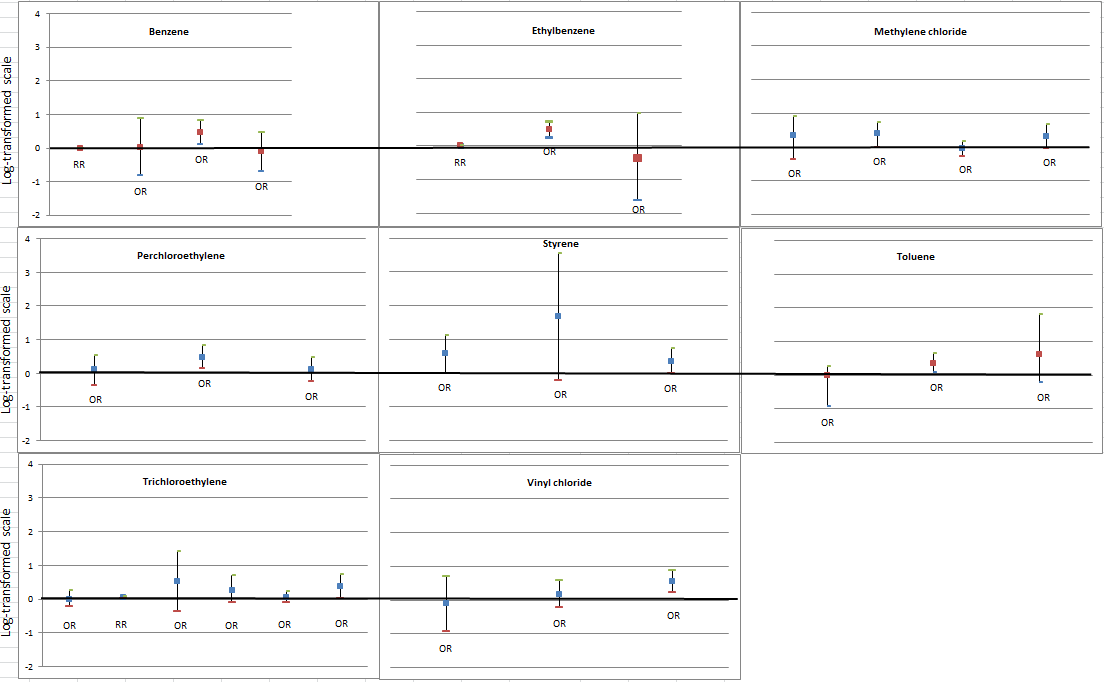


**References and source of data:**

*--Toxic Release Inventory (TRI) [8]*

*--Self-reported occupational exposures [9]*

*--Nearest air monitoring stations [10]*

*--US EPA National-scale Air Toxics Assessment (NATA) [1-3]*

Figure C. Reported effect estimates from studies reporting on heavy metal air pollutant and ASD associations


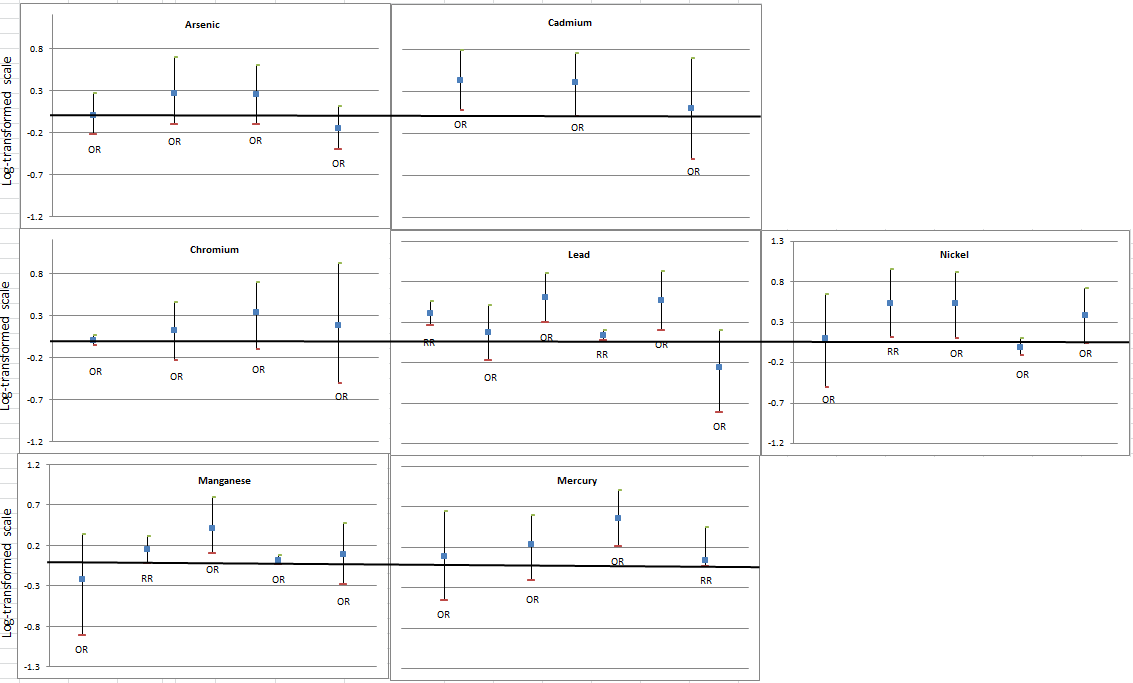


**References and source of data:**

*--Toxic Release Inventory (TRI) [8]*

*--Nearest air monitoring stations [10]*

*--US EPA National-scale Air Toxics Assessment (NATA) [1-3, 11]*

Figure D. Reported effect estimates from studies reporting on pesticide air pollutant^*^ and ASD associations


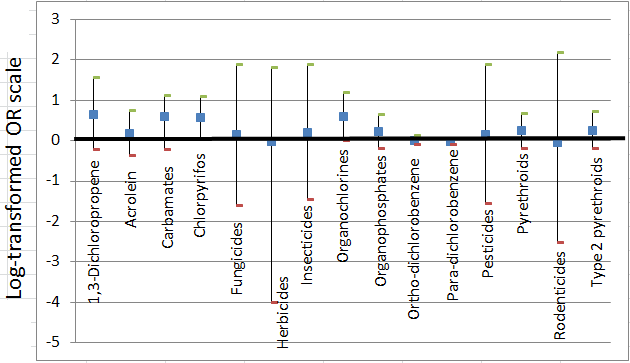


^*^Reported effect estimates for all individual and categories of pesticides were combined into one scatterplot

**References and source of data:**

*--Nearest air monitoring stations [10]*

*--US EPA National-scale Air Toxics Assessment (NATA) [3]*

*--California Department of Pesticide Regulation (DPR) [12, 13]*

*--Self-reported occupational exposures [9]*

**References**

1. Roberts AL, Lyall K, Hart JE, Laden F, Just AC, Bobb JF, et al. Perinatal air pollutant exposures and autism spectrum disorder in the children of Nurses' Health Study II participants. Environ Health Perspect. 2013;121(8):978-84. doi: 10.1289/ehp.1206187. PubMed PMID: 23816781; PubMed Central PMCID: PMC3734496.

2. Windham GC, King G, Roberts E, Croen LA, Grether J. Autism and distribution of hazardous air pollutants at birth in California. Epidemiology. 2007;18(Suppl 5):S174.

3. Kalkbrenner AE, Daniels JL, Chen JC, Poole C, Emch M, Morrissey J. Perinatal exposure to hazardous air pollutants and autism spectrum disorders at age 8. Epidemiology. 2010;21(5):631-41. doi: 10.1097/EDE.0b013e3181e65d76. PubMed PMID: 20562626; PubMed Central PMCID: PMC2989602.

4. Becerra TA, Wilhelm M, Olsen J, Cockburn M, Ritz B. Ambient air pollution and autism in Los Angeles county, California. Environ Health Perspect. 2013;121(3):380-6. doi: 10.1289/ehp.1205827. PubMed PMID: 23249813; PubMed Central PMCID: PMC3621187.

5. Jung CR, Lin YT, Hwang BF. Air Pollution and Newly Diagnostic Autism Spectrum Disorders: A Population-Based Cohort Study in Taiwan. PloS one. 2013;8(9). doi: 10.1371/journal.pone.0075510. PubMed PMID: WOS:000325218700093.

6. Volk HE, Kerin T, Lurmann F, Hertz-Picciotto I, McConnell R, Campbell DB. Autism Spectrum Disorder: Interaction of Air Pollution with the MET Receptor Tyrosine Kinase Gene. Epidemiology. 2014;2014(25).

7. Volk HE, Lurmann F, Penfold B, Hertz-Picciotto I, McConnell R. Traffic-related air pollution, particulate matter, and autism. JAMA psychiatry. 2013;70(1):71-7. doi: 10.1001/jamapsychiatry.2013.266. PubMed PMID: 23404082; PubMed Central PMCID: PMC4019010.

8. Lewandowski TA, Bartell SM, Yager JW, Levin L. An evaluation of surrogate chemical exposure measures and autism prevalence in Texas. Journal of Toxicology and Environmental Health, Part A. 2009;72(24):1592-603.

9. McCanlies EC, Fekedulegn D, Mnatsakanova A, Burchfiel CM, Sanderson WT, Charles LE, et al. Parental occupational exposures and autism spectrum disorder. Journal of autism and developmental disorders. 2012;42(11):2323-34.

10. von Ehrenstein OS, Aralis H, Cockburn M, Ritz B. In Utero Exposure to Toxic Air Pollutants and Risk of Childhood Autism. Epidemiology. 2014;25(6):851-8. doi: 10.1097/ede.0000000000000150. PubMed PMID: WOS:000343122000010.

11. Dickerson AS, Rahbar MH, Bakian AV, Bilder DA, Harrington RA, Pettygrove S, et al. Autism Spectrum Disorder prevalence and associations with air concentrations of lead, mercury, and arsenic. Pediatric and Perinatal Epidemiology. 2016;Submitted.

12. Shelton JF, Geraghty EM, Tancredi DJ, Delwiche LD, Schmidt RJ, Ritz B, et al. Neurodevelopmental disorders and prenatal residential proximity to agricultural pesticides: the CHARGE study. Environ Health Perspect. 2014;122(10):1103-10.

13. Roberts EM, English PB, Grether JK, Windham GC, Somberg L, Wolff C. Maternal residence near agricultural pesticide applications and autism spectrum disorders among children in the California Central Valley. Environmental Health Perspectives. 2007;115:1482-9.
