# Supplementary material for: A Systematic Review and Meta-Analysis of Multiple Airborne Pollutants and Autism Spectrum Disorder
Source: PLoS One. 2016 Sep 21;11(9):e0161851. doi: 10.1371/journal.pone.0161851 (PMC5031428; doi:10.1371/journal.pone.0161851)
Supplement: S3 File — (DOCX) [file pone.0161851.s004.docx]

**S2 File. List of excluded studies (n=1120).**

(1992). New Directions for Mental Health Services, No. 54. Neurobiological disorders in children and adolescents. New Directions for Mental Health Services; Neurobiological disorders in children and adolescents. E. Peschel, R. Peschel, C. W. Howe and J. W. Howe, Jossey-Bass Inc., Publishers {a}, 433 California St., San Francisco, California 94104, USA. 54: vi+138p.

(1999). "A research-oriented framework for risk assessment and prevention of Children's exposure to environmental toxicants." Environ Health Perspect 107(6): 510.

(2001). "NIEHS investigates links between children, the environment, and neurotoxicity." Environmental Health Perspectives 109(6): A258-A260.

(2001). "Recent discoveries affecting drug therapy." Drug Benefit Trends 13(6): 62+65-66.

(2002). "Autism - High blood metal levels may be clue to autism." Chemistry & Industry(15): 5-5.

(2003). "Autism - No metal disease?" Deutsche Apotheker Zeitung 143(47): 70-71.

(2005). Bioactive Natural Products (Part K). Bioactive Natural Products. A. U. Rahman, Elsevier Science Bv, Sara Burgerhartstraat 25, Po Box 211, 1000 Ae Amsterdam, Netherlands. 30.

(2005). "Eye movement autism diagnosis, Down's, genes, Ca, CPR, rating hospitals, ?Say "sorry", making money, pork, overcriminalization: You're all felons! TSA, no more coeds, tires, gasoline, lose your wheels?" Binocular Vision and Strabismus Quarterly 20[[1](#_ENREF_1)]: 226-244.

(2005). "First reports of adverse drug reactions in recent weeks." Drugs and Therapy Perspectives 21(5): 18-20.

(2005). "A mercurial debate over autism." Nat Neurosci 8[[1](#_ENREF_1)]: 1123.

(2006). Neurotoxicity and Developmental Disabilities. Neurotoxicity and Developmental Disabilities. P. W. Davidson, G. J. Myers and B. Weiss, Elsevier Academic Press Inc, 525 B Street, Suite 1900, San Diego, Ca 92101-4495 USA. 30.

(2007). "Autistic spectrum disorder: No causal relationship with vaccines." Paediatrics and Child Health 12(5): 393-398.

(2007). "Silencing debate over autism." Nature Neuroscience 10(5): 531.

(2007). "Thimerosal: updated statement. An Advisory Committee Statement (ACS)." Can Commun Dis Rep 33(Acs-6): 1-13.

(2008). "The effects of plants on autism, pregnancy and thyroid." Phytotherapie 6[[1](#_ENREF_1)]: 46.

(2010). Environmental Heavy Metal Pollution and Effects on Child Mental Development: Risk Assessment and Prevention Strategies. Environmental Heavy Metal Pollution and Effects on Child Mental Development: Risk Assessment and Prevention Strategies. L. I. Simeonov, M. V. Kochubovski and B. G. Simeonova, Springer, Po Box 17, 3300 Aa Dordrecht, Netherlands.

(2011). "In this issue." Chemical Research in Toxicology 24[[1](#_ENREF_1)]: 1787-1788.

(2013). "5th International Congress on Psychopharmacology & International Symposium on Child and Adolescent Psychopharmacology." Bulletin of Clinical Psychopharmacology 23: S1.

(2014). "Erratum to Perinatal air pollutant exposures and autism spectrum disorder in the children of nurses' health study II participants (Environ Health Perspect, (2013), 121, (978-984), 10.1289/ehp.1206187)." Environmental Health Perspectives 122(6): A152.

Abbott, L. C. and S. S. Nahm (2004). "Neuronal nitric oxide synthase expression in cerebellar mutant mice." Cerebellum 3[[2](#_ENREF_2)]: 141-151.

Abdullah, M. M., et al. (2012). "Heavy metal in children's tooth enamel: related to autism and disruptive behaviors?" J Autism Dev Disord 42(6): 929-936.

Abrams, P. (2012). "Editorial Comment: Re: Brubaker L. Conflict of interest: What is the role of our professional societies? Neurourol Urodyn 2012;31:1217-1218." Neurourology and Urodynamics 31[[1](#_ENREF_1)]: 1219-1220.

Abu Shmais, G. A., et al. (2012). "Mechanism of nitrogen metabolism-related parameters and enzyme activities in the pathophysiology of autism." Journal of Neurodevelopmental Disorders 4: 11.

Adams, J. B., et al. (2013). "Toxicological status of children with autism vs. neurotypical children and the association with autism severity." Biol Trace Elem Res 151(2): 171-180.

Adams, J. B., et al. (2009). "Safety and efficacy of oral DMSA therapy for children with autism spectrum disorders: Part A--medical results." BMC Clin Pharmacol 9: 16.

Adams, J. B., et al. (2009). "Safety and efficacy of oral DMSA therapy for children with autism spectrum disorders: part B - behavioral results." BMC Clin Pharmacol 9: 17.

Adams, J. B., et al. (2009). "The severity of autism is associated with toxic metal body burden and red blood cell glutathione levels." J Toxicol 2009: 532640.

Adams, J. B., et al. (2006). "Analyses of toxic metals and essential minerals in the hair of Arizona children with autism and associated conditions, and their mothers." Biol Trace Elem Res 110[[2](#_ENREF_2)]: 193-209.

Adams, J. B., et al. (2007). "Mercury, lead, and zinc in baby teeth of children with autism versus controls." J Toxicol Environ Health A 70(12): 1046-1051.

Addae, C., et al. (2013). "Effect of the environmental pollutant hexachlorobenzene (HCB) on the neuronal differentiation of mouse embryonic stem cells." International Journal of Environmental Research and Public Health 10(10): 5244-5256.

Addae, C. A. and E. Martinez-Ceballos (2010). "Effect of hexachlorobenzene (HCB) on the neuronal differentiation of mouse embryonic stem cells." Molecular Biology of the Cell 21(24).

Agostoni, C. and D. Turck (2011). "Is cow's milk harmful to a child's health?" J Pediatr Gastroenterol Nutr 53(6): 594-600.

Ahn, K. C., et al. (2006). "Immunological assay for BDE-47 (2,2 ',4,4 '-tetrabromodiphenyl ether) in biological samples." Abstracts of Papers American Chemical Society 232: 540.

Akins, M. R., et al. (2009). "Presynaptic translation: stepping out of the postsynaptic shadow." Frontiers in Neural Circuits 3: 7.

Akins, R. S., et al. (2010). "Complementary and alternative medicine in autism: An evidence-based approach to negotiating safe and efficacious interventions with families." Neurotherapeutics 7[[2](#_ENREF_2)]: 307-319.

Akyol, O. and H. Alacam (2007). "Metabolic parameters of the NO pathway in relationship to psychiatric disorders." European Neuropsychopharmacology 17(Suppl. 4): S190.

Akyol, O., et al. (2004). "Nitric oxide as a physiopathological factor in neuropsychiatric disorders." In Vivo 18[[2](#_ENREF_2)]: 377-390.

Alabdali, A., et al. (2014). "A key role for an impaired detoxification mechanism in the etiology and severity of autism spectrum disorders." Behav Brain Funct 10: 14.

Al-Ayadhi, L. Y. (2005). "Heavy metals and trace elements in hair samples of autistic children in central Saudi Arabia." Neurosciences (Riyadh) 10[[2](#_ENREF_2)]: 213-218.

Albizzati, A., et al. (2012). "Normal concentrations of heavy metals in autistic spectrum disorders." Minerva Pediatr 64[[1](#_ENREF_1)]: 27-31.

Alexander, J. J., et al. (2008). "Amoxicillin for postexposure inhalational anthrax in pediatrics: Rationale for dosing recommendations." Pediatric Infectious Disease Journal 27[[1](#_ENREF_1)]: 955-957.

Al-Farsi, Y. M., et al. (2013). "Levels of heavy metals and essential minerals in hair samples of children with autism in Oman: a case-control study." Biol Trace Elem Res 151(2): 181-186.

Allen, J. L., et al. (2014). "Early postnatal exposure to ultrafine particulate matter air pollution: persistent ventriculomegaly, neurochemical disruption, and glial activation preferentially in male mice." Environ Health Perspect 122[[1](#_ENREF_1)]: 939-945.

Almogren, A., et al. (2013). "Levels of heavy metal and trace element among children with autism spectrum disorders." Current Pediatric Research 17(2): 79-83.

Al-Salehi, S. M. and M. Ghaziuddin (2009). "G6PD deficiency in autism: a case-series from Saudi Arabia." Eur Child Adolesc Psychiatry 18[[1](#_ENREF_1)]: 227-230.

Al-Yafee, Y. A., et al. (2011). "Novel metabolic biomarkers related to sulfur-dependent detoxification pathways in autistic patients of Saudi Arabia." Bmc Neurology 11: 9.

Amimoto, Y., et al. (2012). "Lung sound analysis in a patient with vocal cord dysfunction and bronchial asthma." J Asthma 49[[2](#_ENREF_2)]: 227-229.

Amin, O. (2012). "HEAVY METALS AND AND TRACE ELEMENTS IN HAIR AND URINE OF A SAMPLE OF ARAB CHILDREN WITH AUTISTIC SPECTRUM DISORDER." European Psychiatry 27: 1.

Amin, O. R. (2012). "Efficacy of oral dimercaptosuccinic acid (DMSA) therapy in a sample of arab children with autistic spectrum disorder." European Psychiatry 27.

Andari, E., et al. (2010). "Promoting social behavior with oxytocin in high-functioning autism spectrum disorders." Proc Natl Acad Sci U S A 107[[1](#_ENREF_1)]: 4389-4394.

Andari, E., et al. (2012). "Oxytocin's fingerprints in social deficits of autism spectrum disorders." Encephale 38[[1](#_ENREF_1)]: S18.

Anderson, G. and M. Maes (2014). "Redox regulation and the autistic spectrum: Role of tryptophan catabolites, immuno-inflammation, autoimmunity and the amygdala." Current Neuropharmacology 12(2): 148-167.

Anderson, R. J., et al. (2003). Putting tryptophan in the spotlight. Hamilton, B C Decker Inc (Canada).

Anderson, R. J., et al. (2002). "On the biochemical basis of autism." Journal of Peptide Science 8(Supplement): S181.

Andreoli, L., et al. (2012). "Pregnancy implications for systemic lupus erythematosus and the antiphospholipid syndrome." Journal of Autoimmunity 38(2-3): J197-J208.

Anonymous (2000). "Disturbing behavior: Neurotoxic effects in children." Environmental Health Perspectives 108(6): A262-A267.

Anonymous (2004). "Abstracts & Short Articles of the 6th Biennial International Symposium on the Bi-Digital O-Ring Test - August 6-8, 2004 - Ibuka Auditorium (International Conference Hall) - Waseda University, Tokyo, Japan." Acupuncture & Electro-Therapeutics Research 29(3-4): 247-303.

Anonymous (2007). "32nd Annual Meeting of the Human-Biology-Association, Philadelphia, PA, USA, March 28 -29, 2007." American Journal of Human Biology 19(2): 246-294.

Anonymous (2009). "50th Annual Meeting of the European-Society-for-Paediatric-Reseach, Hamburg, GERMANY, October 09 -12, 2009." Acta Paediatrica 98(Suppl. 460): 1-278.

Anonymous (2010). "40th Annual Meeting of the Association-for-Applied-Psychophysiology-and-Biofeedback, Albuquerque, NM, USA, April 01 -04, 2009." Applied Psychophysiology and Biofeedback 35(2): 177-187.

Anonymous (2011). "27th Annual International Symposium on Acupuncture and Electro-Therapeutics and Integrated Medicine, New York, NY, USA, October 21 -24, 2011." Acupuncture & Electro-Therapeutics Research 36(3-4): 331-366.

Anonymous (2011). "Conference on the Mitochondrial Medicine, Scottsdale, AZ, USA, June 16 -19, 2010." Mitochondrion (Kidlington) 11[[1](#_ENREF_1)]: 638-677.

Anonymous (2012). "52nd Annual Meeting of the Japanese-Teratology-Society, Tokyo, JAPAN, July 06 -08, 2012." Congenital Anomalies 52[[1](#_ENREF_1)]: A1-A21.

Anonymous (2014). "Changes to the masthead." Neurotoxicology and Teratology 41: A1.

Arroyo, H. A. and M. C. Fernandez (2013). "[Environmental toxic and its effect on neurodevelopment]." Medicina (B Aires) 73 Suppl 1: 93-102.

Aschner, M. (2008). "Re: Blood levels of mercury are related to diagnosis of autism: a reanalysis of an important data set." J Child Neurol 23[[1](#_ENREF_1)]: 463; author reply 463-465.

Aschner, M., et al. (2006). "Metallothioneins: mercury species-specific induction and their potential role in attenuating neurotoxicity." Exp Biol Med (Maywood) 231[[1](#_ENREF_1)]: 1468-1473.

Aschner, M. and S. J. Walker (2002). "The neuropathogenesis of mercury toxicity." Mol Psychiatry 7 Suppl 2: S40-41.

Assefa, S., et al. (2012). "Inorganic mercury exposure in prairie voles (Microtus ochrogaster) alters the expression of toll-like receptor 4 and activates inflammatory pathways in the liver in a sex-specific manner." Human & Experimental Toxicology 31[[1](#_ENREF_1)]: 376-386.

Audouze, K. and P. Grandjean (2011). "Application of computational systems biology to explore environmental toxicity hazards." Environ Health Perspect 119(12): 1754-1759.

Austin, D. (2008). "An epidemiological analysis of the 'autism as mercury poisoning' hypothesis." International Journal of Risk and Safety in Medicine 20[[2](#_ENREF_2)]: 135-142.

Austin, D. W. and K. Shandley (2008). "An investigation of porphyrinuria in Australian children with autism." Journal of Toxicology and Environmental Health - Part A: Current Issues 71(20): 1349-1351.

Autism Kiykim, E., et al. (2012). "Zinc/copper metabolism in." Journal of Inherited Metabolic Disease 35[[1](#_ENREF_1)]: S139.

Autrup, H. (2005). "Children as a risk group - report from an ecotoc-taskforce." Toxicology Letters (Shannon) 158(Suppl. 1): S32.

Awale, P. S., et al. (2011). "Immune contribution to the valproic acid model of autism." Society for Neuroscience Abstract Viewer and Itinerary Planner 41.

Aykut, D. S., et al. (2012). "Nitric Oxide and Asymmetrical Dimethylarginine Levels in Acute Mania." Klinik Psikofarmakoloji Bulteni-Bulletin of Clinical Psychopharmacology 22[[1](#_ENREF_1)]: 10-16.

Ayres, K. M., et al. (2013). "THE USE OF MOBILE TECHNOLOGIES TO ASSIST WITH LIFE SKILLS/INDEPENDENCE OF STUDENTS WITH MODERATE/SEVERE INTELLECTUAL DISABILITY AND/OR AUTISM SPECTRUM DISORDERS: CONSIDERATIONS FOR THE FUTURE OF SCHOOL PSYCHOLOGY." Psychology in the Schools 50[[2](#_ENREF_2)]: 259-271.

Aziz, A., et al. (2011). "DIA1R is an X-linked gene related to Deleted In Autism-1." PLoS One 6[[1](#_ENREF_1)]: e14534.

Backx, M. and A. Freedman (2009). "Immunization." Medicine 37(10): 529-534.

Baecker, T., et al. (2014). "Loss of COMMD1 and copper overload disrupt zinc homeostasis and influence an autism-associated pathway at glutamatergic synapses." Biometals 27[[1](#_ENREF_1)]: 715-730.

Bagasra, O., et al. (2013). "Role of perfumes in pathogenesis of autism." Med Hypotheses 80(6): 795-803.

Bagni, C., et al. (2011). "The cytoplasmic FMRP interacting protein 1 CYFIP1 links fragile X syndrome to other neuro developmental and psychiatric disorders." Journal of Neurochemistry 118: 15.

Bakare, M. O. and K. M. Munir (2011). "Autism spectrum disorders (ASD) in Africa: a perspective." Afr J Psychiatry (Johannesbg) 14[[2](#_ENREF_2)]: 208-210.

Baker, J. P. (2008). "Baker responds." American Journal of Public Health 98[[1](#_ENREF_1)]: 1350-1351.

Baker, J. P. (2008). "Mercury, vaccines, and autism: One controversy, three histories." American Journal of Public Health 98(2): 244-253.

Bale Jr, J. F. (2004). "Neurologic complications of immunization." Journal of Child Neurology 19(6): 405-412.

Balmer, N. V. and M. Leist (2014). "Epigenetics and transcriptomics to detect adverse drug effects in model systems of human development." Basic and Clinical Pharmacology and Toxicology 115[[1](#_ENREF_1)]: 59-68.

Banji, D., et al. (2014). "Carbosulfan exposure during embryonic period can cause developmental disability in rats." Environmental Toxicology and Pharmacology 38[[1](#_ENREF_1)]: 230-238.

Baron, M. (2005). "The autism industry." New Scientist 188(2519): 19-19.

Barrett, J. R. (2002). "On a growth curve: Children's environmental health centers." Environmental Health Perspectives 110(10): A570-A572.

Bartell, S. (2012). "The use and misuse of exposure biomarkers in epidemiology." Epidemiology 23(5): S69.

Bartell, S. and T. Lewandowski (2011). "Bias from administrative censoring in ecological analyses of autism, and a bayesian solution." Epidemiology 22: S176.

Bartell, S. M. and T. A. Lewandowski (2011). "Administrative censoring in ecological analyses of autism and a Bayesian solution." J Environ Public Health 2011: 202783.

Barthelemy, C., et al. (1991). "EVALUATION OF BEHAVIORAL DISORDERS IN AUTISM IN THE CHILD QUANTITATIVE CLINICAL DATA AND FUNCTIONAL CEREBRAL IMAGING." Archives Internationales de Physiologie de Biochimie et de Biophysique 99(5): A94.

Bartolome, A., et al. (2005). "Fatty acid sensor for low-cost lifetime-assisted ratiometric sensing using a fluorescent fatty acid binding protein." Anal Biochem 345[[1](#_ENREF_1)]: 133-139.

Bate, S., et al. (2014). "Intranasal inhalation of oxytocin improves face processing in developmental prosopagnosia." Cortex 50: 55-63.

Baxter, A. J. and E. P. Krenzelok (2008). "Pediatric fatality secondary to EDTA chelation." Clin Toxicol (Phila) 46(10): 1083-1084.

Becerra, T. A., et al. (2014). "Autism Spectrum Disorders and Race, Ethnicity, and Nativity: A Population-Based Study." Pediatrics 134[[1](#_ENREF_1)]: E63-E71.

Beck, V. and B. Rimland (2001). Method of using secretin for treating autism, Repligen Corporation.

Bedford, H. E. and D. A. C. Elliman (2002). "The adverse effects of vaccines - Fact and fiction." Current Paediatrics 12[[1](#_ENREF_1)]: 62-66.

Bellinger, D. C. (2012). "Comparing the population neurodevelopmental burdens associated with children's exposures to environmental chemicals and other risk factors." NeuroToxicology 33[[1](#_ENREF_1)]: 641-643.

Bellinger, D. C. (2012). "A strategy for comparing the contributions of environmental chemicals and other risk factors to neurodevelopment of children." Environmental Health Perspectives 120[[1](#_ENREF_1)]: 501-507.

Bello, S. C. (2007). "Autism and environmental influences: Review and commentary." Reviews on Environmental Health 22(2): 139-156.

Bengmark, S. (2013). "Gut microbiota, immune development and function." Pharmacological Research 69[[1](#_ENREF_1)]: 87-113.

Bennett, M., et al. (2010). "Hyperbaric oxygen therapy and neurological disease." Undersea & hyperbaric medicine : journal of the Undersea and Hyperbaric Medical Society, Inc 37(5): 371-373; author reply 373-374.

Berghella, A. M., et al. (2012). "The discovery of how gender influences age immunological mechanisms in health and disease, and the identification of ageing gender-specific biomarkers, could lead to specifically tailored treatment and ultimately improve therapeutic success rates." Immunity and Ageing 9.

Berko, A. D., et al. (2012). "Developing a PC12 assay to examine calcium signaling in the regulation of nitric oxide: Role of NCS-1?" Society for Neuroscience Abstract Viewer and Itinerary Planner 42.

Berman, R. F., et al. (2008). "Low-level neonatal thimerosal exposure: further evaluation of altered neurotoxic potential in SJL mice." Toxicol Sci 101(2): 294-309.

Bernard, S., et al. (2008). "Re: Miles & Takahashi paper on RHIg and autism [[1](#_ENREF_1)]." American Journal of Medical Genetics, Part A 146[[2](#_ENREF_2)]: 405-406.

Bernard, S., et al. (2001). "Autism: A novel form of mercury poisoning." Medical Hypotheses 56[[1](#_ENREF_1)]: 462-471.

Bernard, S., et al. (2002). "The role of mercury in the pathogenesis of autism." Mol Psychiatry 7 Suppl 2: S42-43.

Berry-Kravis, E., et al. (2013). "Outcome Measures for Clinical Trials in Fragile X Syndrome." Journal of Developmental and Behavioral Pediatrics 34(7): 508-522.

Berwick, D. C. and K. Harvey (2013). "LRRK2: An eminence grise of Wnt-mediated neurogenesis?" Frontiers in Cellular Neuroscience(MAY).

Besancenot, J. P., et al. (2011). "Has allergenic pollen an impact on non-allergic diseases?" European Annals of Allergy and Clinical Immunology 43[[2](#_ENREF_2)]: 69-76.

Bettecken, T., et al. (2010). "Complex phenotype of male patient with a large duplication of Xp22." Medizinische Genetik 22[[1](#_ENREF_1)]: 133.

Betts, K. S. (2014). "Clues to autistic behaviors: Exploring the role of endocrine disruptors." Environmental Health Perspectives 122(5): A137.

Beyer, S. (2012). "The progress towards integrated employment in the UK." Journal of Vocational Rehabilitation 37[[2](#_ENREF_2)]: 185-194.

Bhargava, S. and S. C. Tyagi (2014). "Nutriepigenetic regulation by folate-homocysteine-methionine axis: A review." Molecular and Cellular Biochemistry 387(1-2): 55-61.

Bigham, M., et al. (2002). "Exposure to thimerosal in vaccines used in Canadian infant immunization programs, with respect to risk of neurodevelopmental disorders." Can Commun Dis Rep 28[[1](#_ENREF_1)]: 69-80.

Bihari, T. (2006). "Assessing CAM options for treating autism." Alternative and Complementary Therapies 12(5): 233-237.

Birnbaum, L. S. (2013). "When environmental chemicals act like uncontrolled medicine." Trends in Endocrinology and Metabolism 24(7): 321-323.

Bjorklund, G. (2013). "The role of zinc and copper in autism spectrum disorders." Acta Neurobiol Exp (Wars) 73(2): 225-236.

Black, J. (2002). "Letter from London [[1](#_ENREF_1)]." National Medical Journal of India 15(6): 355.

Blake, V. "The National Childhood Vaccine Injury Act and the supreme court's interpretation." Virtual Mentor.

Blaucok-Busch, E., et al. (2012). "Efficacy of DMSA Therapy in a Sample of Arab Children with Autistic Spectrum Disorder." Maedica (Buchar) 7[[2](#_ENREF_2)]: 214-221.

Blaurock-Busch, E., et al. (2012). "Toxic Metals and Essential Elements in Hair and Severity of Symptoms among Children with Autism." Maedica (Buchar) 7[[1](#_ENREF_1)]: 38-48.

Blaurock-Busch, E., et al. (2011). "Heavy metals and trace elements in hair and urine of a sample of arab children with autistic spectrum disorder." Maedica (Buchar) 6[[1](#_ENREF_1)]: 247-257.

Blaxill, M. F. (2004). "Concerns continue over mercury and autism." Am J Prev Med 26[[1](#_ENREF_1)]: 91; reply 91-92.

Blaxill, M. F., et al. (2004). "Thimerosal and autism? A plausible hypothesis that should not be dismissed." Medical Hypotheses 62(5): 788-794.

Blaylock, R. L. (2009). "A possible central mechanism in autism spectrum disorders, part 2: immunoexcitotoxicity." Altern Ther Health Med 15[[1](#_ENREF_1)]: 60-67.

Blaylock, R. L. (2009). "A possible central mechanism in autism spectrum disorders, part 3: the role of excitotoxin food additives and the synergistic effects of other environmental toxins." Altern Ther Health Med 15(2): 56-60.

Blaylock, R. L. and A. Strunecka (2009). "Immune-glutamatergic dysfunction as a central mechanism of the autism spectrum disorders." Current Medicinal Chemistry 16(2): 157-170.

Block, M. L., et al. (2012). "The outdoor air pollution and brain health workshop." NeuroToxicology 33(5): 972-984.

Bloom, P. and D. S. Weisberg (2007). "Childhood origins of adult resistance to science." Science 316(5827): 996-997.

Blossom, S. J., et al. (2008). "Developmental exposure to trichloroethylene promotes CD4(+) T cell differentiation and hyperactivity in association with oxidative stress and neurobehavioral deficits in MRL+/+ mice." Toxicology and Applied Pharmacology 231[[2](#_ENREF_2)]: 344-353.

Blossom, S. J., et al. (2012). "Postnatal exposure to trichloroethylene alters glutathione redox homeostasis, methylation potential, and neurotrophin expression in the mouse hippocampus." Neurotoxicology 33(6): 1518-1527.

Blum, J. D. and N. Talib (2006). "Balancing individual rights versus collective good in public health enforcement." Medicine and Law 25(2): 273-281.

Blume, S. (2006). "Anti-vaccination movements and their interpretations." Social Science and Medicine 62[[2](#_ENREF_2)]: 628-642.

Bohacek, J. and I. M. Mansuy (2013). "Epigenetic inheritance of disease and disease risk." Neuropsychopharmacology 38[[1](#_ENREF_1)]: 220-236.

Bolton, P., et al. (1992). "Season of birth: Issues, approaches and findings for autism." Journal of Child Psychology and Psychiatry and Allied Disciplines 33[[2](#_ENREF_2)]: 509-530.

Bond, T. and H. Hakonarson (2013). "Ask the Experts Pharmacogenomics and genome-wide association studies." Pharmacogenomics 14[[1](#_ENREF_1)]: 365-368.

Bondy, S. C. and A. Campbell (2005). "Developmental neurotoxicology." Journal of Neuroscience Research 81(5): 605-612.

Bonhomme, J. (2010). "Newinsights: The importance of father's health to the health of children." Journal of Men's Health 7[[2](#_ENREF_2)]: 301.

Borchers, A. T., et al. (2002). "Vaccines, viruses, and voodoo." Journal of Investigational Allergology and Clinical Immunology 12[[2](#_ENREF_2)]: 155-168.

Borck, G., et al. (2008). "Clinical, cellular, and neuropathological consequences of AP1S2 mutations: further delineation of a recognizable X-linked mental retardation syndrome." Hum Mutat 29(7): 966-974.

Boso, M., et al. (2006). "Neurophysiology and neurobiology of the musical experience." Funct Neurol 21[[1](#_ENREF_1)]: 187-191.

Bottos, A., et al. (2011). "Neurexins and neuroligins: synapses look out of the nervous system." Cellular and Molecular Life Sciences 68(16): 2655-2666.

Bourlioux, P. (2014). "[Current view on gut microbiota]." Ann Pharm Fr 72[[1](#_ENREF_1)]: 15-21.

Bowton, E., et al. (2014). "SLC6A3 coding variant Ala559Val found in two autism probands alters dopamine transporter function and trafficking." Transl Psychiatry 4: e464.

Bradstreet, J. J., et al. (2010). "Biomarker-guided interventions of clinically relevant conditions associated with autism spectrum disorders and attention deficit hyperactivity disorder." Altern Med Rev 15[[1](#_ENREF_1)]: 15-32.

Brady, L. S., et al. (2009). "NIMH Initiatives to Facilitate Collaborations Among Industry, Academia, and Government for the Discovery and Clinical Testing of Novel Models and Drugs for Psychiatric Disorders." Neuropsychopharmacology 34[[1](#_ENREF_1)]: 229-243.

Brask, B. H., et al. (1987). "A CASE OF PERVASIVE DEVELOPMENTAL DISORDER IN A BOY WITH EXTREMELY HIGH LEAD LEVELS IN DECIDUOUS TEETH." WHO Environmental Health: 106-109.

Braun, J. M. (2012). "Endocrine disrupting compounds, gonadal hormones, and autism." Developmental Medicine & Child Neurology 54[[1](#_ENREF_1)]: 1068.

Braun, J. M., et al. (2014). "Gestational exposure to endocrine-disrupting chemicals and reciprocal social, repetitive, and stereotypic behaviors in 4- and 5-year-old children: the HOME study." Environ Health Perspect 122(5): 513-520.

Braun, J. M., et al. (2013). "Phthalate exposure and children's health." Curr Opin Pediatr 25(2): 247-254.

Brent, J. (2013). "Commentary on the abuse of metal chelation therapy in patients with autism spectrum disorders." J Med Toxicol 9[[1](#_ENREF_1)]: 370-372.

Bronks, I. G. and R. E. Evans (1968). "A follow-up survey op psychiatric patients referred to industrial rehabilitation units." Rehab. (Lond. ) 65: 5-10.

Brown, A. E. and L. A. Sorbera (2013). "THERAPEUTIC TARGETS FOR DUCHENNE MUSCULAR DYSTROPHY (DMD)." Drugs of the Future 38(6): 395-406.

Brown Jr, J. S. (2007). "Psychiatric Issues in Toxic Exposures." Psychiatric Clinics of North America 30[[1](#_ENREF_1)]: 837-854.

Brubacher, R. L. (1972). "Ohio's project prep." Rkhab.Rec. 13[[1](#_ENREF_1)]: 24-26.

Brulotte, J., et al. (2009). "Complementary, holistic, and integrative medicine: Fish oils and neurodevelopmental disorders." Pediatrics in Review 30[[1](#_ENREF_1)]: e29-e33.

Brumback, R. A. (2007). "Note from editor-in-chief about erratum for Ip et al article." Journal of Child Neurology 22[[1](#_ENREF_1)]: 1321-1323.

Brumback, R. A. (2008). "The further mercurial adventures of Ip et al." Journal of Child Neurology 23(12): 1497.

Brumback, R. A. (2008). "A never ending saga: the mercury and autism articles by Ip et al." J Child Neurol 23(7): 725.

Buck Louis, G. M. and R. Sundaram (2012). "Exposome: Time for transformative research." Statistics in Medicine 31(22): 2569-2575.

Buiatti, M. (1998). "Mathematical modeling in biology: A critical assessment." Nuovo Cimento Della Societa Italiana Di Fisica D-Condensed Matter Atomic Molecular and Chemical Physics Fluids Plasmas Biophysics 20[[1](#_ENREF_1)]: 79-89.

Burns, J. G. and F. Mery (2010). "Transgenerational memory effect of ageing in Drosophila." J Evol Biol 23[[1](#_ENREF_1)]: 678-686.

Buxbaum, J. D. and P. R. Hof (2013). The Environment in Autism Spectrum Disorders. San Diego, Elsevier Academic Press Inc.

Calahorro, F. and M. Ruiz-Rubio (2013). "Human alpha- and beta-NRXN1 isoforms rescue behavioral impairments of Caenorhabditis elegans neurexin-deficient mutants." Genes, Brain and Behavior 12[[1](#_ENREF_1)]: 453-464.

Calderon-Garciduenas, L., et al. (2014). "Air pollution and detrimental effects on children's brain. The need for a multidisciplinary approach to the issue complexity and challenges." Frontiers in Human Neuroscience 8: 7.

Calton, M., et al. (2014). "Impaired Hypercarbic and Hypoxic Responses from Developmental Loss of Cerebellar Purkinje Neurons: Implications for Sudden Infant Death Syndrome." The Cerebellum.

Campbell, M. G., et al. (2013). "Pathway-based outlier method reveals heterogeneous genomic structure of autism in blood transcriptome." BMC Med Genomics 6: 34.

Camurri, A. and P. Ferrentino (1999). "Interactive environments for music and multimedia." Multimedia Systems 7[[1](#_ENREF_1)]: 32-47.

Cannell, J. J. (2008). "Autism and vitamin D." Med Hypotheses 70[[1](#_ENREF_1)]: 750-759.

Cannell, J. J. (2010). "On the aetiology of autism." Acta Paediatrica, International Journal of Paediatrics 99[[1](#_ENREF_1)]: 1128-1130.

Cannell, J. J. and W. B. Grant (2013). "What is the role of vitamin D in autism?" Dermatoendocrinol 5[[1](#_ENREF_1)]: 199-204.

Carneiro, A. M., et al. (2009). "Functional coding variation in recombinant inbred mouse lines reveals multiple serotonin transporter-associated phenotypes." Proc Natl Acad Sci U S A 106(6): 2047-2052.

Carneiro, M. F. H., et al. (2013). "Thimerosal in childhood vaccines contributes to accumulating mercury toxicity in the kidney." Toxicological and Environmental Chemistry 95[[1](#_ENREF_1)]: 1424-1447.

Carpenter, D. O. (2013). Intellectual Developmental Disability Syndromes and Organic Chemicals, Blackwell Science Publ, Osney Mead, Oxford Ox2 0el, Uk.

Carpenter, E. M., et al. (2009). "Reduced reelin expression and prenatal pesticide exposure alter CNS anatomy and mouse behavior in a possible model for autism." Society for Neuroscience Abstract Viewer and Itinerary Planner 39.

Carpenter, W., et al. (2012). "Nimh research domain criteria project: How will the criteria work for studies of diagnosis and new drug development?" Neuropsychopharmacology 38: S13-S14.

Carrasco, N. J. M. (2009). "Status of the State of Undernutrition and Environmental Influences for Disease in Later Generations." Explore: The Journal of Science and Healing 5[[1](#_ENREF_1)]: 55-58.

Carrozzo, M. (2011). "Urban legends: Facts and myths in oral diseases." Oral Diseases 17[[1](#_ENREF_1)]: 753-754.

Casanova, M. F. (2008). "The minicolumnopathy of autism: A link between migraine and gastrointestinal symptoms." Med Hypotheses 70[[1](#_ENREF_1)]: 73-80.

Cass, H., et al. (2006). "Medical investigation of children with autistic spectrum disorders." Child: Care, Health and Development 32(5): 521-533.

Cave, S. F. (2008). "The history of vaccinations in the light of the autism epidemic." Altern Ther Health Med 14(6): 54-57.

Ceylan, M., et al. (2010). "Oxidative imbalance in child and adolescent patients with attention-deficit/hyperactivity disorder." Progress in Neuro-Psychopharmacology & Biological Psychiatry 34[[1](#_ENREF_1)]: 1491-1494.

Cezar, G. G. (2006). "Embryonic stem cells: A new avenue to evaluate the effects of chemicals in humans." International Journal of Pharmaceutical Medicine 20(2): 107-114.

Chancellor, A. (2005). "How good at neurology are you?" Practical Neurology 5[[1](#_ENREF_1)]: 252-254.

Chang, S. W. C., et al. (2012). "Inhaled oxytocin amplifies both vicarious reinforcement and self reinforcement in rhesus macaques (Macaca mulatta)." Proceedings of the National Academy of Sciences of the United States of America 109[[2](#_ENREF_2)]: 959-964.

Chang, S. W. C. and M. L. Platt (2014). "Oxytocin and social cognition in rhesus macaques: Implications for understanding and treating human psychopathology." Brain Research 1580: 57-68.

Charlot, L., et al. (2011). "Non-psychiatric health problems among psychiatric inpatients with intellectual disabilities." Journal of Intellectual Disability Research 55(2): 199-209.

Chaste, P. and M. Leboyer (2012). "Autism risk factors: Genes, environment, and gene-environment interactions." Dialogues in Clinical Neuroscience 14[[2](#_ENREF_2)]: 281-292.

Chatterjee, A. and C. Okeefe (2010). "Current controversies in the USA regarding vaccine safety." Expert Review of Vaccines 9(5): 497-502.

Chaudhuri, S., et al. (2010). "Encapsulation of serotonin in beta-cyclodextrin nano-cavities: Fluorescence spectroscopic and molecular modeling studies." Journal of Molecular Structure 975(1-3): 160-165.

Chauhan, A., et al. (2008). "Increased oxidative damage and free radical generation in lymphoblasts from autism." Journal of Neurochemistry 106(Suppl. 1): 44.

Chauhan, A., et al. (2009). "Mitochondrial abnormalities in lymphoblasts from autism." Journal of Neurochemistry 109(Suppl. 1): 273.

Chauhan, A., et al. (2008). "Increased copper-mediated oxidation of membrane phosphatidylethanolamine in autism." American Journal of Biochemistry and Biotechnology 4(2): 95-100.

Chauhan, V., et al. (2010). "BISPHENOL A, ENDOSULFAN AND TERBUTALINE DECREASE MEMBRANE FLUIDITY: POTENTIAL ROLE IN AUTISM." Journal of Neurochemistry 115(Suppl. 1): 29.

Chen, H., et al. (2012). "Spondylodiscitis and epidural abscess after stem cell injections to the spine for chronic back pain: A Case Report." Pain Medicine (United States) 13(2): 335-336.

Chen, W. S., et al. (2012). "Functional expression of rat neuroligin-1 extracellular fragment by a bi-cistronic baculovirus expression vector." Protein Expression and Purification 81[[1](#_ENREF_1)]: 18-24.

Cheng, Y., et al. (2012). "Neurodevelopmental Consequences of Sub-Clinical Carbon Monoxide Exposure in Newborn Mice." Plos One 7(2): 12.

Cheslack-Postava, K., et al. (2013). "Maternal serum persistent organic pollutants in the Finnish Prenatal Study of Autism: A pilot study." Neurotoxicology and Teratology 38: 1-5.

Christophersen, O. A. (2012). "Should autism be considered a canary bird telling that Homo sapiens may be on its way to extinction?" Microb Ecol Health Dis 23.

Cicha, R., et al. (2009). Neurotoxicity, Autism, and Cognitive Impairment. Hauppauge, Nova Science Publishers, Inc.

Clark, B., et al. (2010). "Is lead a concern in Canadian autistic children?" Paediatrics and Child Health 15[[1](#_ENREF_1)]: 17-22.

Clarke, R., et al. (2010). "The magnetism of surgery: small bowel obstruction in an 8-year-old boy." BMJ Case Rep 2010.

Clarkson, T. W., et al. (2003). "Human Exposure to Mercury: The Three Modern Dilemmas." Journal of Trace Elements in Experimental Medicine 16[[1](#_ENREF_1)]: 321-343.

Claudio, L. (2003). "PILGrimage to New York." Environmental Health Perspectives 111(16): A879.

Clements, C. J. and P. B. McIntyre (2006). "When science is not enough - a risk/benefit profile of thiomersal-containing vaccines." Expert Opin Drug Saf 5[[1](#_ENREF_1)]: 17-29.

Clifton, J. C., 2nd (2007). "Mercury exposure and public health." Pediatr Clin North Am 54(2): 237-269, viii.

Coghill, D. (2011). "Pragmatic measures in paediatric psychopharmacology - Are we getting it right?" European Neuropsychopharmacology 21[[1](#_ENREF_1)]: 571-583.

Cohen, B. I. (2006). "Ammonia (NH3), nitric oxide (NO) and nitrous oxide (N2O)--the connection with infantile autism." Autism 10(2): 221-223.

Cohen, D. J., et al. (1976). "Pica and elevated blood lead level in autistic and atypical children." Am J Dis Child 130[[1](#_ENREF_1)]: 47-48.

Cohen, D. J., et al. (1982). "Blood lead in autistic children." Lancet 2(8289): 94-95.

Cohen, J. P., et al. (2013). "Plasma and urine dimercaptopropanesulfonate concentrations after dermal application of transdermal DMPS (TD-DMPS)." J Med Toxicol 9[[1](#_ENREF_1)]: 9-15.

Colborn, T. (2004). "Neurodevelopment and endocrine disruption." Environmental Health Perspectives 112[[1](#_ENREF_1)]: 944-949.

Coleman, C. I. and A. A. Perugini (2012). Case Studies. 78.

Colvin, S. M. and K. Y. Kwan (2014). "Dysregulated nitric oxide signaling as a candidate mechanism of fragile X syndrome and other neuropsychiatric disorders." Front Genet 5: 239.

Connor, C. M., et al. (2011). "White matter neuron alterations in schizophrenia and related disorders." Int J Dev Neurosci 29[[2](#_ENREF_2)]: 325-334.

Connors, S. L., et al. (2008). "Fetal mechanisms in neurodevelopmental disorders." Pediatric Neurology 38[[2](#_ENREF_2)]: 163-176.

Contini, C., et al. (2010). "Chlamydophila pneumoniae Infection and Its Role in Neurological Disorders." Interdiscip Perspect Infect Dis 2010: 273573.

Corbett, S. J. and C. C. S. Poon (2008). "Toxic levels of mercury in Chinese infants eating fish congee." Medical Journal of Australia 188[[1](#_ENREF_1)]: 59-60.

Cordero, J. F. (2003). "A New Look at Behavioral Outcomes and Teratogens: A Commentary." Birth Defects Research Part A - Clinical and Molecular Teratology 67[[1](#_ENREF_1)]: 900-902.

Cory-Slechta, D. A., et al. (2008). "The environmental etiologies of neurobehavioral deficits and disorders: Weaving complex outcomes and risk modifiers into the equation." NeuroToxicology 29(5): 759-760.

Cosgrove, L., et al. (2014). "Tripartite Conflicts of Interest and High Stakes Patent Extensions in the DSM-5." Psychotherapy and Psychosomatics 83(2): 106-113.

Costa, L. G., et al. (2014). "Neurotoxicants are in the air: convergence of human, animal, and in vitro studies on the effects of air pollution on the brain." Biomed Res Int 2014: 736385.

Costello, E. (2005). "Complementary and alternative therapies: Considerations for families after international adoption." Pediatric Clinics of North America 52(5): 1463-+.

Coury, D. L. and P. L. Nash (2003). "Epidemiology and etiology of autistic spectrum disorders: Difficult to determine." Pediatric Annals 32(10): 696-700.

Cowan, S. A. (2004). "Mercury in influenza vaccine." Ugeskrift for Laeger 166(38): 3289-3291.

Cozzolino, R., et al. (2014). "Use of solid-phase microextraction coupled to gas chromatography-mass spectrometry for determination of urinary volatile organic compounds in autistic children compared with healthy controls." Anal Bioanal Chem 406[[1](#_ENREF_1)]: 4649-4662.

Cranmer, J. M. (2008). "Twenty-fourth International Neurotoxicology Conference. "Environmental Etiologies of Neurological Disorders: Modifiers of Risk"." NeuroToxicology 29(5): 753-754.

Cranmer, J. M., et al. (2012). "Environmentally Triggered Neurodevelopmental Disorders - Focus on Endocrine Disruption and Sex Differences in Autism, ADHD, and Schizophrenia: Overview and Theme of the 27th International Neurotoxicology Conference." Neurotoxicology (Amsterdam) 33(6, Sp. Iss. SI).

Crespi, B. J. and D. L. Thiselton (2011). "Comparative immunogenetics of autism and schizophrenia." Genes Brain and Behavior 10(7): 689-701.

Crews, D. and J. A. McLachlan (2006). "Epigenetics, evolution, endocrine disruption, health, and disease." Endocrinology 147(6): S4-S10.

Cronin, M. (2006). "Currencies of exchange: Literature and the future of European language diversity." Futures 38(2): 146-157.

Cubala-Kucharska, M. (2010). "The review of most frequently occurring medical disorders related to aetiology of autism and the methods of treatment." Acta Neurobiol Exp (Wars) 70(2): 141-146.

Curran, M. P. (2011). "Aripiprazole: In the treatment of irritability associated with autistic disorder in pediatric patients." Pediatric Drugs 13[[2](#_ENREF_2)]: 197-204.

Currenti, S. A. (2010). "Understanding and determining the etiology of autism." Cell Mol Neurobiol 30(2): 161-171.

Curtis, J. T., et al. (2010). "Developmental exposure to metals alters subsequent social behavior in mature prairie voles: A refinement of a vole model of autism." Society for Neuroscience Abstract Viewer and Itinerary Planner 40.

Curtis, J. T., et al. (2010). "Chronic metals ingestion by prairie voles produces sex-specific deficits in social behavior: an animal model of autism." Behav Brain Res 213[[1](#_ENREF_1)]: 42-49.

Curtis, J. T., et al. (2009). "Metals ingestion by prairie voles produces sex-specific deficits in social behavior: An animal model of autism." Society for Neuroscience Abstract Viewer and Itinerary Planner 39.

Curtis, L. T. and K. Patel (2008). "Nutritional and environmental approaches to preventing and treating autism and attention deficit hyperactivity disorder (ADHD): a review." J Altern Complement Med 14[[1](#_ENREF_1)]: 79-85.

Daenen, E. W. P. M., et al. (2002). "The effects of neonatal lesions in the amygdala or ventral hippocampus on social behaviour later in life." Behavioural Brain Research 136(2): 571-582.

Daly, J. W. (2005). "Nicotinic agonists, antagonists, and modulators from natural sources." Cellular and Molecular Neurobiology 25(3-4): 513-552.

D'Amelio, M., et al. (2005). "Paraoxonase gene variants are associated with autism in North America, but not in Italy: possible regional specificity in gene-environment interactions." Mol Psychiatry 10[[1](#_ENREF_1)]: 1006-1016.

D'Amelio, M., et al. (2003). "Genetic study of paraoxonase gene variants in autistic disorder." American Journal of Human Genetics 73(5): 509.

Das, U. N. (2013). "Autism as a disorder of deficiency of brain-derived neurotrophic factor and altered metabolism of polyunsaturated fatty acids." Nutrition 29(10): 1175-1185.

Davidson, P. W., et al. (2004). "Mercury exposure and child development outcomes." Pediatrics 113(4 Suppl): 1023-1029.

Davis, D. A., et al. (2012). "Nano-particulates from urban vehicular exhaust decrease glutamate receptor function." Society for Neuroscience Abstract Viewer and Itinerary Planner 42.

Davis, D. A., et al. (2013). "Prenatal Exposure to Urban Air Nanoparticles in Mice Causes Altered Neuronal Differentiation and Depression-Like Responses." Plos One 8(5): 7.

Davis, R. L. (2012). "Neurobiological marker in ASD." International Journal of Neuropsychopharmacology 15: 21-22.

Davis, R. L., et al. (2010). "Heavy metals and neuroimmune modulation: Importance in autism?" Society for Neuroscience Abstract Viewer and Itinerary Planner 40.

Davis, R. L., et al. (2009). "Heavy metal toxicity and chemokine expression in the brain: Importance in autism?" Society for Neuroscience Abstract Viewer and Itinerary Planner 39.

Davis, S. R., et al. (2014). "Respiratory medication use in an Australian developmental disability clinic population: Messages for health care professionals." Australian Journal of Primary Health 20[[2](#_ENREF_2)]: 278-284.

Davis, T. N., et al. (2013). "Chelation treatment for autism spectrum disorders: A systematic review." Research in Autism Spectrum Disorders 7[[1](#_ENREF_1)]: 49-55.

Dawson, G. (2013). "Dramatic increase in autism prevalence parallels explosion of research into its biology and causes." JAMA Psychiatry 70[[1](#_ENREF_1)]: 9-10.

Dayya, D. (2006). "Dataset Analysis and Management for Use in an Ecological Analysis on Exposures to Heavy Metal Contaminated Air and Disease Rates of Autism in the United States." Retrieved 11/13/2014, from http://digitalcommons.uconn.edu/uchcgs_masters/34.

De Angelis, M., et al. (2013). "Fecal microbiota and metabolome of children with autism and pervasive developmental disorder not otherwise specified." PLoS One 8(10): e76993.

de Cock, M., et al. (2012). "Does perinatal exposure to endocrine disruptors induce autism spectrum and attention deficit hyperactivity disorders? Review." Acta Paediatr 101[[1](#_ENREF_1)]: 811-818.

de Cock, M. and M. Van de Bor (2010). "AUTISM SPECTRUM (ASD) AND ATTENTION DEFICIT HYPERACTIVITY (ADHD) DISORDERS EPIDEMICS: DOES FETAL EXPOSURE TO ENDOCRINE DISRUPTING CHEMICALS PLAY A ROLE?" Pediatric Research 68(Suppl. 1): 122.

De Felice, C., et al. (2009). "Systemic oxidative stress in classic Rett syndrome." Free Radic Biol Med 47[[1](#_ENREF_1)]: 440-448.

De Gavre, P. (2006). "Savage industrial marketing, exploitation of the sufferings of the autistics The Inserm has gone out of the red box!" ANAE - Approche Neuropsychologique des Apprentissages chez l'Enfant 18(2): 72+125.

De Groot, A. S., et al. (2010). "Species neutral correlates of immunogenicity for vaccines and protein therapeutics: Fact or science fiction." Human Vaccines 6(5): 371-372.

De Jaco, A., et al. (2010). "Folding anomalies of neuroligin3 caused by a mutation in the alpha/beta-hydrolase fold domain." Chem Biol Interact 187(1-3): 56-58.

De Noni, I. (2008). "Release of beta-casomorphins 5 and 7 during simulated gastro-intestinal digestion of bovine beta-casein variants and milk-based infant formulas." Food Chemistry 110[[1](#_ENREF_1)]: 897-903.

De Palma, G., et al. (2012). "Lack of correlation between metallic elements analyzed in hair by ICP-MS and autism." J Autism Dev Disord 42[[2](#_ENREF_2)]: 342-353.

De Vries, T. W., et al. (2008). "Inhaled corticosteroids do not affect behaviour." Acta Paediatrica, International Journal of Paediatrics 97(6): 786-789.

deCastro, B. R. (2014). "Acrolein and Asthma Attack Prevalence in a Representative Sample of the United States Adult Population 2000-2009." Plos One 9(5): 10.

DeLorey, T. M., et al. (2008). "Gabrb3 gene deficient mice exhibit impaired social and exploratory behaviors, deficits in non-selective attention and hypoplasia of cerebellar vermal lobules: A potential model of autism spectrum disorder." Behavioural Brain Research 187(2): 207-220.

Delorme, R., et al. (2010). "Mutation screening of NOS1AP gene in a large sample of psychiatric patients and controls." BMC Med Genet 11: 108.

DeLuca, G. C., et al. (2013). "Review: The role of vitamin D in nervous system health and disease." Neuropathology and Applied Neurobiology 39(5): 458-484.

Denholm, E. M. and W. J. Martin, II (2008). "Translational research in environmental health sciences." Translational Research 151(2): 57-58.

Deoni, S. C. and M. Catani (2007). "Visualization of the deep cerebellar nuclei using quantitative T1 and rho magnetic resonance imaging at 3 Tesla." Neuroimage 37[[1](#_ENREF_1)]: 1260-1266.

Desai, M., et al. (2013). "Bisphenol a increases neural progenitor cell proliferation and alters neurogenesis." Reproductive Sciences 20[[2](#_ENREF_2)]: 204A.

DeSoto, C. M. and T. R. Hitlan (2008). "Concerning blood mercury levels and autism: A need to clarify [2]." Journal of Child Neurology 23[[1](#_ENREF_1)]: 463-465.

DeSoto, M. C. (2008). "A reply to Soden et al.: your data shows autistic children have higher levels of heavy metals." Clin Toxicol (Phila) 46(10): 1098; author reply 1098.

DeSoto, M. C. (2009). "Ockham's Razor and autism: The case for developmental neurotoxins contributing to a disease of neurodevelopment." Neurotoxicology 30[[2](#_ENREF_2)]: 331-337.

Desoto, M. C. and R. T. Hitlan (2007). "Blood levels of mercury are related to diagnosis of autism: a reanalysis of an important data set." J Child Neurol 22[[1](#_ENREF_1)]: 1308-1311.

Desoto, M. C. and R. T. Hitlan (2010). "Sorting out the spinning of autism: heavy metals and the question of incidence." Acta Neurobiol Exp (Wars) 70(2): 165-176.

Dessoki, H., et al. (2012). "TOXIC METALS AND ESSENTIAL MINERALS AND SEVERITY OF SYMPTOMS AMONG CHILDREN WITH AUTISM." European Psychiatry 27: 1.

Deth, R., et al. (2008). "How environmental and genetic factors combine to cause autism: A redox/methylation hypothesis." Neurotoxicology 29[[1](#_ENREF_1)]: 190-201.

Deth, R. C. (2013). "Autism: A redox/methylation disorder." Global Advances In Health and Medicine 2(6): 68-73.

Di Nardo, A., et al. (2009). "Tuberous Sclerosis Complex Activity Is Required to Control Neuronal Stress Responses in an mTOR-Dependent Manner." Journal of Neuroscience 29(18): 5926-5937.

Dietert, R. R. and J. M. Dietert (2008). "Possible role for early-life immune insult including developmental immunotoxicity in chronic fatigue syndrome (CFS) or myalgic encephalomyelitis (ME)." Toxicology 247[[1](#_ENREF_1)]: 61-72.

Dietert, R. R. and J. M. Dietert (2008). "Potential for early-life immune insult including developmental immunotoxicity in autism and autism spectrum disorders: Focus on critical windows of immune vulnerability." Journal of Toxicology and Environmental Health-Part B-Critical Reviews 11[[1](#_ENREF_1)]: 660-680.

Dietert, R. R., et al. (2011). "Environmental risk factors for autism." Emerging Health Threats Journal 4[[1](#_ENREF_1)].

Dietrich, K. N., et al. (2005). "Principles and practices of neurodevelopmental assessment in children: Lessons learned from the Centers for Children's Environmental Health and Disease Prevention Research." Environmental Health Perspectives 113(10): 1437-1446.

Dikme, G., et al. (2011). "The relationship between heavy metal exposure and chronic neurological diseases in children." Acta Paediatrica, International Journal of Paediatrics 100: 26-27.

Dikme, G., et al. (2013). "The relation between blood lead and mercury levels and chronic neurological diseases in children." Turk Pediatri Arsivi 48[[2](#_ENREF_2)]: 221-225.

DiMaggio, C., et al. "Early childhood exposure to anesthesia and risk of developmental and behavioral disorders in a sibling birth cohort." Anesth Analg.

Dochniak, M. J. (2007). "Autism spectrum disorders - Exogenous protein insult." Med Hypotheses 69[[2](#_ENREF_2)]: 545-549.

Doherty, J. D. (2006). "Screening pesticides for neuropathogenicity." Journal of Biomedicine and Biotechnology 2006.

Donders, J. (2010). "Evidence-based neuropsychological assessment and intervention." Developmental Medicine and Child Neurology 52: 1.

Dooley, E. E. (2013). "The beat." Environmental Health Perspectives 121[[1](#_ENREF_1)]: A17.

Dooley, J. M. (2006). "Tic Disorders in Childhood." Seminars in Pediatric Neurology 13[[1](#_ENREF_1)]: 231-242.

Dorea, J. G. (2010). "Making sense of epidemiological studies of young children exposed to thimerosal in vaccines." Clinica Chimica Acta 411(21-22): 1580-1586.

Dorea, J. G. (2010). "More on low-level non-occupational mercury exposure and health concerns." Science of the Total Environment 408[[1](#_ENREF_1)]: 2008-2009.

Dosman, C. F., et al. (2007). "Children with autism: effect of iron supplementation on sleep and ferritin." Pediatr Neurol 36[[2](#_ENREF_2)]: 152-158.

Dosman, C. F., et al. (2006). "Ferritin as an indicator of suspected iron deficiency in children with autism spectrum disorder: prevalence of low serum ferritin concentration." Dev Med Child Neurol 48(12): 1008-1009.

Downing, D. (2000). "Mercury again." Journal of Nutritional and Environmental Medicine 10[[1](#_ENREF_1)]: 267-269.

Downing, D. (2007). "Autism: Are we entering the final straight? In memory of Bernard Rimland." Journal of Nutritional and Environmental Medicine 16(3-4): 173-180.

Driessen, T., et al. (2013). "Postpartum gene expression changes in the medial preoptic area exhibit a high concordance with autism and other mental health disorders." Society for Neuroscience Abstract Viewer and Itinerary Planner 43.

Driessen, T. M., et al. (2014). "Genes showing altered expression in the medial preoptic area in the highly social maternal phenotype are related to autism and other disorders with social deficits." Bmc Neuroscience 15: 16.

Drum, D. A. (2009). "Are toxic biometals destroying your children's future?" Biometals 22(5): 697-700.

Dubovicky, M. (2010). "Neurobehavioral manifestations of developmental impairment of the brain." Interdisciplinary Toxicology 3(2): 59-67.

Dubovicky, M., et al. (2008). "Evaluation of developmental neurotoxicity: some important issues focused on neurobehavioral development." Interdiscip Toxicol 1(3-4): 206-210.

Dufault, R., et al. (2012). "A macroepigenetic approach to identify factors responsible for the autism epidemic in the United States." Clin Epigenetics 4[[1](#_ENREF_1)]: 6.

Dunaway, K., et al. (2013). "A Bioinformatic Approach to Determine the Effect of Organic Pollutants on the Genome and Epigenome." Environmental and Molecular Mutagenesis 54(Suppl. 1): S24.

Duncan, P. M., et al. (2008). "Bright futures: The screening table recommendations." Pediatric Annals 37[[2](#_ENREF_2)]: 152-158.

Dunn, A. G., et al. (2013). "The effects of industry sponsorship on comparator selection in trial registrations for neuropsychiatric conditions in children." PLoS One 8(12): e84951.

Duvall, M. G., et al. (2013). "Pulmonary Hypertension Associated With Scurvy and Vitamin Deficiencies in an Autistic Child." Pediatrics 132(6): E1699-E1703.

Ecker, C., et al. (2013). "Developing new pharmacotherapies for autism." J Intern Med 274[[1](#_ENREF_1)]: 308-320.

Edelson, S. B. and D. S. Cantor (1998). "Autism: Xenobiotic influences." Toxicology and Industrial Health 14[[1](#_ENREF_1)]: 553-563.

Edelstein, L., et al. (2012). "Neuronal nitric oxide synthase immunoreactivity in the human claustrum: A light- and electron-microscopic investigation." Society for Neuroscience Abstract Viewer and Itinerary Planner 42.

Edwards, M. J. (2006). "Review: Hyperthermia and fever during pregnancy." Birth Defects Research Part A - Clinical and Molecular Teratology 76(7): 507-516.

Egiebor, E., et al. (2013). "The kinetic signature of toxicity of four heavy metals and their mixtures on MCF7 breast cancer cell line." Int J Environ Res Public Health 10(10): 5209-5220.

El Sadek, M. M., et al. (2013). "Synthesis and bioassay of a new class of furanyl-1,3,4-oxadiazole derivatives." Molecules 18(7): 8550-8562.

El-Ansary, A., et al. (2010). "Measurement of selected ions related to oxidative stress and energy metabolism in Saudi autistic children." Clinical Biochemistry 43(1-2): 63-70.

El-Ansary, A. K., et al. (2011). "Relationship between chronic lead toxicity and plasma neurotransmitters in autistic patients from Saudi Arabia." Clin Biochem 44(13): 1116-1120.

El-Ansary, A. K., et al. (2011). "Plasma fatty acids as diagnostic markers in autistic patients from Saudi Arabia." Lipids in Health and Disease 10.

El-baz, F., et al. (2010). "Hair mercury measurement in Egyptian autistic children." Egyptian Journal of Medical Human Genetics 11(2): 135-141.

Elliman, D. and N. Sengupta (2005). "Measles." Curr Opin Infect Dis 18[[2](#_ENREF_2)]: 229-234.

Ellis, C. N., et al. (2012). "Understanding and Managing Atopic Dermatitis in Adult Patients." Seminars in Cutaneous Medicine and Surgery 31(3 SUPPL.): S18-S22.

Ellsworth, H., et al. (2012). "Compounding error leads to clonidine toxicity associated with hyperglycemia." Clinical Toxicology 50(7): 606.

Elphinstone, P. "MMR and autism: the debate continues." Lancet.

Eppright, T. D., et al. (1996). "Attention deficit hyperactivity disorder, infantile autism, and elevated blood-lead: a possible relationship." Mo Med 93[[2](#_ENREF_2)]: 136-138.

Eskenazi, B., et al. (2010). "PON1 and neurodevelopment in children from the CHAMACOS study exposed to organophosphate pesticides in utero." Environ Health Perspect 118(12): 1775-1781.

Eskenazi, B. and P. J. Landrigan (2002). "Environmental Health Perspectives and children's environmental health." Environmental Health Perspectives 110(10): A559-A560.

Eskenazi, B., et al. (2007). "Organophosphate pesticide exposure and neurodevelopment in young Mexican-American children." Environ Health Perspect 115(5): 792-798.

Eskenazi, B., et al. (2008). "Pesticide toxicity and the developing brain." Basic Clin Pharmacol Toxicol 102(2): 228-236.

Essa, M. M., et al. (2013). "Impaired antioxidant status and reduced energy metabolism in autistic children." Research in Autism Spectrum Disorders 7(5): 557-565.

Essa, M. M., et al. (2012). "Increased markers of oxidative stress in autistic children of the Sultanate of Oman." Biol Trace Elem Res 147(1-3): 25-27.

Evans, M. (2005). "Flu shot." Canadian Family Physician 51(NOV.): 1511-1516.

Evans, T. A., et al. (2008). "The autistic phenotype exhibits a remarkably localized modification of brain protein by products of free radical-induced lipid oxidation." American Journal of Biochemistry and Biotechnology 4(2): 61-72.

Ewing, G. E. (2009). "What is regressive autism and why does it occur? Is it the consequence of multi-systemic dysfunction affecting the elimination of heavy metals and the ability to regulate neural temperature?" N Am J Med Sci 1(2): 28-47.

Faber, S., et al. (2009). "The plasma zinc/serum copper ratio as a biomarker in children with autism spectrum disorders." Biomarkers 14[[2](#_ENREF_2)]: 171-180.

Fakira, A. K., et al. (2012). "Purkinje cell dysfunction and delayed death in plasma membrane calcium ATPase 2-heterozygous mice." Mol Cell Neurosci 51(1-2): 22-31.

Fanjiang, G. and R. E. Kleinman (2007). "Nutrition and performance in children." Current Opinion in Clinical Nutrition and Metabolic Care 10[[2](#_ENREF_2)]: 342-347.

Farooq, M. U., et al. (2009). "Levetiracetam for managing neurologic and psychiatric disorders." American Journal of Health-System Pharmacy 66(6): 541-561.

Fatemi, S., et al. (2010). "Prenatal infection at E7 leads to altered gene expression in placenta and in brains of exposed offspring: A DNA microarray study." Society for Neuroscience Abstract Viewer and Itinerary Planner 40.

Fatemi, S. H., et al. (2012). "Consensus paper: Pathological role of the cerebellum in Autism." Cerebellum 11[[2](#_ENREF_2)]: 777-807.

Fatemi, S. H., et al. (2000). "Prenatal viral infection causes alterations in nNOS expression in developing mouse brains." Neuroreport 11(7): 1493-1496.

Fatemi, S. H., et al. (2009). "Abnormal expression of myelination genes and alterations in white matter fractional anisotropy following prenatal viral influenza infection at E16 in mice." Schizophrenia Research 112(1-3): 46-53.

Fedor-Freybergh, P. G. (2007). "Some words from the editor-in-chief of the Neuroendocrinology Letters." Neuroendocrinology Letters 28[[1](#_ENREF_1)]: 333-334.

Fernell, E., et al. (2007). "No evidence for a clear link between active intestinal inflammation and autism based on analyses of faecal calprotectin and rectal nitric oxide." Acta Paediatr 96(7): 1076-1079.

Fido, A. and S. Al-Saad (2005). "Toxic trace elements in the hair of children with autism." Autism 9[[2](#_ENREF_2)]: 290-298.

Figgitt, D. P. and K. J. McClellan (2000). "Fluvoxamine: An updated review of its use in the management of adults with anxiety disorders." Drugs 60[[1](#_ENREF_1)]: 925-954.

Filipek, P. A., et al. (2000). "Practice parameter: Screening and diagnosis of autism. Report of the quality standards subcommittee of the American Academy of Neurology and the Child Neurology Society." Neurology 55[[1](#_ENREF_1)]: 468-479.

Fitzpatrick, M. (2003). "Heavy metal." Lancet 361(9369): 1664.

Fitzpatrick, M. (2007). "Autism and environmental toxicity." Lancet Neurology 6[[1](#_ENREF_1)]: 297.

Fitzpatrick, M. (2007). "The end of the road for the campaign against MMR." British Journal of General Practice 57(541): 679.

Flechas, J. (2013). "Jorge Flechas, MD: the potential of oxytocin, nitric oxide, and iodine." Altern Ther Health Med 19[[1](#_ENREF_1)]: 50-56.

France-Stiglic, A., et al. (2011). "Determination of urinary porphyrines in families with autistic members." European Journal of Pharmaceutical Sciences 44: 51-52.

Francois, G., et al. (2005). "Vaccine safety controversies and the future of vaccination programs." Pediatr Infect Dis J 24[[1](#_ENREF_1)]: 953-961.

Franke, B., et al. (2012). "The genetics of attention deficit/hyperactivity disorder in adults, a review." Molecular Psychiatry 17(10): 960-987.

Franke, B., et al. (2009). "Genome-wide association studies in ADHD." Human Genetics 126[[1](#_ENREF_1)]: 13-50.

Franz, K. J. (2013). "Clawing back: Broadening the notion of metal chelators in medicine." Current Opinion in Chemical Biology 17(2): 143-149.

Frick, L. R., et al. (2013). "Microglial Dysregulation in Psychiatric Disease." Clinical & Developmental Immunology: 10.

Frustaci, A., et al. (2012). "Oxidative stress-related biomarkers in autism: systematic review and meta-analyses." Free Radic Biol Med 52(10): 2128-2141.

Frye, R. E., et al. (2013). "Metabolic effects of sapropterin treatment in autism spectrum disorder: a preliminary study." Transl Psychiatry 3: e237.

Frye, R. E., et al. (2010). "Tetrahydrobiopterin as a Novel Therapeutic Intervention for Autism." Neurotherapeutics 7[[2](#_ENREF_2)]: 241-249.

Frye, R. E. and S. J. James (2014). "Metabolic pathology of autism in relation to redox metabolism." Biomarkers in Medicine 8[[2](#_ENREF_2)]: 321-330.

Fudenberg, H. H. (2003). "A micro-epidemic of autism and myalgic encephalitis in Dublin suburbs; Search for the underlyng cause." IJCI - International Journal of Clinical Investigation 11(2-4): 23-26.

Fujikawa-Brooks, S., et al. (2010). "The effect of rate stress on the auditory brainstem response in autism: A preliminary report." International Journal of Audiology 49(2): 129-140.

Fujiwara, M., et al. (2013). "Effects of the chemical chaperone 4-phenylbutylate on the function of the serotonin transporter [[3](#_ENREF_3)] expressed in COS-7 cells." Journal of Pharmacological Sciences 122(2): 71-83.

Furlano, R. I., et al. (2014). "Bone Fractures in Children with Autistic Spectrum Disorder." Journal of Developmental and Behavioral Pediatrics 35(6): 353-359.

Furlong, M. A., et al. (2014). "Prenatal exposure to organophosphate pesticides and reciprocal social behavior in childhood." Environ Int 70: 125-131.

Garibovic, E., et al. (2011). "Environmental factors in the pathogenesis of autism spectrum disorders." European Child and Adolescent Psychiatry 20: S142.

Garofalo, L. and A. Milano (2013). "Idiopathic calcinosis cutis universalis." European Journal of Pediatric Dermatology 23[[2](#_ENREF_2)]: 188-189.

Garreau, B., et al. (1994). "Effects of auditory stimulation on regional cerebral blood flow in autistic children." Developmental Brain Dysfunction 7(2-3): 119-128.

Geier, D. A., et al. (2010). "Blood mercury levels in autism spectrum disorder: Is there a threshold level?" Acta Neurobiol Exp (Wars) 70(2): 177-186.

Geier, D. A., et al. (2011). "A significant relationship between mercury exposure from dental amalgams and urinary porphyrins: a further assessment of the Casa Pia children's dental amalgam trial." Biometals 24(2): 215-224.

Geier, D. A. and M. R. Geier (2003). "An assessment of the impact of thimerosal on childhood neurodevelopmental disorders." Pediatric Rehabilitation 6(2): 97-102.

Geier, D. A. and M. R. Geier (2004). "A comparative evaluation of the effects of MMR immunization and mercury doses from thimerosal-containing childhood vaccines on the population prevalence of autism." Med Sci Monit 10[[2](#_ENREF_2)]: Pi33-39.

Geier, D. A. and M. R. Geier (2006). "A clinical trial of combined anti-androgen and anti-heavy metal therapy in autistic disorders." Neuro Endocrinol Lett 27(6): 833-838.

Geier, D. A. and M. R. Geier (2006). "A prospective assessment of porphyrins in autistic disorders: a potential marker for heavy metal exposure." Neurotox Res 10[[1](#_ENREF_1)]: 57-64.

Geier, D. A. and M. R. Geier (2007). "A case series of children with apparent mercury toxic encephalopathies manifesting with clinical symptoms of regressive autistic disorders." Journal of Toxicology and Environmental Health - Part A: Current Issues 70(10): 837-851.

Geier, D. A. and M. R. Geier (2007). "A prospective study of mercury toxicity biomarkers in autistic spectrum disorders." J Toxicol Environ Health A 70(20): 1723-1730.

Geier, D. A. and M. R. Geier (2007). "A prospective study of thimerosal-containing Rho(D)-immune globulin administration as a risk factor for autistic disorders." Journal of Maternal-Fetal and Neonatal Medicine 20(5): 385-390.

Geier, D. A., et al. (2009). "Reply to Martin's comments." Journal of the Neurological Sciences 280(1-2): 128-129.

Geier, D. A., et al. (2009). "A Prospective Study of Transsulfuration Biomarkers in Autistic Disorders." Neurochemical Research 34(2): 386-393.

Geier, D. A., et al. (2009). "Biomarkers of environmental toxicity and susceptibility in autism." J Neurol Sci 280(1-2): 101-108.

Geier, D. A., et al. (2009). "A prospective blinded evaluation of urinary porphyrins verses the clinical severity of autism spectrum disorders." J Toxicol Environ Health A 72(24): 1585-1591.

Geier, D. A., et al. (2010). "The biological basis of autism spectrum disorders: Understanding causation and treatment by clinical geneticists." Acta Neurobiol Exp (Wars) 70(2): 209-226.

Geier, D. A., et al. (2012). "Hair toxic metal concentrations and autism spectrum disorder severity in young children." Int J Environ Res Public Health 9(12): 4486-4497.

Geier, D. A., et al. (2009). "Mitochondrial dysfunction, impaired oxidative-reduction activity, degeneration, and death in human neuronal and fetal cells induced by low-level exposure to thimerosal and other metal compounds." Toxicol Environ Chem 91(3-4): 735-749.

Geier, D. A., et al. (2008). "A comprehensive review of mercury provoked autism." Indian J Med Res 128[[1](#_ENREF_1)]: 383-411.

Geier, D. A., et al. (2012). "A quantitative evaluation of brain dysfunction and body-burden of toxic metals." Medical Science Monitor 18(7): CR425-CR431.

Geier, M. R. and D. A. Geier (2005). "The potential importance of steroids in the treatment of autistic spectrum disorders and other disorders involving mercury toxicity." Med Hypotheses 64(5): 946-954.

Geller, R. J. (2013). "Pediatric antimony toxicity treated with succimer." Clinical Toxicology 51(7): 640.

Genuis, S. J. (2006). "The chemical erosion of human health: Adverse environmental exposure and in-utero pollution - Determinants of congenital disorders and chronic disease." Journal of Perinatal Medicine 34[[2](#_ENREF_2)]: 185-195.

George, G. N., et al. (2004). "Mercury binding to the chelation therapy agents DMSA and DMPS and the rational design of custom chelators for mercury." Chemical Research in Toxicology 17[[1](#_ENREF_1)]: 999-1006.

George, M., et al. (2010). "Encephalopathy from lead poisoning masquerading as a flu-like syndrome in an autistic child." Pediatric Emergency Care 26(5): 370-373.

George, M. S., et al. (1992). "CEREBRAL BLOOD FLOW ABNORMALITIES IN ADULTS WITH INFANTILE AUTISM." Journal of Nervous and Mental Disease 180(7): 413-417.

Ghanbari, Y., et al. (2014). "Identifying group discriminative and age regressive sub-networks from DTI-based connectivity via a unified framework of non-negative matrix factorization and graph embedding." Med Image Anal 18[[1](#_ENREF_1)]: 1337-1348.

Ghanizadeh, A. (2011). "Gold implants and increased expression of metallothionein-I/II as a novel hypothesized therapeutic approach for autism." Toxicology 283[[1](#_ENREF_1)]: 63-64.

Ghanizadeh, A. (2011). "Novel treatment for lead exposure in children with autism." Biol Trace Elem Res 142[[2](#_ENREF_2)]: 257-258.

Ghanizadeh, A. (2011). "Oxidative stress may mediate association of stereotypy and immunity in autism, a novel explanation with clinical and research implications." Journal of Neuroimmunology 232(1-2): 194-195.

Ghanizadeh, A. (2012). "Hydrogen as a novel hypothesized emerging treatment for oxidative stress in autism." European Review for Medical and Pharmacological Sciences 16[[1](#_ENREF_1)]: 1313-1314.

Ghanizadeh, A. and E. Moghimi-Sarani (2013). "A randomized double blind placebo controlled clinical trial of N-Acetylcysteine added to risperidone for treating autistic disorders." BMC Psychiatry 13: 196.

Ghosh, A., et al. (2013). "Drug discovery for autism spectrum disorder: challenges and opportunities." Nat Rev Drug Discov 12(10): 777-790.

Giacoia, G. P. and D. R. Mattison (2005). "Newborns and drug studies: The NICHD/FDA newborn drug development initiative." Clinical Therapeutics 27(6): 796-813.

Gil, M., et al. (2013). "Sexual experience increases oxytocin receptor gene expression and protein in the medial preoptic area of the male rat." Psychoneuroendocrinology 38[[1](#_ENREF_1)]: 1688-1697.

Gillie, O. (2012). "The Scots' paradox: Can sun exposure, or lack of it, explain major paradoxes in epidemiology?" Anticancer Research 32(1 PART 2): 237-248.

Giovedi, S., et al. (2014). "Involvement of synaptic genes in the pathogenesis of autism spectrum disorders: the case of synapsins." Front Pediatr 2: 94.

Giraldo, S. E. (2008). "Prenatal pesticide exposure and development of autism spectrum disorders in the California Central Valley." International Pediatrics 23(2): 98.

Goines, P. E. and P. Ashwood (2013). "Cytokine dysregulation in autism spectrum disorders (ASD): Possible role of the environment." Neurotoxicology and Teratology 36: 67-81.

Goldman, L. R. and S. Koduru (2000). "Chemicals in the environment and developmental toxicity to children: a public health and policy perspective." Environ Health Perspect 108 Suppl 3: 443-448.

Goldman, M. (2013). "New Frontiers for Collaborative Research." Science Translational Medicine 5(216): 216ed222.

Goldson, E. (2009). Autism: An Update. 56: 187-201.

Gombash, S. E., et al. (2014). "Intravenous AAV9 efficiently transduces myenteric neurons in neonate and juvenile mice." Front Mol Neurosci 7: 81.

Gondalia, S. V., et al. (2010). "Gastrointestinal microbiology in autistic spectrum disorder: a review." Reviews in Medical Microbiology 21[[2](#_ENREF_2)]: 44-50.

Gonzanulllez-Alzaga, B., et al. (2014). "A systematic review of neurodevelopmental effects of prenatal and postnatal organophosphate pesticide exposure." Toxicology Letters 230(2): 104-121.

Good, P. (2006). "Low-dose naltrexone for multiple sclerosis and autism: Does its benefit reveal a common cause?" Medical Hypotheses 67[[2](#_ENREF_2)]: 671-672.

Gordon, I. (1996). "Cerebral blood flow imaging in paediatrics: a review." Nucl Med Commun 17(12): 1021-1029.

Goswami, B., et al. (2009). "Paraoxonase: A multifaceted biomolecule." Clinica Chimica Acta 410(1-2): 1-12.

Goursaud, A. P. S. and J. Bachevalier (2007). "Social attachment in juvenile monkeys with neonatal lesion of the hippocampus, amygdala and orbital frontal cortex." Behavioural Brain Research 176[[1](#_ENREF_1)]: 75-93.

Grabrucker, A. M. (2013). "Environmental factors in autism." Frontiers in Psychiatry 3(JAN).

Grabrucker, A. M. (2014). "A role for synaptic zinc in ProSAP/Shank PSD scaffold malformation in autism spectrum disorders." Dev Neurobiol 74(2): 136-146.

Grabrucker, S., et al. (2014). "Zinc deficiency dysregulates the synaptic ProSAP/Shank scaffold and might contribute to autism spectrum disorders." Brain 137(Pt 1): 137-152.

Grandin, T. (2009). "How does visual thinking work in the mind of a person with autism? A personal account." Philos Trans R Soc Lond B Biol Sci 364(1522): 1437-1442.

Grandjean, P. and P. J. Landrigan (2006). "Developmental neurotoxicity of industrial chemicals." Lancet 368(9553): 2167-2178.

Grandjean, P. and P. J. Landrigan (2014). "Neurobehavioural effects of developmental toxicity." Lancet Neurol 13[[2](#_ENREF_2)]: 330-338.

Grether, J., et al. (2004). "Baby hair, mercury toxicity and autism." Int J Toxicol 23[[1](#_ENREF_1)]: 275-276.

Gross, L. (2009). "A broken trust: Lessons from the vaccine-autism wars." PLoS Biology 7(5).

Grys, W., et al. (2013). "Gas Chromatography-electron Capture Detection Method for Determination of Polyols in the Urine." Current Organic Chemistry 17(20): 2359-2364.

Guastella, A., et al. (2010). "Oxytocin as a treatment to improve social functioning in schizophrenia." Australian and New Zealand Journal of Psychiatry 44: A10.

Guastella, A. J. (2012). "A randomized controlled trial of oxytocin nasal spray to treat youth diagnosed with autism spectrum disorders." Biological Psychiatry 71[[1](#_ENREF_1)]: 234S.

Guastella, A. J., et al. (2013). "Recommendations for the standardisation of oxytocin nasal administration and guidelines for its reporting in human research." Psychoneuroendocrinology 38(5): 612-625.

Guida, N., et al. (2014). "Histone deacetylase 4 promotes ubiquitin-dependent proteasomal degradation of Sp3 in SH-SY5Y cells treated with di(2-ethylhexyl) phthalate (DEHP), determining neuronal death." Toxicology and Applied Pharmacology 280[[1](#_ENREF_1)]: 190-198.

Gustafsson, L. (2003). Neural network theory and recent neuroanatomical findings indicate that inadequate nitric oxide synthase will cause autism. Knowledge-Based Intellignet Information and Engineering Systems, Pt 2, Proceedings. V. Palade, R. J. Howlett and L. Jain. Berlin, Springer-Verlag Berlin. 2774: 1109-1114.

Gustafsson, L. (2004). "Comment on "Disruption in the inhibitory architecture of the cell minicolumn: implications for autism"." Neuroscientist 10[[2](#_ENREF_2)]: 189-191.

Guxens, M. and J. Sunyer (2012). "A review of epidemiological studies on neuropsychological effects of air pollution." Swiss Medical Weekly 141: 9.

Guzzi, G. (2007). "Of vaccine-autism, thimerosal, and metallothioneins [[1](#_ENREF_1)]." Pediatric Allergy and Immunology 18[[1](#_ENREF_1)]: 88.

Guzzi, G., et al. (2002). "Should amalgam fillings be removed? [[1](#_ENREF_1)] (multiple letters)." Lancet 360(9350): 2081.

Halladay, A. K., et al. (2009). "Animal models of autism spectrum disorders: Information for neurotoxicologists." NeuroToxicology 30(5): 811-821.

Hamade, A., et al. (2013). "Autism in children and correlates in Lebanon: a pilot case-control study." J Res Health Sci 13(2): 119-124.

Hartzell, S. and S. Seneff (2012). "Impaired Sulfate Metabolism and Epigenetics: Is There a Link in Autism?" Entropy 14(10): 1953-1977.

Hass, U. (2006). "The need for developmental neurotoxicity studies in risk assessment for developmental toxicity." Reproductive Toxicology 22(2): 148-156.

Hassanzadeh, L., et al. (2013). "Synthesis, radiolabeling and bioevaluation of a novel arylpiperazine derivative containing triazole as a 5-HT1A receptor imaging agents." Nuclear Medicine and Biology 40(2): 227-232.

Haug, F. (1974). "HUMAN FACTOR - CONTRIBUTIONS TO INDUSTRIAL PSYCHOLOGY AND PEDAGOGY - GERMAN - ASPERGER,KH." Argument 16(3-4): 321-322.

Heberling, C. A., et al. (2013). "Hypothesis for a systems connectivity model of autism spectrum disorder pathogenesis: Links to gut bacteria, oxidative stress, and intestinal permeability." Medical Hypotheses 80[[2](#_ENREF_2)]: 264-270.

Henry, A., et al. (2010). "In utero beta 2 adrenergic agonist exposure and adverse neurophysiologic and behavioral outcomes." American Journal of Obstetrics and Gynecology 203[[1](#_ENREF_1)]: e14.

Hepel, M. and M. Stobiecka (2011). Microsensor Arrays for Determination of Biomarkers of Oxidative Stress. Bioelectronics, Biointerfaces, and Biomedical Applications 4. M. Madou, D. Landheer, K. Sode et al. Pennington, Electrochemical Soc Inc. 35: 125-134.

Herbert, M. R. (2007). "Clinical implications of environmental toxicology for children's neurodevelopment in autism." Future Neurology 2(2): 167-171.

Herbert, M. R. (2010). "Contributions of the environment and environmentally vulnerable physiology to autism spectrum disorders." Curr Opin Neurol 23(2): 103-110.

Herbert, M. R. (2011). "Reply to Letter of Peter Good." Neuropsychology Review 21(1, Sp. Iss. SI): 70-71.

Herbert, M. R. and M. P. Anderson (2008). An Expanding Spectrum of Autism Models From Fixed Developmental Defects to Reversible Functional Impairments. Autism: Current Theories and Evidence. A. W. Zimmerman. Totowa, Humana Press Inc: 429-463.

Herder, G. A. (1993). "Infantile autism in Nordland County: Prevalence and etiology." Tidsskrift for den Norske Laegeforening 113(18): 2247-2249.

Herguner, S., et al. (2012). "Ferritin and iron levels in children with autistic disorder." Eur J Pediatr 171[[1](#_ENREF_1)]: 143-146.

Herman, B. H., et al. (1989). "EFFECTS OF ACUTE ADMINISTRATION OF NALTREXONE ON CARDIOVASCULAR FUNCTION BODY TEMPERATURE BODY WEIGHT AND SERUM CONCENTRATIONS OF LIVER ENZYMES IN AUTISTIC CHILDREN." Developmental Pharmacology and Therapeutics 12[[2](#_ENREF_2)]: 118-127.

Herrera, M. and C. A. Smith (2009). "CHED 197-Mapping mercury concentrations in soils near a coal burning power plant." Abstracts of Papers American Chemical Society 238: 197-CHED.

Hertz-Picciotto, I., et al. (2011). "Polybrominated diphenyl ethers in relation to autism and developmental delay: a case-control study." Environmental Health 10: 11.

Hertz-Picciotto, I., et al. (2006). "The CHARGE study: An epidemiologic investigation of genetic and environmental factors contributing to autism." Environmental Health Perspectives 114(7): 1119-1125.

Hertz-Picciotto, I., et al. (2010). "HOUSEHOLD PESTICIDE USE IN RELATION TO AUTISM." American Journal of Epidemiology 171: S100-S100.

Hertz-Picciotto, I., et al. (2008). "Role of metal exposures in autism." Neurotoxicology and Teratology 30[[2](#_ENREF_2)]: 247-248.

Hertz-Picciotto, I., et al. (2010). "Blood mercury concentrations in CHARGE Study children with and without autism." Environ Health Perspect 118[[1](#_ENREF_1)]: 161-166.

Hertz-Picciotto, I., et al. (2008). "Prenatal exposures to persistent and non-persistent organic compounds and effects on immune system development." Basic Clin Pharmacol Toxicol 102(2): 146-154.

Hessel, L. (2003). "[Mercury in vaccines]." Bull Acad Natl Med 187[[1](#_ENREF_1)]: 1501-1510.

Heyer, N. J., et al. (2012). "Disordered Porphyrin Metabolism: A Potential Biological Marker for Autism Risk Assessment." Autism Research 5(2): 84-92.

Higashida, H. (2011). "A missense mutation in CD38 associated with autism spectrum disorder and oxytocin treatment." Neuroscience Research 71: e14.

Hileman, B. (2005). "Biomonitoring." Chemical & Engineering News 83(33): 35-35.

Hill, E. L. (2014). "Linking Clinical and Industrial Psychology: Autism Spectrum Disorder at Work." Industrial and Organizational Psychology-Perspectives on Science and Practice 7[[1](#_ENREF_1)]: 152-155.

Hill, J., et al. (2012). "Environmental toxins linked to neurodegeneration and autism activate the brain's immune system." Journal of the American Pharmacists Association 52(2): 279.

Hill, J. M. (2007). "Vasoactive intestinal peptide in neurodevelopmental disorders: Therapeutic potential." Current Pharmaceutical Design 13[[1](#_ENREF_1)]: 1079-1089.

Hjiej, H., et al. (2008). "[Substitutive and dietetic approaches in childhood autistic disorder: interests and limits]." Encephale 34(5): 496-503.

Hochmann, J. (2012). "The development of child psychiatry through the history of autism as a looking glass." Neuropsychiatrie de l'Enfance et de l'Adolescence 60[[2](#_ENREF_2)]: 207-215.

Hodgson, N. W., et al. (2014). "Decreased glutathione and elevated hair mercury levels are associated with nutritional deficiency-based autism in Oman." Experimental Biology and Medicine 239(6): 697-706.

Hoffman, K., et al. (2012). "The spatial distribution of known predictors of autism spectrum disorders impacts geographic variability in prevalence in central North Carolina." Environmental Health 11: 10.

Holland, N., et al. (2010). "Trans-placental exposures and children's mental and physical health." Environmental and Molecular Mutagenesis 51(7): 694.

Holmes, A. S., et al. (2003). "Reduced levels of mercury in first baby haircuts of autistic children." Int J Toxicol 22[[1](#_ENREF_1)]: 277-285.

Holmes, L. B. (2011). "Human teratogens: Update 2010." Birth Defects Research Part A - Clinical and Molecular Teratology 91[[1](#_ENREF_1)]: 1-7.

Holt, R., et al. (2010). "Linkage and candidate gene studies of autism spectrum disorders in European populations." European Journal of Human Genetics 18[[1](#_ENREF_1)]: 1013-1019.

Holzman, D. C. (2014). "Pesticides and Autism Spectrum Disorders: New Findings from the CHARGE Study." Environ Health Perspect 122(10): A280.

Hong, K. E. M. (2006). "Impacts of rapid social and family changes on the mental health of children in Korea." Psychiatry Investigation 3[[1](#_ENREF_1)]: 6-25.

Hoshino, K. (2012). "Effectiveness of low dose of L-dopamine in children with autism and attention-deficithyperactivity disorder." Developmental Medicine and Child Neurology 54: 148.

House, S. H. (2007). "Nurturing the brain nutritionally and emotionally from before conception to late adolescence." Nutr Health 19(1-2): 143-161.

Hranilovic, D. and M. Bucan (2001). "Social behavior as an endophenotype for psychiatric disorders: Development of mouse models." Current Genomics 2[[1](#_ENREF_1)]: 41-54.

Hu, V. W., et al. (2006). "Gene expression profiling of lymphoblastoid cell lines from monozygotic twins discordant in severity of autism reveals differential regulation of neurologically relevant genes." Bmc Genomics 7: 18.

Huang, C. H., et al. (2004). "Elevated adrenomedullin mRNA in lymphoblastoid cells from schizophrenic patients." Neuroreport 15[[1](#_ENREF_1)]: 1443-1446.

Hughes, J. R. (2008). "A review of recent reports on autism: 1000 studies published in 2007." Epilepsy Behav 13[[2](#_ENREF_2)]: 425-437.

Hughes, J. R. (2009). "Presence of thimerosal in vaccines." Epilepsy and Behavior 14[[1](#_ENREF_1)]: 710.

Hughes, S., et al. (2014). "Differences in reporting serious adverse events in industry sponsored clinical trial registries and journal articles on antidepressant and antipsychotic drugs: A cross-sectional study." BMJ Open 4(7).

Hughes, V. (2006). "News feature: A shot of fear." Nat Med 12[[1](#_ENREF_1)]: 1228-1229.

Hughes, V. (2007). "Mercury rising." Nature Medicine 13[[1](#_ENREF_1)]: 896-897.

Humiston, S. G. (2001). "The stubborn facts of vaccine safety." Pediatric Annals 30(7): 385-390.

Hunter, J. W., et al. (2010). "Neuroligin-deficient mutants of C. elegans have sensory processing deficits and are hypersensitive to oxidative stress and mercury toxicity." Dis Model Mech 3(5-6): 366-376.

Huq, A. M., et al. (2003). "Investigation of 40 positional and functional candidate genes for autism." Annals of Neurology 54(Suppl. 7): S119.

Hussain, J., et al. (2007). "Environmental evaluation of a child with developmental disability." Pediatr Clin North Am 54[[1](#_ENREF_1)]: 47-62, viii.

Hwang, J. Y. and D. C. Pallas (2014). "STRIPAK complexes: Structure, biological function, and involvement in human diseases." International Journal of Biochemistry & Cell Biology 47: 118-148.

Hyman, M. H. (2004). "The impact of mercury on human health and the environment." Altern Ther Health Med 10(6): 70-75.

Hyman, S. E. (2014). "Revitalizing psychiatric therapeutics." Neuropsychopharmacology 39[[1](#_ENREF_1)]: 220-229.

Inampudi, C., et al. (1995). "Regional cerebral blood flow changes in autistic disorder characterized by Tc-99m HMPAO brain SPECT scan." Journal of Investigative Medicine 43(SUPPL. 1): 51A.

Insel, T. (2011). "Who will develop the next generation of medications for mental illness? The NIMH perspective." Neuropsychopharmacology 36: S25.

Ip, P., et al. (2004). "Mercury exposure in children with autistic spectrum disorder: case-control study." J Child Neurol 19(6): 431-434.

Jackson, J. A., et al. (2000). "Case from the center: Comparison of hair copper, zinc, aluminum and lead in patients with elevated and normal urine pyrrole levels." Journal of Orthomolecular Medicine 15[[2](#_ENREF_2)]: 139-140.

Jackson, M. J. and P. J. Garrod (1978). "Plasma zinc, copper, and amino acid levels in the blood of autistic children." J Autism Child Schizophr 8(2): 203-208.

Jain, S. K. (2006). "Oxidative stress and metabolic diseases: Introduction." Pathophysiology 13[[2](#_ENREF_2)]: 127-128.

James, A. (2008). "Pharmacotherapy and child psychiatry: Is there a way forward?" Advances in Psychiatric Treatment 14[[1](#_ENREF_1)]: 10-16.

James, A. C. (2010). "Prescribing antipsychotics for children and adolescents." Advances in Psychiatric Treatment 16[[1](#_ENREF_1)]: 63-75.

James, S. J., et al. (2009). "Cellular and mitochondrial glutathione redox imbalance in lymphoblastoid cells derived from children with autism." Faseb j 23[[1](#_ENREF_1)]: 2374-2383.

Jang, J. N., et al. (2012). "Randomized trial of an eLearning program for training family members of children with autism in the principles and procedures of applied behavior analysis." Research in Autism Spectrum Disorders 6(2): 852-856.

Jen, M. and A. C. Yan (2010). "Syndromes associated with nutritional deficiency and excess." Clin Dermatol 28(6): 669-685.

Jensen, P. S., et al. (2007). "Consensus report on impulsive aggression as a symptom across diagnostic categories in child psychiatry: implications for medication studies." J Am Acad Child Adolesc Psychiatry 46[[2](#_ENREF_2)]: 309-322.

Johnson, S. (2001). "Micronutrient accumulation and depletion in schizophrenia, epilepsy, autism and Parkinson's disease?" Med Hypotheses 56(5): 641-645.

Jonakait, G. M. and L. Ni (2009). "Prostaglandins compromise basal forebrain cholinergic neuron differentiation and survival: Action at EP1/3 receptors results in AIF-induced death." Brain Research 1285: 30-41.

Juhasz, C., et al. (2012). "Quantitative PET Imaging of Tryptophan Accumulation in Gliomas and Remote Cortex: Correlation With Tumor Proliferative Activity." Clinical Nuclear Medicine 37[[1](#_ENREF_1)]: 838-842.

Jurewicz, J. and W. Hanke (2008). "Prenatal and childhood exposure to pesticides and neurobehavioral development: Review of epidemiological studies." International Journal of Occupational Medicine and Environmental Health 21(2): 121-132.

Jusko, T. A., et al. (2011). "Maternal and early postnatal polychlorinated biphenyl exposure in relation to total serum immunoglobulin concentrations in 6-month-old infants." Journal of Immunotoxicology 8[[1](#_ENREF_1)]: 95-100.

Kahler, S. G. (2006). "Dietary and intestinal issues in autism." Neurotoxicology (Amsterdam) 27(6): 1150.

Kahn, J. and G. Fox (1997). "Normal musical development: A review." Medical Problems of Performing Artists 12[[2](#_ENREF_2)]: 83-88.

Kajta, M. and A. Wojtowicz (2010). "[Neurodevelopmental disorders in response to hormonally active environmental pollutants]." Przegl Lek 67[[1](#_ENREF_1)]: 1194-1199.

Kajta, M. and A. K. Wojtowicz (2013). "Impact of endocrine-disrupting chemicals on neural development and the onset of neurological disorders." Pharmacol Rep 65(6): 1632-1639.

Kakubari, N., et al. (1976). "BEHAVIOR THERAPY OF AUTISTIC CHILDREN PART 9 APPLICABILITY AND LIMITATIONS OF THE MONTESSORI EDUCATIONAL MATERIALS." Bulletin of the Seishin-Igaku Institute(20): 1-14.

Kala, G. K., et al. (2009). "Lithium-induced membranous glomerulonephropathy in a pediatric patient." Pediatric Nephrology 24[[1](#_ENREF_1)]: 2267-2269.

Kalia, M. (2008). "Brain development: anatomy, connectivity, adaptive plasticity, and toxicity." Metabolism 57 Suppl 2: S2-5.

Kalkbrenner, A. E., et al. (2011). "RESIDENTIAL MOBILITY OF CHILDREN AND THE POTENTIAL FOR CONTROL SELECTION BIAS." American Journal of Epidemiology 173(Suppl. 11): S184.

Kalkbrenner, A. E., et al. (2014). "Environmental Chemical Exposures and Autism Spectrum Disorders: A Review of the Epidemiological Evidence." Curr Probl Pediatr Adolesc Health Care.

Kamer, A., et al. (2004). "A prevalence estimate of pervasive developmental disorder among immigrants to Israel and Israeli natives- a file review study." Soc Psychiatry Psychiatr Epidemiol 39(2): 141-145.

Kana, R. K., et al. (2009). "Atypical frontal-posterior synchronization of Theory of Mind regions in autism during mental state attribution." Soc Neurosci 4(2): 135-152.

Kane, P. C., et al. (2006). "Clearance of neurotoxins by phospholipid emulsion in autism and PDD." Neurotoxicology (Amsterdam) 27(5, Sp. Iss. SI): 883.

Kappil, M. and J. Chen (2014). "Environmental exposures in utero and microRNA." Current Opinion in Pediatrics 26(2): 243-251.

Karson, C. N., et al. (1996). Method of treating schizophrenia, Tourette's syndrome, mania, autism, and obsessive compulsive disorder with inhibitors of brain nitric oxide synthase, University of Arkansas: 2038.

Kato, T. A., et al. (2013). "Neurotransmitters, Psychotropic Drugs and Microglia: Clinical Implications for Psychiatry." Current Medicinal Chemistry 20[[2](#_ENREF_2)]: 331-344.

Kaufman, D., et al. (2008). "Ethical implications of including children in a large biobank for genetic-epidemiologic research: A qualitative study of public opinion." American Journal of Medical Genetics, Part C: Seminars in Medical Genetics 148[[1](#_ENREF_1)]: 31-39.

Kaur, H., et al. (2013). "Metal ion interactions with drugs: Electrochemical study of complexation of various bivalent metal ions with nimesulide and ibuprofen." Journal of Molecular Liquids 182: 39-42.

Kavirajan, H. (2009). "Memantine: A comprehensive review of safety and efficacy." Expert Opinion on Drug Safety 8[[1](#_ENREF_1)]: 89-109.

Kawada, T. (2014). "Risk Assessment for Autism Spectrum Disorders by Representative Database." Paediatric and Perinatal Epidemiology 28(2): 177-177.

Kawamoto, T., et al. (2011). "Japan environment and children's study." Epidemiology 22: S157-S158.

Kawano, M., et al. (1996). "Retrospective study of general anesthetic management for dental treatment of handicapped patients in Osaka Dental University." Journal of Japanese Dental Society of Anesthesiology 24(2): 325-331.

Kehat, R. and D. J. Bonsall (2009). "Recurrent corneal metallic foreign bodies in children with autism spectrum disorders." J aapos 13(6): 621-622.

Keil, A. P., et al. (2014). "Autism spectrum disorder, flea and tick medication, and adjustments for exposure misclassification: the CHARGE (CHildhood Autism Risks from Genetics and Environment) case-control study." Environ Health 13[[1](#_ENREF_1)]: 3.

Keil, D. E., et al. (2011). "Testing for toxic elements: A focus on arsenic, cadmium, lead, and mercury." Laboratory Medicine 42(12): 735-742.

Kern, J. K. (2003). "Purkinje cell vulnerability and autism: a possible etiological connection." Brain & Development 25(6): 377-382.

Kern, J. K., et al. (2010). "A biomarker of mercury body-burden correlated with diagnostic domain specific clinical symptoms of autism spectrum disorder." Biometals 23(6): 1043-1051.

Kern, J. K., et al. (2011). "Toxicity biomarkers in autism spectrum disorder: a blinded study of urinary porphyrins." Pediatr Int 53(2): 147-153.

Kern, J. K., et al. (2011). "Toxicity biomarkers among US children compared to a similar cohort in France: a blinded study measuring urinary porphyrins." Toxicological and Environmental Chemistry 93(2): 396-405.

Kern, J. K., et al. (2007). "Sulfhydryl-reactive metals in autism." J Toxicol Environ Health A 70[[1](#_ENREF_1)]: 715-721.

Kern, J. K., et al. (2013). "Thimerosal exposure and the role of sulfation chemistry and thiol availability in autism." Int J Environ Res Public Health 10[[1](#_ENREF_1)]: 3771-3800.

Kern, J. K. and A. M. Jones (2006). "Evidence of toxicity, oxidative stress, and neuronal insult in autism." J Toxicol Environ Health B Crit Rev 9(6): 485-499.

Khakzad, M. R., et al. (2012). "The complementary role of high sensitivity C-reactive protein in the diagnosis and severity assessment of autism." Research in Autism Spectrum Disorders 6[[2](#_ENREF_2)]: 1032-1037.

Khan, A., et al. (2014). "Disrupted brain thyroid hormone homeostasis and altered thyroid hormone-dependent brain gene expression in autism spectrum disorders." Journal of Physiology and Pharmacology 65(2): 257-272.

Kidd, P. M. (2002). "Autism, an extreme challenge to integrative medicine. Part 2: medical management." Altern Med Rev 7(6): 472-499.

Kilburn, K. H., et al. "Do terbutaline- and mold-associated impairments of the brain and lung relate to autism?" Toxicol Ind Health.

Kim, H. W., et al. (2009). "Family-based association study between NOS-I and -IIA polymorphisms and autism spectrum disorders in Korean trios." Am J Med Genet B Neuropsychiatr Genet 150b(2): 300-306.

Kim, P., et al. "Effects of Korean red ginseng extracts on neural tube defects and impairment of social interaction induced by prenatal exposure to valproic acid." Food Chem Toxicol.

Kim, S. A. and S. Y. Yang (2010). "Focused Conference Group: P15 - Endothelium in health and disease functional analysis of single nucleotide polymorphsims in NOS2A." Basic and Clinical Pharmacology and Toxicology 107: 377.

Kimmel, C. A., et al. (2005). "Lessons learned for the national children's study from the National Institute of Environmental Health Sciences/US Environmental Protection Agency Centers for Children's Environmental Health and Disease Prevention Research." Environmental Health Perspectives 113(10): 1414-1418.

Kimmel, S. R. (2002). "Vaccine adverse events: separating myth from reality." Am Fam Physician 66[[1](#_ENREF_1)]: 2113-2120.

Kimmel, S. R. (2003). "A mother who refuses to vaccinate her child." American Family Physician 67[[2](#_ENREF_2)]: 651+655-656.

King, B. H. (2014). "Sunsetting DSM-IV's pervasive developmental disorder." Journal of the American Academy of Child and Adolescent Psychiatry 53(5): 494-496.

Kinney, D. K., et al. (2010). "Environmental risk factors for autism: Do they help cause de novo genetic mutations that contribute to the disorder?" Medical Hypotheses 74[[1](#_ENREF_1)]: 102-106.

Kita, T., et al. (2003). "Current research on methamphetamine-induced neurotoxicity: animal models of monoamine disruption." J Pharmacol Sci 92[[2](#_ENREF_2)]: 178-195.

Koenig, C. M., et al. (2012). "Lack of Evidence for Neonatal Misoprostol Neurodevelopmental Toxicity in C57BL6/J Mice." Plos One 7(6): 7.

Kojima, H. and G. Yasukochi (1973). "Treatment with L-DOPA in two cases of organic brain syndrome." Folia Psychiatrica et Neurologica Japonica 27(2): 155-166.

Kream, R. M., et al. (2010). "Psychiatric Implications of Endogenous Morphine: Up-To-Date Review." Folia Biologica 56(6): 231-241.

Kuehn, B. M. (2006). "NIH initiatives to probe contribution of genes, environment in disease." Journal of the American Medical Association 295[[1](#_ENREF_1)]: 1633-1634.

Kuipers, R. S., et al. (2012). "A multidisciplinary reconstruction of Palaeolithic nutrition that holds promise for the prevention and treatment of diseases of civilisation." Nutrition Research Reviews 25[[1](#_ENREF_1)]: 96-129.

Kuji, A., et al. (2007). "Usefulness of propofol and laryngeal mask airway in ambulatory anesthesia - A study on 100 cases of outpatient anesthesia for the handicapped." Journal of Japanese Dental Society of Anesthesiology 35[[2](#_ENREF_2)]: 365-372.

Kultur, S. E. C. (2011). "Zinc supplementation in psychiatric disorders of children." Klinik Psikofarmakoloji Bulteni 21: S73.

Kurt, I., et al. (2011). "Urinary porphyrins and biomarkers of oxidative stress in Turkish autistic children." Journal of Inherited Metabolic Disease 34: S270.

Kuwabara, H., et al. (2013). "Altered Metabolites in the Plasma of Autism Spectrum Disorder: A Capillary Electrophoresis Time-of-Flight Mass Spectroscopy Study." Plos One 8[[1](#_ENREF_1)]: 8.

Kuwagata, M. (2012). "Current problems of in vivo developmental neurotoxicity tests and a new in vivo approach focusing on each step of the developing central nervous system." Congenital Anomalies 52[[2](#_ENREF_2)]: 129-139.

Kwan, K. Y., et al. (2012). "Species-dependent posttranscriptional regulation of NOS1 by FMRP in the developing cerebral cortex." Cell 149[[1](#_ENREF_1)]: 899-911.

Labie, D. (2007). "[Developmental neurotoxicity of industrial chemicals]." Med Sci (Paris) 23(10): 868-872.

Lai, M. W. and M. Burns Ewald (2005). "Pediatric "pesticide" poisoning in a pill: Predominant nicotinic cholinergic effects after exposure to a therapeutic carbamate." Clinical Toxicology 43(6): 643.

Laks, D. R. (2010). "Luteinizing hormone provides a causal mechanism for mercury associated disease." Medical Hypotheses 74[[1](#_ENREF_1)]: 698-701.

Lakshmi Priya, M. D. and A. Geetha (2011). "A biochemical study on the level of proteins and their percentage of nitration in the hair and nail of autistic children." Clin Chim Acta 412(11-12): 1036-1042.

Lakshmi Priya, M. D. and A. Geetha (2011). "Level of trace elements (copper, zinc, magnesium and selenium) and toxic elements (lead and mercury) in the hair and nail of children with autism." Biol Trace Elem Res 142(2): 148-158.

Lamb, J. C., et al. (2014). "Critical comments on the WHO-UNEP State of the Science of Endocrine Disrupting Chemicals - 2012." Regulatory Toxicology and Pharmacology 69[[1](#_ENREF_1)]: 22-40.

Lamm, S. H. and C. Goebel (2004). "Perchlorate in the environment: The epidemiological studies find no adverse outcome." Abstracts of Papers American Chemical Society 228(Part 1): U89.

Lammers, H. (2003). "Autism due to mercury load?" Zeitschrift fur Umweltmedizin 11(2): 70-75.

Lan, A., et al. (2013). "Effects of gestational exposure to chlorpyrifos on motor development and social behavior in C57BL/6 J mice." Journal of Molecular Neuroscience 51: S65-S66.

Landrigan, P. (2009). "Environmental disasters and children's health." Neurotoxicology and Teratology 31[[1](#_ENREF_1)]: 245-246.

Landrigan, P. J. (2009). "Environmental Disasters and Children's Health." Birth Defects Research 85(5): 387.

Landrigan, P. J. (2010). "What causes autism? Exploring the environmental contribution." Current Opinion in Pediatrics 22(2): 219-225.

Landrigan, P. J. (2012). "The hidden costs of environmental contamination." European Respiratory Journal 40(2): 286-288.

Landrigan, P. J. (2012). "The national children's study and its relationship to prospective international studies in child health." Birth Defects Research Part A - Clinical and Molecular Teratology 94(5): 379.

Landrigan, P. J. and A. Garg (2002). "Chronic effects of toxic environmental exposures on children's health." Journal of Toxicology - Clinical Toxicology 40[[1](#_ENREF_1)]: 449-456.

Landrigan, P. J., et al. (2012). "A research strategy to discover the environmental causes of autism and neurodevelopmental disabilities." Environmental Health Perspectives 120(7): A258-A260.

Landrigan, P. J. and G. Tamburlini (2005). "Children's health and the environment: A transatlantic dialogue." Environmental Health Perspectives 113(10): A646-A647.

Landrigan, P. J. and L. Trasande (2010). "The us national children's study: A 21-year prospective study of 100,000 american children." Acta Paediatrica, International Journal of Paediatrics 99: 11-12.

Landrigan, P. J., et al. (2006). "The national children's study: A 21-year prospective study of 100 000 American children." Pediatrics 118(5): 2173-2186.

Larsson, M., et al. (2009). "Associations between indoor environmental factors and parental-reported autistic spectrum disorders in children 6-8 years of age." Neurotoxicology 30(5): 822-831.

LaSalle, J. M. (2011). "A genomic point-of-view on environmental factors influencing the human brain methylome." Epigenetics 6(7): 862-869.

Lasalle, J. M. (2013). "Epigenomic strategies at the interface of genetic and environmental risk factors for autism." Journal of Human Genetics 58(7): 396-401.

Lathe, R. (2006). "Author's reply to review of his book on autism [2]." British Medical Journal 333(7563): 352.

Lathe, R. (2008). "Environmental factors and limbic vulnerability in childhood autism." American Journal of Biochemistry and Biotechnology 4(2): 183-197.

Lathe, R. and M. Le Page (2003). "Toxic metal clue to autism - A study has revealed startling differences in mercury levels in the hair of autistic and normal children." New Scientist 178(2400): 4-5.

Laugeray, A., et al. (2014). "Perinatal exposure to low dose glufosinate ammonium induces autism-like phenotypes in mice." Toxicology Letters (Shannon) 229(Suppl. S): S43.

Laura, V., et al. (2011). "Metals, metallothioneins and oxidative stress in blood of autistic children." Research in Autism Spectrum Disorders 5[[1](#_ENREF_1)]: 286-293.

Lauritsen, M. B., et al. (2014). "Urbanicity and autism spectrum disorders." J Autism Dev Disord 44(2): 394-404.

Laviola, G., et al. (2006). "Paradoxical effects of prenatal acetylcholinesterase blockade on neuro-behavioral development and drug-induced stereotypies in reeler mutant mice." Psychopharmacology (Berl) 187[[2](#_ENREF_2)]: 331-344.

Laviola, G., et al. (2009). "Gene-environment interaction during early development in the heterozygous reeler mouse: Clues for modelling of major neurobehavioral syndromes." Neuroscience & Biobehavioral Reviews 33(4, Sp. Iss. SI): 560-572.

Lawler, C. P. (2008). "The "Environment" for autism research: Signs of improvement?" Environmental Health Perspectives 116(10): 416-417.

Lawler, C. P., et al. (2004). "Identifying environmental contributions to autism: Provocative clues and false leads." Mental Retardation and Developmental Disabilities Research Reviews 10[[1](#_ENREF_1)]: 292-302.

Lazzaro, R. J. (2007). "Don't shy away from vaccination [[1](#_ENREF_1)]." Drug Topics 151(5).

Le Saux, N. (2009). "Dispelling myths held by parents about the influenza vaccine." Paediatrics and Child Health 14[[1](#_ENREF_1)]: 618-620.

Leask, J. and P. McIntyre (2003). "Public opponents of vaccination: A case study." Vaccine 21(32): 4700-4703.

Leavey, A., et al. (2013). "Gestational age at birth and risk of autism spectrum disorders in Alberta, Canada." Journal of Pediatrics 162(2): 361-368.

Leckman, J. F. and A. E. Herman (2002). "Maternal behavior and developmental psychopathology." Biol Psychiatry 51[[1](#_ENREF_1)]: 27-43.

Lee, D. A., et al. (2003). "Childhood autism: A circuit syndrome?" Neurologist 9(2): 99-109.

Lee, M. C., et al. (1990). "TECHNETIUM-99M-LABELED HMPAO BRAIN SPECT STUDY OF REGIONAL BLOOD FLOW IN PATIENTS WITH INFANTILE AUTISM." European Journal of Nuclear Medicine 16(7): 522.

Lee, Y. and D. Styne (2013). "Influences on the onset and tempo of puberty in human beings and implications for adolescent psychological development." Hormones and Behavior 64(2): 250-261.

Leeder, J. S. (2003). "Developmental and pediatric pharmacogenomics." Pharmacogenomics 4[[2](#_ENREF_2)]: 331-341.

Lees, H. J., et al. (2013). "Hippurate: The natural history of a mammalian-microbial cometabolite." Journal of Proteome Research 12[[1](#_ENREF_1)]: 1527-1546.

Leonard, M. M. and B. Vasagar (2014). "US perspective on gluten-related diseases." Clinical and Experimental Gastroenterology 7[[1](#_ENREF_1)]: 25-37.

Leslie, K. E. and S. M. Koger (2011). "A Significant Factor in Autism: Methyl Mercury Induced Oxidative Stress in Genetically Susceptible Individuals." Journal of Developmental and Physical Disabilities 23[[1](#_ENREF_1)]: 313-324.

Levin, E. D., et al. (2009). "Genetic aspects of behavioral neurotoxicology." NeuroToxicology 30(5): 741-753.

Levitsky, L. L. (2004). "Childhood Immunizations and Chronic Illness." New England Journal of Medicine 350[[1](#_ENREF_1)]: 1380-1382.

Levy, S. E. and S. L. Hyman (2003). "Use of complementary and alternative treatments: For children with autistic spectrum disorders is increasing." Pediatric Annals 32(10): 685-691.

Levy, S. E. and S. L. Hyman (2008). "Complementary and Alternative Medicine Treatments for Children with Autism Spectrum Disorders." Child and Adolescent Psychiatric Clinics of North America 17[[1](#_ENREF_1)]: 803-820.

Lewandowski, T. A. (2006). "Questions regarding environmental mercury release, special education rates, and autism disorder: An ecological study of Texas by Palmer et al." Health and Place 12[[1](#_ENREF_1)]: 749-750.

Lewandowski, T. A. (2010). EVOLVING UNDERSTANDING OF THE RELATIONSHIP BETWEEN MERCURY EXPOSURE AND AUTISM. Environmental Heavy Metal Pollution and Effects on Child Mental Development: Risk Assessment and Prevention Strategies. L. I. Simeonov, M. V. Kochubovski and B. G. Simeonova. Dordrecht, Springer: 65-84.

Li, X. and M. DiFiglia (2012). "The recycling endosome and its role in neurological disorders." Progress in Neurobiology 97(2): 127-141.

Li, X., et al. (2014). "The ADAR RNA editing enzyme controls neuronal excitability in Drosophila melanogaster." Nucleic Acids Research 42(2): 1139-1151.

Lidsky, T. I. and J. S. Schneider (2005). "Autism and autistic symptoms associated with childhood lead poisoning." Journal of Applied Research 5[[1](#_ENREF_1)]: 80-87.

Liew, Z., et al. (2013). "Perfluorinated chemicals in maternal serum and the risk of autism, attention-deficit/hyperactivity disorder and cerebral palsy in children." European Journal of Epidemiology 28[[1](#_ENREF_1)]: S174.

Lloyd-Smith, M. and B. Sheffield-Brotherton (2008). "Children's environmental health: intergenerational equity in action--a civil society perspective." Ann N Y Acad Sci 1140: 190-200.

Lobanenkov, V., et al. (2011). "Environmental epigenomics and disease susceptibility." Epigenomics 3[[2](#_ENREF_2)]: 261-266.

Lombard, J. (1998). "Autism: a mitochondrial disorder?" Med Hypotheses 50(6): 497-500.

London, E. and R. A. Etzel (2000). "The environment as an etiologic factor in autism: A new direction for research." Environmental Health Perspectives 108(Supplement 3): 401-404.

Long, M., et al. (2014). "Endocrine disrupting compounds and heavy metals in the amniotic fluid and neurodevelopment disorders: A case-control study." Toxicology Letters (Shannon) 229(Suppl. S): S121.

Lonsdale, D., et al. (2002). "Treatment of autism spectrum children with thiamine tetrahydrofurfuryl disulfide: a pilot study." Neuro Endocrinol Lett 23[[1](#_ENREF_1)]: 303-308.

Lonsdale, D., et al. (2011). "Dysautonomia in autism spectrum disorder: case reports of a family with review of the literature." Autism Res Treat 2011: 129795.

Lopes, M. M. and L. Q. A. Caldas (2011). "Young children with austim spectrum disorders: Can aluminium bodyburden cause metabolism disruption?" Toxicology Letters 205: S92.

Lopez-Rodriguez, A. and M. Holmgren (2012). "Restoration of proper trafficking to the cell surface for membrane proteins harboring cysteine mutations." PLoS One 7(10): e47693.

Low, H., et al. (2012). "Putting together a plasma membrane NADH oxidase: a tale of three laboratories." Int J Biochem Cell Biol 44[[1](#_ENREF_1)]: 1834-1838.

Lu, J. M., et al. (2010). "Chemical and molecular mechanisms of antioxidants: experimental approaches and model systems." Journal of Cellular and Molecular Medicine 14[[1](#_ENREF_1)]: 840-860.

Lu, J. M., et al. (2012). "Ginsenoside Rb1 Directly Scavenges Hydroxyl Radical and Hypochlorous Acid." Current Pharmaceutical Design 18(38): 6339-6347.

Lucas, K. and M. Maes (2013). "Role of the Toll Like receptor (TLR) radical cycle in chronic inflammation: possible treatments targeting the TLR4 pathway." Mol Neurobiol 48[[1](#_ENREF_1)]: 190-204.

Luck, A. N., et al. (2013). "Human serum transferrin: is there a link among autism, high oxalate levels, and iron deficiency anemia?" Biochemistry 52(46): 8333-8341.

Luna, G., et al. (2011). "Possible role of mixed heavy metal toxicity in recurrent infections and NK cell count in a population of children with different forms of immune dysfunction." Allergy (Oxford) 66(Suppl. 94, Sp. Iss. SI): 548.

Lyall, K., et al. (2014). "Maternal lifestyle and environmental risk factors for autism spectrum disorders." Int J Epidemiol 43(2): 443-464.

Macedoni-Luksic, M. (2012). "Biochemical markers in autism spectrum disorders." European Journal of Paediatric Neurology 16(5): 557.

Macedoni-Luksic, M., et al. (2014). "Levels of Metals in the Blood and Specific Porphyrins in the Urine in Children with Autism Spectrum Disorders." Biol Trace Elem Res.

MacFabe, D. F., et al. (2007). "Neurobiological effects of intraventricular propionic acid in rats: Possible role of short chain fatty acids on the pathogenesis and characteristics of autism spectrum disorders." Behavioural Brain Research 176[[1](#_ENREF_1)]: 149-169.

Macfarlane, G. T. and S. Macfarlane (2012). "Bacteria, Colonic Fermentation, and Gastrointestinal Health." Journal of Aoac International 95[[1](#_ENREF_1)]: 50-60.

Maggon, K. (2009). "Industrial R&D paradigm shift to vaccines." Biotechnology Journal 4[[1](#_ENREF_1)]: 458-461.

Main, P. A., et al. (2013). "Necrosis is increased in lymphoblastoid cell lines from children with autism compared with their non-autistic siblings under conditions of oxidative and nitrosative stress." Mutagenesis 28[[1](#_ENREF_1)]: 475-484.

Majewska, M. D., et al. (2010). "Different levels of salivary steroids in autistic and healthy children." European Neuropsychopharmacology 20: S615-S616.

Majewska, M. D., et al. (2010). "Age-dependent lower or higher levels of hair mercury in autistic children than in healthy controls." Acta Neurobiol Exp (Wars) 70(2): 196-208.

Mancardi, M. M., et al. (2006). "Creatine transporter deficiency and epilepsy: Description of two cases and brief review of the literature." Bollettino - Lega Italiana contro l'Epilessia(133-134): 275-277.

Mania, M., et al. (2012). "[Fish and seafood as a source of human exposure to methylmercury]." Rocz Panstw Zakl Hig 63[[2](#_ENREF_2)]: 257-264.

Manning-Courtney, P., et al. (2003). "Diagnosis and treatment of autism spectrum disorders." Current Problems in Pediatric and Adolescent Health Care 33[[1](#_ENREF_1)]: 283-304.

Manto, M. U. and H. Fatemi (2004). "Nitric oxide in the cerebellum of mutant mice." Cerebellum 3[[2](#_ENREF_2)]: 130-132.

Manuel, J. (2013). "The Long Road to Recovery Environmental Health Impacts of Hurricane Sandy." Environmental Health Perspectives 121(5): A152-A159.

Marger, L., et al. (2014). "Zinc: An underappreciated modulatory factor of brain function." Biochemical Pharmacology 91[[1](#_ENREF_1)]: 426-435.

Margolis, F. S. (2009). "Fact or fiction?" Alpha Omegan 102(2): 43-44.

Marin, P., et al. (2014). "Proteomics and phosphoproteomics for understanding cellular signalling and its disruption in CNS disorders." European Neuropsychopharmacology 24: S2-S3.

Maritzen, T., et al. (2012). "Turning CALM into excitement: AP180 and CALM in endocytosis and disease." Biology of the Cell 104(10): 588-602.

Marriott, A. L., et al. (2013). New Animal Models of Progressive Neurodegeneration: Tools for Developing Predictive Diagnostics and Identifying Presymptomatic Therapeutic Targets. 3: 45-68.

Marta, M. L., et al. (2014). "Heavy metals and specific porphyrine levels in children with autism." Zdravniski Vestnik 83(5): 376-386.

Martin, B. J. (2009). "Re: Biomarkers of environmental toxicity and susceptibility in autism." J Neurol Sci 280(1-2): 127-128; author reply 128-129; discussion 129-130.

Martin, M. D. and J. S. Woods (2006). "The safety of dental amalgam in children." Expert Opin Drug Saf 5(6): 773-781.

Marx, G. and C. Gilon (2012). "The molecular basis of memory." ACS Chem Neurosci 3[[1](#_ENREF_1)]: 633-642.

Masters, R. D. (2006). "The gap between neurotoxicology and public policy: Case studies of environmental toxins and neurodevelopmental disorders." Neurotoxicology (Amsterdam) 27(5, Sp. Iss. SI): 930.

Mathew, N. M. (2011). "Exposure to freeway air pollution and developmental delays." Retrieved 11/13/2014, from http://cdm15799.contentdm.oclc.org/cdm/ref/collection/p15799coll127/id/623048.

Matsumoto, J., et al. (2014). "Mental disorders that exacerbated due to the Fukushima disaster, a complex radioactive contamination disaster." Psychiatry and Clinical Neurosciences 68[[2](#_ENREF_2)]: 182-187.

Maughan, B., et al. (2005). "Time trends in child and adolescent mental disorders." Curr Opin Psychiatry 18[[1](#_ENREF_1)]: 381-385.

Mazur-Kolecka, B., et al. (2007). "Altered development of neuronal progenitor cells after stimulation with autistic blood sera." Brain Research 1168: 11-20.

McCarthy, M. "Conflict of interest taints vaccine approval process, charges US report." Lancet.

McCormick, M. C. (2003). "The autism "epidemic": Impressions from the perspective of immunization safety review." Ambulatory Pediatrics 3[[2](#_ENREF_2)]: 119-120.

McFadden, S. A. (1996). "Phenotypic variation in xenobiotic metabolism and adverse environmental response: Focus on sulfur-dependent detoxification pathways." Toxicology 111(1-3): 43-65.

McGinnis, W. R. (2001). "Mercury and autistic gut disease." Environ Health Perspect 109(7): A303-304.

McGinnis, W. R. (2004). "Oxidative stress in autism." Alternative Therapies in Health and Medicine 10(6): 22-36.

McGinnis, W. R., et al. (2013). "Proposed toxic and hypoxic impairment of a brainstem locus in autism." Int J Environ Res Public Health 10(12): 6955-7000.

McGovern, V. (2007). "Autism and agricultural pesticides. Integrating data to track trends." Environ Health Perspect 115(10): A504.

McKay, C. (2013). "Commentary on "Transdermal DMPS"." Journal of Medical Toxicology 9[[1](#_ENREF_1)]: 1-3.

McLennan, J. D., et al. (1993). "Sex differences in higher functioning people with autism." Journal of Autism and Developmental Disorders 23(2): 217-227.

Meadows, M. (2004). "IOM report: no link between vaccines and autism." FDA Consum 38(5): 18-19.

Medrihan, L., et al. (2009). "Neurobeachin, a protein implicated in membrane protein traffic and autism, is required for the formation and functioning of central synapses." J Physiol 587(Pt 21): 5095-5106.

Meeker, J. D. (2012). "Exposure to environmental endocrine disruptors and child development." Archives of Pediatrics and Adolescent Medicine 166(10): 952-958.

Mei, H., et al. (2012). "Opportunities in systems biology to discover mechanisms and repurpose drugs for CNS diseases." Drug Discovery Today 17(21-22): 1208-1216.

Meletis, C. D. and N. Zabriskie (2007). "Is autism the coal miner's canary of America's health status?" Alternative and Complementary Therapies 13[[1](#_ENREF_1)]: 193-198.

Messer, A. (2010). "Mini-review: polybrominated diphenyl ether (PBDE) flame retardants as potential autism risk factors." Physiol Behav 100[[2](#_ENREF_2)]: 245-249.

Meyers, O. I. (2013). "The impact of DSM-5 on the development of drugs to treat autism spectrum disorder." Value in Health 16(7): A607.

Miles, J. H. and T. N. Takahashi (2007). "Lack of association between Rh status, Rh immune globulin in pregnancy and autism." American Journal of Medical Genetics, Part A 143(13): 1397-1407.

Miles, J. H. and T. N. Takahashi (2008). "Reply to Bernard et al. "Re: Miles and Takahashi paper on RHIg and autism" [[1](#_ENREF_1)]." American Journal of Medical Genetics, Part A 146[[2](#_ENREF_2)]: 407.

Miller, C. (2006). "An Emerging Paradigm. Chemical Exposures and Health." Explore: The Journal of Science and Healing 2(5): 432-441.

Miller, M. D., et al. (2002). "Differences between children and adults: Implications for risk assessment at California EPA." International Journal of Toxicology 21(5): 403-418.

Minami, T., et al. (2010). "Induction of metallothionein in mouse cerebellum and cerebrum with low-dose thimerosal injection." Cell Biology and Toxicology 26(2): 143-152.

Minami, T., et al. (2007). "Effects of lipopolysaccharide and chelator on mercury content in the cerebrum of thimerosal-administered mice." Environ Toxicol Pharmacol 24[[2](#_ENREF_2)]: 316-320.

Ming, X., et al. (2010). "Genetic variant of glutathione peroxidase 1 in autism." Brain & Development 32(2): 105-109.

Ming, X., et al. (2005). "Increased excretion of a lipid peroxidation biomarker in autism." Prostaglandins Leukotrienes and Essential Fatty Acids 73(5): 379-384.

Mishori, R. and C. McHale (2014). "Pica: An age-old eating disorder that's often missed." Journal of Family Practice 63(7): E1-E4.

Miwa, S., et al. (1992). "[A novel function of tetrahydrobiopterin]." Nihon Yakurigaku Zasshi 100(5): 367-381.

Moffitt, T. E. and M. Melchior (2007). "Why does the worldwide prevalence of childhood attention deficit hyperactivity disorder matter?" American Journal of Psychiatry 164(6): 856-858.

Mohammed, S., et al. (2014). "A review on the impact of the environmental adversities on various developmental disorders of brain in children." International Journal of Pharmacy and Pharmaceutical Sciences 6(6): 161-164.

Mondal, S., et al. (2010). "A synthetic ditryptophan conjugate that rescues bacteria from mercury toxicity through complexation." Tetrahedron Letters 51(47): 6111-6115.

Monro, J. A., et al. (2013). "The risk of lead contamination in bone broth diets." Med Hypotheses 80[[1](#_ENREF_1)]: 389-390.

Moolman-Smook, J. C., et al. (2008). "Of rain men and snowcakes: The presentation, pathology, aetiology and management of autistic spectrum disorder." SAJCH South African Journal of Child Health 2[[1](#_ENREF_1)]: 8-12.

Moore, J. H. and J. Hwa (2014). "Editorial: pharmacogenetics and molecular medicine: "so close and yet so far"." Curr Mol Med 14(7): 803-804.

Moore, M. W., et al. (2012). "Challenges in the codevelopment of companion diagnostics." Personalized Medicine 9(5): 485-496.

Moraes, C. T., et al. (2013). "Translational research in primary mitochondrial diseases: challenges and opportunities." Mitochondrion 13(6): 945-952.

Moraes, F. C., et al. (2012). "Glass/PDMS hybrid microfluidic device integrating vertically aligned SWCNTs to ultrasensitive electrochemical determinations." Lab on a Chip - Miniaturisation for Chemistry and Biology 12[[1](#_ENREF_1)]: 1959-1962.

Morara, M., et al. (2007). "Optimal design for epidemiological studies subject to designed missingness." Lifetime Data Anal 13[[1](#_ENREF_1)]: 583-605.

Morris, S. A. and H. H. Bernstein (2004). "Immunizations, neonatal jaundice, and animal-induced injuries." Current Opinion in Pediatrics 16[[1](#_ENREF_1)]: 450-460.

Moss, M. and D. Downing (2008). "British Society for Ecological Medicine Winter Meeting November 2007: Fats and membranes." Journal of Nutritional and Environmental Medicine 17[[1](#_ENREF_1)]: 1-11.

Mount, R. H., et al. (2003). "Features of autism in Rett syndrome and severe mental retardation." Journal of Autism and Developmental Disorders 33[[1](#_ENREF_1)]: 435-442.

Mountz, J. M., et al. (1993). "Technetium-99m HM-PAO brain SPECT evaluation of autistic disorder." Journal of Nuclear Medicine 34(5 SUPPL.): 78P.

Mullen, B., et al. (2010). "A Dab1-lacZ reporter reveals CNS lamination defects in a mouse model for autism." Society for Neuroscience Abstract Viewer and Itinerary Planner 40.

Mullen, B. R., et al. (2013). "Decreased reelin expression and organophosphate pesticide exposure alters mouse behaviour and brain morphology." ASN Neuro 5[[1](#_ENREF_1)]: e00106.

Mundschenk, N. A., et al. (2011). "Effective Classroom Management: An Air Traffic Control Analogy." Intervention in School and Clinic 47(2): 98-103.

Murphy, D. and W. Spooren (2012). "EU-AIMS: a boost to autism research." Nat Rev Drug Discov 11[[1](#_ENREF_1)]: 815-816.

Murphy, S. K. (2014). "Obesity: Paternal obesity - A risk factor for autism?" Nature Reviews Endocrinology 10(7): 389-390.

Muskiet, F. A. J. (2010). Frontiers in Neuroscience

Pathophysiology and Evolutionary Aspects of Dietary Fats and Long-Chain Polyunsaturated Fatty Acids across the Life Cycle. Fat Detection: Taste, Texture, and Post Ingestive Effects. J. P. Montmayeur and J. le Coutre. Boca Raton (FL), CRC Press

Taylor & Francis Group, LLC.

Mutter, J., et al. (2007). "Comments on the article "the toxicology of mercury and its chemical compounds" by Clarkson and Magos (2006)." Crit Rev Toxicol 37(6): 537-549; discussion 551-532.

Mutter, J., et al. (2005). "Mercury and autism: accelerating evidence?" Neuro Endocrinol Lett 26(5): 439-446.

Mutter, J., et al. (2005). "[Amalgam risk assessment with coverage of references up to 2005]." Gesundheitswesen 67[[2](#_ENREF_2)]: 204-216.

Mychaskiw 2nd, G. (2010). "Hyperbaric oxygen therapy and neurologic disease: the time has come." Undersea & hyperbaric medicine : journal of the Undersea and Hyperbaric Medical Society, Inc 37(2): xi-xiii.

Mychaskiw, G., II (2011). "Neurologic applications of hyperbaric oxygen: A sad, slow story of smoke and fire." Undersea & Hyperbaric Medicine 38(5): 305-307.

Nalli, C., et al. (2014). "The effects of lupus and antiphospholipid antibody syndrome on foetal outcomes." Lupus 23(6): 507-517.

Nash, D. T. and A. R. Slutzky (2014). "Gluten Sensitivity: New Epidemic or New Myth? Every Major Change in Our Diet Carries With It the Possibility of Unforeseen Risks." Am J Cardiol.

Nash, R. A. (2005). "Metals in medicine." Integrative Medicine 4[[1](#_ENREF_1)]: 38-47.

Nataf, R., et al. (2006). "Porphyrinuria in childhood autistic disorder: implications for environmental toxicity." Toxicol Appl Pharmacol 214(2): 99-108.

National Environmental Trust, W., DC.; Environmental Protection Agency, Washington, DC.; Physicians for Social Responsibility, Washington, DC.; Learning Disabilities Association of America, Pittsburgh, PA. (2000). Polluting Our Future. Chemical Pollution in the U.S. that Affects Child Development and Learning. .

Nau, J. Y. (2012). "[Autism and antibiotics: the Montagnier question]." Rev Med Suisse 8(335): 772-773.

Naviaux, R. K. (2014). "Metabolic features of the cell danger response." Mitochondrion 16: 7-17.

Nelson, K. B. and M. L. Bauman (2003). "Thimerosal and autism?" Pediatrics 111[[2](#_ENREF_2)]: 674-679.

Nelson, R. J. (2012). "Role of Nitric Oxide in Aggression and Social Behavior." Biological Psychiatry 71(8, Suppl. S): 124S.

Nevison, C. D. (2014). "A comparison of temporal trends in United States autism prevalence to trends in suspected environmental factors." Environ Health 13: 73.

Newschaffer, C. J., et al. (2012). "Infant siblings and the investigation of autism risk factors." Journal of Neurodevelopmental Disorders 4: 16.

Newton, K. E., et al. (2005). "A problem with her lead weight." Ann Clin Biochem 42(Pt 2): 145-148.

Ng, D. K., et al. (2007). "Low-level chronic mercury exposure in children and adolescents: meta-analysis." Pediatr Int 49[[1](#_ENREF_1)]: 80-87.

Nicoll, A. (2001). "Benefits, safety, and risks of immunisation programmes." Interdisciplinary Science Reviews 26[[1](#_ENREF_1)]: 20-30.

Nicolle-Mir, L. (2014). "Perinatal exposure to atmospheric pollutants and the risk of autism." Environnement, Risques et Sante 13(2): 98-99.

Nietupski, J. A., et al. (2001). "Preparing students with mild disabilities for careers in technology: A process and recommendations from Iowa's High School High Tech program." Journal of Vocational Rehabilitation 16(3-4): 179-187.

Nilsen, R. M., et al. (2014). "Reply to T. Kawada." Paediatric and Perinatal Epidemiology 28(2): 178.

Nolfe, G. (2012). "EEG and meditation." Clinical Neurophysiology 123[[1](#_ENREF_1)]: 631-632.

Obrenovich, M. E., et al. (2011). "Altered heavy metals and transketolase found in autistic spectrum disorder." Biol Trace Elem Res 144(1-3): 475-486.

Ohnishi, T., et al. (2000). "Abnormal regional cerebral blood flow in childhood autism." Brain 123[[1](#_ENREF_1)]: 1838-1844.

Ohno, H., et al. (2004). "Wide use of Merthiolate may cause mercury poisoning in Mexico." Bulletin of Environmental Contamination and Toxicology 73(5): 777-780.

Okawa, T., et al. (2007). "Evaluation of outpatient inhalation guidance for infant childhood asthma." Oto-Rhino-Laryngology Tokyo 50(SUPPL. 3): 13-28.

Olivan Gonzalvo, G. (2008). "Psychomotor delay in children adopted in Russia is not associated with lead." Anales de Pediatria 69(5): 495-496.

Omura, Y. (2006). "Asbestos as a possible major cause of malignant lung tumors (including small cell carcinoma, adenocarcinoma & mesothelioma), brain tumors (i.e. astrocytoma & glioblastoma multiforme), many other malignant tumors, intractable pain including fibromyalgia, & some cardio-vascular pathology: Safe & effective methods of reducing asbestos from normal & pathological areas." Acupunct Electrother Res 31(1-2): 61-125.

Omura, Y. (2008). "Very recently discovered role of asbestos in intractable medical problems including malignant tumors, cardiovascular diseases, Alzheimer's disease, autism, cataracts, intractable pain, and Morgellon's disease (which is considered to be a medical mystery) and how to remove asbestos from the human body safely and effectively." Acupuncture & Electro-Therapeutics Research 33(1-2): 51-53.

Ondine, v. E., et al. (2013). Prenatal Exposure to Air Toxics and Autism in Children. 27th Conference of the International Society for Environmental Epidemiology (ISEE) "Addressing Environmental Health Inequalities", Centro de Convencoes Reboucas, Sao Paulo, Brazil.

Orenstein, W. A., et al. (2005). "Immunizations in the United States: Success, structure, and stress - A complex collaboration involving government, industry, providers, academe, professional societies, and third-party payers." Health Affairs 24[[2](#_ENREF_2)]: 599-610.

Ornoy, A. (2009). "Valproic acid in pregnancy: How much are we endangering the embryo and fetus?" Reproductive Toxicology 28[[1](#_ENREF_1)]: 1-10.

Ortega Garcia, J. A., et al. (2011). "Prenatal exposure of a girl with autism spectrum disorder to 'horsetail' (Equisetum arvense) herbal remedy and alcohol: A case report." Journal of Medical Case Reports 5.

Osborne, J. W. and J. Summitt (2003). "Autism Caused by Dental Amalgam?" Operative Dentistry 28(6): 665-666.

Osinka, A. and K. Pasternak (2014). "MERCURY IN MEDICINE AND HEALTH SERVICE." Journal of Elementology 19[[1](#_ENREF_1)]: 289-297.

Ouhaz, Z., et al. (2012). "Dopamine modulation of latent inhibition in animal model of Schizophrenia." Amino Acids 42[[1](#_ENREF_1)]: 1524-1525.

Owens, S. E., et al. (2011). "Lack of association between autism and four heavy metal regulatory genes." Neurotoxicology 32(6): 769-775.

Owhadi, H. and A. Boulos (2008). "Bistable equilibrium points of mercury body burden." Journal of Biological Systems 16[[1](#_ENREF_1)]: 139-150.

Ozbayrak, K. R., et al. (1991). "LEFT OCCIPITAL HYPOPERFUSION IN A CASE WITH THE ASPERGER SYNDROME." Brain and Development 13(6): 454-456.

Pabello, N. G. and D. A. Lawrence (2006). "Neuroimmunotoxicology: Modulation of neuroimmune networks by toxicants." Clinical Neuroscience Research 6(1-2): 69-85.

Palmer, R. F., et al. (2006). "Environmental mercury release, special education rates, and autism disorder: An ecological study of Texas." Health and Place 12(2): 203-209.

Palmer, R. F., et al. (2009). "Proximity to point sources of environmental mercury release as a predictor of autism prevalence." Health Place 15[[1](#_ENREF_1)]: 18-24.

Palomo, T., et al. (2003). "Brain sites of movement disorder: genetic and environmental agents in neurodevelopmental perturbations." Neurotox Res 5(1-2): 1-26.

Pandit, J. J. and S. M. Yentis (2005). "All that glisters... How to assess the 'value' of a scientific paper." Anaesthesia 60[[1](#_ENREF_1)]: 373-383.

Pantaleoni, E. (2012). APPLYING GIS AND SPATIAL ANALYSIS TO STUDIES OF HEALTH IN CHILDREN WITH DISABILITIES. International Review of Research in Developmental Disabilities, Vol 42. R. M. Hodapp. San Diego, Elsevier Academic Press Inc. 42: 1-29.

Parcell, S. W. (2005). "Clinical pearls - Part I of the 39th Annual Meeting of the Academy of Environmental Medicine." Integrative Medicine 4(2): 42-45.

Parellada, M., et al. (2012). "Plasma antioxidant capacity is reduced in Asperger syndrome." J Psychiatr Res 46[[2](#_ENREF_2)]: 394-401.

Park, E. K., et al. (2007). "Evaluation of cytotoxicity attributed to thimerosal on murine and human kidney cells." Journal of Toxicology and Environmental Health-Part a-Current Issues 70(24): 2092-2095.

Parker, S. K., et al. (2004). "Thimerosal-containing vaccines and autistic spectrum disorder: A critical review of published original data." Pediatrics 114[[2](#_ENREF_2)]: 793-804.

Parker, W., et al. (2012). "A prescription for clinical immunology: the pills are available and ready for testing. A review." Current Medical Research and Opinion 28(7): 1193-1202.

Parry, P. I. and E. C. Levin (2012). "Pediatric Bipolar Disorder in an Era of "Mindless Psychiatry"." Journal of Trauma and Dissociation 13[[1](#_ENREF_1)]: 51-68.

Pasala, S., et al. (2013). "High-dose argatroban for heparin-induced thrombocytopenia in a child using a ventricular assist device." Annals of Thoracic Surgery 95[[2](#_ENREF_2)]: e57-e58.

Pasca, S. P., et al. (2010). "Paraoxonase 1 activities and polymorphisms in autism spectrum disorders." Journal of Cellular and Molecular Medicine 14[[2](#_ENREF_2)]: 600-607.

Patel, K. and L. T. Curtis (2007). "A comprehensive approach to treating autism and attention-deficit hyperactivity disorder: a prepilot study." J Altern Complement Med 13(10): 1091-1097.

Patrianakos-Hoobler, A. I., et al. (2009). "Risk Factors Affecting School Readiness in Premature Infants With Respiratory Distress Syndrome." Pediatrics 124[[1](#_ENREF_1)]: 258-267.

Paul, Y. and A. Parthasarathy (2005). "Preservatives in DPT vaccine." Indian Pediatrics 42(10): 1006-1007.

Paulson, J. A. (2007). "The state of children's health and the environment." Archives of Environmental & Occupational Health 62(2): 53-57.

Paulson, J. A. and L. Gordon (2011). "Clinical services in environmental pediatrics." Mount Sinai Journal of Medicine 78[[1](#_ENREF_1)]: 11-21.

Pavlovic, R. Y. and A. M. Pavlovic (2011). "Movies and mental illness stigma - A European cinematic perspective." European Psychiatry 26.

Pereira, J. A., et al. (2013). "Contagious comments: what was the online buzz about the 2011 Quebec measles outbreak?" PLoS One 8(5): e64072.

Persico, A. M. and S. Merelli (2014). "Environmental Factors in the Onset of Autism Spectrum Disorder." Curr Dev Disord Rep 1: 8–19.

Persico, A. M. and A. Pimenta (2003). "5'UTR human reelin gene variants associated with autistic disorder blunt reporter gene expression in neuronal and non - neuronal cell lines." Society for Neuroscience Abstract Viewer and Itinerary Planner 2003: Abstract No. 318.319.

Pessah, I. N. and P. J. Lein (2008). Evidence for Environmental Susceptibility in Autism What We Need to Know About Gene x Environment Interactions. Autism: Current Theories and Evidence. A. W. Zimmerman. Totowa, Humana Press Inc: 409-428.

Pfaender, S. and A. M. Grabrucker (2014). "Characterization of biometal profiles in neurological disorders." Metallomics 6(5): 960-977.

Phillips, J. (2008). "Mercury causes autism in one case." Journal of Orthomolecular Medicine 23[[1](#_ENREF_1)]: 218-219.

Pisalchaiyong, T., et al. (2005). "Comparison of the effectiveness of oral diazepam and midazolam for the sedation of autistic patients during dental treatment." Pediatr Dent 27[[2](#_ENREF_2)]: 198-206.

Pisano, W. (2006). "Keys to strengthening the supply of routinely recommended vaccines: View from industry." Clinical Infectious Diseases 42(SUPPL. 3): S111-S117.

Pisula, W. and E. Pisula (2014). "Autism prevalence and meat consumption -a hypothesis that needs to be tested." Med Hypotheses 83[[1](#_ENREF_1)]: 488-493.

Plaisted Grant, K. and G. Davis (2009). "Perception and apperception in autism: rejecting the inverse assumption." Philos Trans R Soc Lond B Biol Sci 364(1522): 1393-1398.

Polanska, K., et al. (2013). "Review of current evidence on the impact of pesticides, polychlorinated biphenyls and selected metals on attention deficit / hyperactivity disorder in children." Int J Occup Med Environ Health 26[[1](#_ENREF_1)]: 16-38.

Poliakov, E., et al. (2014). "Impairment of translation in neurons as a putative causative factor for autism." Biology Direct 9: 13.

Potera, C. (2014). "Echoes of autism? Inhaled ultrafine particles and brain changes in mice." Environ Health Perspect 122[[1](#_ENREF_1)]: A250.

Pragnya, B., et al. (2014). "Ameliorating effect of piperine on behavioral abnormalities and oxidative markers in sodium valproate induced autism in BALB/C mice." Behav Brain Res 270: 86-94.

Prandota, J. (2010). "Autism spectrum disorders may be due to cerebral toxoplasmosis associated with chronic neuroinflammation causing persistent hypercytokinemia that resulted in an increased lipid peroxidation, oxidative stress, and depressed metabolism of endogenous and exogenous substances." Research in Autism Spectrum Disorders 4(2): 119-155.

Prandota, J. (2010). "Neuropathological changes and clinical features of autism spectrum disorder participants are similar to that reported in congenital and chronic cerebral toxoplasmosis in humans and mice." Research in Autism Spectrum Disorders 4(2): 103-118.

Prandota, J. (2011). "Metabolic, immune, epigenetic, endocrine and phenotypic abnormalities found in individuals with autism spectrum disorders, Down syndrome and Alzheimer disease may be caused by congenital and/or acquired chronic cerebral toxoplasmosis." Research in Autism Spectrum Disorders 5[[1](#_ENREF_1)]: 14-59.

Pregenzer, G. (2014). "Interactive medical intelligence-the future is now." Journal of Maternal-Fetal and Neonatal Medicine 27: 408-409.

Press, C., et al. (2006). "Bottom-up, not top-down, modulation of imitation by human and robotic models." European Journal of Neuroscience 24[[1](#_ENREF_1)]: 2415-2419.

Pretorius, H. T., et al. (2009). "Metal toxicity determined by bioassay of urine porphyrins correlates with thyroid associated cognitive impairment and psychiatric disease." Thyroid 19: S61.

Price, J. (2013). "Induced pluripotent stem cells and in vitro models of neurodevelopmental disorders." European Neuropsychopharmacology 23: S1-S2.

Pringsheim, T. and D. Gorman (2012). "Second-generation antipsychotics for the treatment of disruptive behaviour disorders in children: a systematic review." Can J Psychiatry 57(12): 722-727.

Quaak, I., et al. (2013). "The dynamics of Autism Spectrum Disorders: How neurotoxic compounds and neurotransmitters interact." International Journal of Environmental Research and Public Health 10[[1](#_ENREF_1)]: 3384-3408.

Rahbar, M. H., et al. (2012). "The role of drinking water sources, consumption of vegetables and seafood in relation to blood arsenic concentrations of Jamaican children with and without Autism Spectrum Disorders." Sci Total Environ 433: 362-370.

Rahbar, M. H., et al. (2014). "Role of fruits, grains, and seafood consumption in blood cadmium concentrations of Jamaican children with and without Autism Spectrum Disorder." Research in Autism Spectrum Disorders 8[[1](#_ENREF_1)]: 1134-1145.

Rahbar, M. H., et al. (2014). "Blood manganese concentrations in Jamaican children with and without autism spectrum disorders." Environmental Health 13: 14.

Rahbar, M. H., et al. (2012). "Metabolic genes and blood lead concentrations in jamaican children with and without autism spectrum disorders." American Journal of Epidemiology 175: S126.

Rahbar, M. H., et al. (2013). "Seafood consumption and blood mercury concentrations in Jamaican children with and without autism spectrum disorders." Neurotox Res 23[[1](#_ENREF_1)]: 22-38.

Raiten, D. J., et al. (1984). "Vitamin and trace element assessment of autistic and learning disabled children." Nutrition and Behavior 2[[1](#_ENREF_1)]: 9-17.

Rajnarayanan, R., et al. (2009). Potential Role of Cation-Aquaporin Interactions in Autism. New York, Ieee.

Ramierz, E. and S. Luza (1967). "Dimethyl sulfoxide in the treatment of mental patients." Ann Ny Acad Sci 141((1)): 655-667.

Ramsay, M. (2010). "Genetic and epigenetic insights into fetal alcohol spectrum disorders." Genome Medicine 2[[1](#_ENREF_1)].

Rand, J. B., et al. (2009). "Neuroligin deficient mutants of C. elegans are hypersensitive to oxidative stress and some heavy metals." Society for Neuroscience Abstract Viewer and Itinerary Planner 39.

Rao, D. B., et al. (2011). "Histopathological evaluation of the nervous system in national toxicology program rodent studies: A modified approach." Toxicologic Pathology 39[[2](#_ENREF_2)]: 463-470.

Ratajczak, H. V. (2011). "Theoretical aspects of autism: causes--a review." J Immunotoxicol 8[[1](#_ENREF_1)]: 68-79.

Rauh, V. A., et al. (2006). "Impact of prenatal chlorpyrifos exposure on neurodevelopment in the first 3 years of life among inner-city children." Pediatrics 118(6): e1845-1859.

Reber, M. E. (2012). Epidemiology of autism. Cambridge, Cambridge Univ Press.

Reber, M. E. (2012). Etiology: essential autism. Cambridge, Cambridge Univ Press.

Reid, A. and S. Nihon (2011). "The effects of heart rate variability on sensorimotor rhythm: A pilot study." Journal of Neurotherapy 15[[1](#_ENREF_1)]: 419-420.

Rey, J. M., et al. (2008). "Complementary and alternative medicine (CAM) treatments and pediatric psychopharmacology." Journal of the American Academy of Child and Adolescent Psychiatry 47[[1](#_ENREF_1)]: 364-368.

Reynolds, A., et al. (2012). "Iron status in children with autism spectrum disorder." Pediatrics 130 Suppl 2: S154-159.

Rice, D. and S. Barone, Jr. (2000). "Critical periods of vulnerability for the developing nervous system: Evidence from humans and animal models." Environmental Health Perspectives 108(Supplement 3): 511-533.

Richendrfer, H., et al. (2012). "Developmental sub-chronic exposure to chlorpyrifos reduces anxiety-related behavior in zebrafish larvae." Neurotoxicol Teratol 34[[1](#_ENREF_1)]: 458-465.

Rimland, B. (2000). "The autism epidemic, vaccinations, and mercury." Journal of Nutritional and Environmental Medicine 10[[1](#_ENREF_1)]: 261-266.

Rinsky, R. A. (2002). "The Precautionary Principle: A message from the editor." Public Health Reports 117(6): 491-492.

Rizzo, S. J. S., et al. (2013). "Future viable models of psychiatry drug discovery in pharma." Journal of Biomolecular Screening 18(5): 509-521.

Roberts, A. L. (2014). "Perinatal Air Pollutant Exposures and Autism Spectrum Disorder in the Children of Nurses' Health Study II Participants (vol 121, pg 978, 2013)." Environmental Health Perspectives 122(6): A152-A152.

Roberts, J. R. and C. J. Karr (2012). "Pesticide exposure in children." Pediatrics 130(6): e1765-1788.

Roberts, W. and M. Harford (2002). "Immunization and children at risk for autism." Paediatrics and Child Health 7[[1](#_ENREF_1)]: 623-632.

Robinson, J. L. (2007). "Vaccine controversies in Canada." Canadian Pharmacists Journal 140(SUPPL. 2): S9-S10.

Robinson-Agramonte, M. A., et al. (2011). "DNA damage and immune-enviromental events in autism." Journal of Neurochemistry 118: 132.

Rode, D. (2006). "Are mercury amalgam fillings safe for children? An evaluation of recent research results." Altern Ther Health Med 12[[1](#_ENREF_1)]: 16-17.

Rogers, T. D., et al. (2013). "Is autism a disease of the cerebellum?: An integration of clinical and pre-clinical research." Frontiers in Systems Neuroscience(APR 2013).

Roman, G. and S. J. Rogers (2004). "Donepezil: A clinical review of current and emerging indications." Expert Opinion on Pharmacotherapy 5[[1](#_ENREF_1)]: 161-180.

Roman, G. C. (2007). "Autism: transient in utero hypothyroxinemia related to maternal flavonoid ingestion during pregnancy and to other environmental antithyroid agents." J Neurol Sci 262(1-2): 15-26.

Rommelse, N. (2014). "5.2 Are ASD symptoms more like 'gold dust' within the ADHD+ASD spectrum disorders?" Australian and New Zealand Journal of Psychiatry 48: 21-22.

Ronson, J. (2011). "The kids are not alright." New Scientist 210(2815): 44-47.

Rooney, J. P. K. (2008). "Mercury levels in newborns and infants after receipt of thimerosal-containing Vaccines." Pediatrics 122[[1](#_ENREF_1)]: 902.

Rosas, L. G. and B. Eskenazi (2008). "Pesticides and child neurodevelopment." Curr Opin Pediatr 20(2): 191-197.

Rose, S., et al. (2012). "Increased susceptibility to reactive oxygen species in autism lymphoblastoid cells is mediated by mitochondrial dysfunction." Society for Neuroscience Abstract Viewer and Itinerary Planner 42.

Rose, S., et al. (2012). "Evidence of oxidative damage and inflammation associated with low glutathione redox status in the autism brain." Translational Psychiatry 2: 8.

Rose, S., et al. (2008). "The frequency of polymorphisms affecting lead and mercury toxicity among children with autism." American Journal of Biochemistry and Biotechnology 4(2): 85-94.

Rose, S., et al. (2012). "Increased susceptibility to reactive oxygen species in autism lymphoblastoid cells is mediated by mitochondrial dysfunction." Mitochondrion (Kidlington) 12(5): 562.

Rosen, L. D. and D. Imus (2007). "Environmental Injustice: Children's Health Disparities and the Role of the Environment." Explore: The Journal of Science and Healing 3(5): 524-528.

Rosenblatt, N. L. (2007). "An economic impact assessment of lead exposure in the Commonwealth of Kentucky, USA: Making the case for statewide remediation." International Journal of Environment and Pollution 30(3-4): 443-456.

Ross, B., et al. (2013). "Anatomical impact of reelin haploinsufficiency and prenatal organophosphate pesticide exposure in mouse cerebellum." Society for Neuroscience Abstract Viewer and Itinerary Planner 43.

Rossignol, D. A. and R. E. Frye (2012). "Mitochondrial dysfunction in autism spectrum disorders: a systematic review and meta-analysis." Molecular Psychiatry 17[[2](#_ENREF_2)]: 290-314.

Rossignol, D. A. and R. E. Frye (2012). "A review of research trends in physiological abnormalities in autism spectrum disorders: immune dysregulation, inflammation, oxidative stress, mitochondrial dysfunction and environmental toxicant exposures." Molecular Psychiatry 17[[1](#_ENREF_1)]: 389-401.

Rossignol, D. A., et al. (2014). "Environmental toxicants and autism spectrum disorders: a systematic review." Transl Psychiatry 4: e360.

Rudkowski, Z. (2006). "The environmental risk with long-lasting sequellae for children - A "new paediatric morbidity"." Family Medicine and Primary Care Review 8(2): 459-460.

Russo, A. F. (2008). "Anti-metallothionein IgG and levels of metallothionein in autistic families." Swiss Med Wkly 138(5-6): 70-77.

Russo, A. J. and R. Gilbride (2003). "Structural difference of MTF-1 in some autistic individuals." Journal of the Pennsylvania Academy of Science 77[[1](#_ENREF_1)]: 3-6.

Rutter, M. (1999). "The Emanuel Miller Memorial Lecture 1998. Autism: two-way interplay between research and clinical work." J Child Psychol Psychiatry 40(2): 169-188.

Rutter, M. (2005). "Aetiology of autism: Findings and questions." Journal of Intellectual Disability Research 49[[1](#_ENREF_1)]: 231-238.

Ryan, P. B., et al. (2007). "Using biomarkers to inform cumulative risk assessment." Environmental Health Perspectives 115(5): 833-840.

Ryu, Y. H., et al. (1999). "Perfusion impairments in infantile autism on technetium-99m ethyl cysteinate dimer brain single-photon emission tomography: comparison with findings on magnetic resonance imaging." European Journal of Nuclear Medicine 26[[2](#_ENREF_2)]: 253-259.

Rzhetsky, A., et al. (2014). "Environmental and State-Level Regulatory Factors Affect the Incidence of Autism and Intellectual Disability." Plos Computational Biology 10[[2](#_ENREF_2)]: 11.

Sadamatsu, M., et al. (2006). "Review of animal models for autism: Implication of thyroid hormone." Congenital Anomalies 46[[1](#_ENREF_1)]: 1-9.

Saito, K., et al. (2013). "Removable orthodontic appliance with nickel-titanium spring to reposition the upper incisors in an autistic patient." Spec Care Dentist 33[[1](#_ENREF_1)]: 35-39.

Sajdel-Sulkowska, E. M., et al. (2008). "Oxidative stress in autism: Elevated cerebellar 3-nitrotyrosine levels." American Journal of Biochemistry and Biotechnology 4(2): 73-84.

Sakul, A. S. (2008). "Heavy Metal Detoxification and Chelation Therapy for AntiAging." Turkiye Klinikleri Tip Bilimleri Dergisi 28(6): S236-S244.

Salas, J. and C. A. Smith (2008). "CHED 199-Search for mercury near a coal burning power plant." Abstracts of Papers American Chemical Society 236: 199-CHED.

Salhia, H. O., et al. (2014). "Systemic review of the epidemiology of autism in Arab gulf countries." Neurosciences 19[[1](#_ENREF_1)]: 291-296.

Samsel, A. and S. Seneff (2013). "Glyphosate's Suppression of Cytochrome P450 Enzymes and Amino Acid Biosynthesis by the Gut Microbiome: Pathways to Modern Diseases." Entropy 15[[1](#_ENREF_1)]: 1416-1463.

Samuni, Y., et al. (2013). "The chemistry and biological activities of N-acetylcysteine." Biochimica et Biophysica Acta - General Subjects 1830[[1](#_ENREF_1)]: 4117-4129.

San Pedro, E. C., et al. (1997). "Global rCBF SPECT abnormalities correlate with the severity of autistic disorder." Journal of Nuclear Medicine 38(5 SUPPL.): 13P.

San Pedro, E. C., et al. (1997). "Reduction in temporal lobe rCBF is associated with childhood autistic disorder and their lack of social relatedness." Journal of Investigative Medicine 45[[1](#_ENREF_1)]: 14A.

Sandhya, T., et al. (2012). "Bacopa monniera (L.) Wettst ameliorates behavioral alterations and oxidative markers in sodium valproate induced autism in rats." Neurochem Res 37(5): 1121-1131.

Sanua, V. D. (1981). "Cultural changes and psychopathology in children: With special reference to infantile autism." Acta Paedopsychiatrica 47[[2](#_ENREF_2)]: 133-142.

Satomoto, M., et al. (2009). "Neonatal exposure to sevoflurane induces abnormal social behaviors and deficits in fear conditioning in mice." Anesthesiology 110[[2](#_ENREF_2)]: 628-637.

Schaevitz, L. R., et al. (2010). "Cognitive and social functions and growth factors in a mouse model of Rett syndrome." Physiology & Behavior 100(3, Sp. Iss. SI): 255-263.

Schantz, S. (2008). "An overview of the centers for children's environmental health and disease prevention: Research, translation and outreach." Neurotoxicology and Teratology 30[[2](#_ENREF_2)]: 245.

Schechtman, M. A. (2007). "Scientifically unsupported therapies in the treatment of young children with autism spectrum disorders." Pediatric Annals 36[[1](#_ENREF_1)]: 497-505.

Schendel, D. E., et al. (2012). "The Study to Explore Early Development (SEED): A Multisite Epidemiologic Study of Autism by the Centers for Autism and Developmental Disabilities Research and Epidemiology (CADDRE) Network." Journal of Autism and Developmental Disorders 42(10): 2121-2140.

Schmeits, P. C. J., et al. (2009). "Investigating unexpected INRs: In search of the culprit Adherence, interactions, genetics, and superwarfarin." Netherlands Journal of Medicine 67(2): 76-78.

Schmid, C. and J. S. Rotenberg (2005). "Neurodevelopmental toxicology." Neurologic Clinics 23(2): 321-+.

Schmid, E. F., et al. (2007). "Communicating the risks and benefits of medicines." Drug Discovery Today 12(9-10): 355-364.

Schneider, T., et al. (2008). "Gender-specific behavioral and immunological alterations in an animal model of autism induced by prenatal exposure to valproic acid." Psychoneuroendocrinology 33(6): 728-740.

Schubert, C. (2010). "Pandemic blows lid off laws limiting mercury in vaccines." Nature Medicine 16[[1](#_ENREF_1)]: 9.

Schultz, S. T. (2010). "Does thimerosal or other mercury exposure increase the risk for autism? A review of current literature." Acta Neurobiol Exp (Wars) 70(2): 187-195.

Schwartz, D. A., et al. (2004). "Environmental genomics: a key to understanding biology, pathophysiology and disease." Human Molecular Genetics 13: R217-R224.

Schwartzer, J. J., et al. (2013). "Using mouse models of autism spectrum disorders to study the neurotoxicology of gene-environment interactions." Neurotoxicology and Teratology 36: 17-35.

Scumpia, P. O., et al. (2014). "Alpha-lipoic acid effects on brain glial functions accompanying double-stranded RNA antiviral and inflammatory signaling." Neurochemistry International 64: 55-63.

Sealey, L. and O. Bagasra (2014). "The male gender bias in autism may be due to preferential depletions of oxytocin and arginine-vasopressin receptors positive neurons exposed to certain fragrances during fetal development." FASEB Journal 28[[1](#_ENREF_1)].

Sears, M. E. (2013). "Chelation: Harnessing and Enhancing Heavy Metal Detoxification-A Review." Scientific World Journal: 219840.

Secor, J. D., et al. (2011). "Novel Lipid-Soluble Thiol-Redox Antioxidant and Heavy Metal Chelator, N,N '-bis(2-Mercaptoethyl)Isophthalamide (NBMI) and Phospholipase D-Specific Inhibitor, 5-Fluoro-2-Indolyl Des-Chlorohalopemide (FIPI) Attenuate Mercury-Induced Lipid Signaling Leading to Protection Against Cytotoxicity in Aortic Endothelial Cells." International Journal of Toxicology 30(6): 619-638.

Seggie, J. (2013). "Editor's choice." South African Medical Journal 103(5): 271-272.

Segura Benedicto, A. (2012). "[The putative link between the MMR vaccine and autism and refusal to vaccinate]." Gac Sanit 26[[1](#_ENREF_1)]: 366-371.

Seibel, M. M. (2006). "The environment: Its risks to conception and pregnancy." Sexuality, Reproduction and Menopause 4[[1](#_ENREF_1)]: 1-2.

Seitler, B. N. (2010). "New information that people in high places do not want us to know about autism." Ethical Human Psychology and Psychiatry 12(2): 144-157.

Sempere, A., et al. (2010). "Study of inborn errors of metabolism in urine from patients with unexplained mental retardation." J Inherit Metab Dis 33[[1](#_ENREF_1)]: 1-7.

Seneff, S., et al. (2012). "Might cholesterol sulfate deficiency contribute to the development of autistic spectrum disorder?" Med Hypotheses 78(2): 213-217.

Seneff, S., et al. (2012). "Empirical Data Confirm Autism Symptoms Related to Aluminum and Acetaminophen Exposure." Entropy 14[[1](#_ENREF_1)]: 2227-2253.

Seneff, S., et al. (2012). "Is Cholesterol Sulfate Deficiency a Common Factor in Preeclampsia, Autism, and Pernicious Anemia?" Entropy 14[[1](#_ENREF_1)]: 2265-2290.

Seneff, S., et al. (2013). "Is Encephalopathy a Mechanism to Renew Sulfate in Autism?" Entropy 15[[1](#_ENREF_1)]: 372-406.

Sener, E. F., et al. (2013). "The association of TNF-(alpha) and NOS3 gene expressions in autism." Klinik Psikofarmakoloji Bulteni 23: S176.

Serajee, F. J., et al. (2004). "Polymorphisms in xenobiotic metabolism genes and autism." J Child Neurol 19(6): 413-417.

Serajee, F. J., et al. (2003). "Investigation of 40 positional and functional candidate genes for autism." American Journal of Medical Genetics 122B[[1](#_ENREF_1)]: 117-118.

Seshardri, K., et al. (2004). PRELIMINARY RESULTS OF HOME ENVIRONMENTAL ASSESSMENT AND CHEMICAL EXPOSURE, AND BODY BURDENS OF CHILDREN WITH AUTISM SPECTRUM DISORDER OR PDD-NOS (ASD). The Sixteenth Conference of the International Society for Environmental Epidemiology (ISEE), Lippincott Williams & Wilkins.

Sezgin, C., et al. (2010). "Comparison of blood toxic and plasma essential elements of the autistic Turkish infants." FEBS Journal 277: 88.

Sezgin, C., et al. (2010). "Excretion by urine of toxic and essential elements of the autistic Turkish infants." FEBS Journal 277: 89.

Shalat, S. L., et al. (2004). "NJ autism study: The role of behavior in ingestion of environmental toxins." Neurotoxicology (Amsterdam) 25[[1](#_ENREF_1)]: 683.

Shandley, K. and D. W. Austin (2011). "Ancestry of pink disease (infantile acrodynia) identified as a risk factor for autism spectrum disorders." J Toxicol Environ Health A 74(18): 1185-1194.

Shandley, K., et al. (2014). "Are urinary porphyrins a valid diagnostic biomarker of autism spectrum disorder?" Autism Research.

Shannon, M. and J. W. Graef (1996). "Lead intoxication in children with pervasive developmental disorders." Journal of Toxicology Clinical Toxicology 34(2): 177-181.

Shannon, M., et al. (2003). "Children's environmental health: one year in a pediatric environmental health specialty unit." Ambul Pediatr 3[[1](#_ENREF_1)]: 53-56.

Sharma, H. S., et al. (2013). "Novel therapeutic strategies using nanodrug delivery, stem cells and combination therapy for CNS trauma and neurodegenerative disorders." Expert Review of Neurotherapeutics 13(10): 1085-1088.

Sharma, H. S. and A. Sharma (2014). "The American Association for the Advancement of Science (AAAS), 180th Annual Meeting Chicago, IL, USA Feb 13-17, 2014 "Meeting Global Challenges: Discoveries & Innovation"." CNS & Neurological Disorders-Drug Targets 13[[1](#_ENREF_1)]: 553-555.

Sharp, S. I., et al. (2009). "Genetics of attention-deficit hyperactivity disorder (ADHD)." Neuropharmacology 57(7-8): 590-600.

Shattock, P. E. G., et al. (2003). Is there an increasing incidence of autism? Evidence and possible explanations. Hamilton, B C Decker Inc (Canada).

Shearer, T. R., et al. (1982). "Minerals in the hair and nutrient intake of autistic children." J Autism Dev Disord 12[[1](#_ENREF_1)]: 25-34.

Shelton, J. F. and I. Hertz-Picciotto (2010). "EFFECT OF MATERNAL OCCUPATIONAL PESTICIDE EXPOSURE ON COGNITIVE AND ADAPTIVE FUNCTION IN THE CHARGE STUDY." American Journal of Epidemiology 171(Suppl. 11): S99.

Shelton, J. F., et al. (2012). "Tipping the balance of autism risk: potential mechanisms linking pesticides and autism." Environ Health Perspect 120(7): 944-951.

Sheng, L., et al. (2010). "Prenatal polycyclic aromatic hydrocarbon exposure leads to behavioral deficits and downregulation of receptor tyrosine kinase, MET." Toxicol Sci 118(2): 625-634.

Sheth, J. (2014). "Editorial." Molecular Cytogenetics 7.

Shimura, A., et al. (1997). "Dental-management conditions of risky and handicapped children at the Department of Pediatric Dentistry, Tokyo Dental College." Shikwa Gakuho 97(5): 545-551.

Shwed, U. and P. S. Bearman (2010). "The Temporal Structure of Scientific Consensus Formation." American Sociological Review 75(6): 817-840.

Siegel, B. V., Jr. and M. S. Buchsbaum (1993). "Glucose metabolic correlates of attentional performance in adults with a history of infantile autism, schizophrenics, and controls." Society for Neuroscience Abstracts 19(1-3): 1224.

Siegrist, C. A. (2007). "Mechanisms Underlying Adverse Reactions to Vaccines." Journal of Comparative Pathology 137(SUPPL. 1): S46-S50.

Silbergeld, E. K. (2008). "Mercury, vaccines, and autism, revisited." American Journal of Public Health 98[[1](#_ENREF_1)]: 1350.

Silva, M. H. and S. L. Beauvais (2010). "Human health risk assessment of endosulfan. I: Toxicology and hazard identification." Regulatory Toxicology and Pharmacology 56[[1](#_ENREF_1)]: 4-17.

Silva, M. H. and D. Gammon (2009). "An assessment of the developmental, reproductive, and neurotoxicity of endosulfan." Birth Defects Res B Dev Reprod Toxicol 86[[1](#_ENREF_1)]: 1-28.

Simoni, R. E., et al. (2007). "Uric acid changes in urine and plasma: An effective tool in screening for purine inborn errors of metabolism and other pathological conditions." Journal of Inherited Metabolic Disease 30[[2](#_ENREF_2)]: 295-309.

Simpson, E. A., et al. (2014). "Inhaled oxytocin increases positive social behaviors in newborn macaques." Proceedings of the National Academy of Sciences of the United States of America 111[[1](#_ENREF_1)]: 6922-6927.

Simpson, K. L., et al. (2011). "Perinatal antidepressant exposure alters cortical network function in rodents." Proceedings of the National Academy of Sciences of the United States of America 108(45): 18465-18470.

Singh, V. K. (2009). "Phenotypic expression of autoimmune autistic disorder (AAD): a major subset of autism." Ann Clin Psychiatry 21[[2](#_ENREF_2)]: 148-161.

Singh, V. K. and J. Hanson (2006). "Assessment of metallothionein and antibodies to metallothionein in normal and autistic children having exposure to vaccine-derived thimerosal." Pediatr Allergy Immunol 17[[1](#_ENREF_1)]: 291-296.

Singh, V. K. and W. H. Rivas (2004). "Detection of antinuclear and antilaminin antibodies in autistic children who received thimerosal-containing vaccines." Journal of Biomedical Science 11(5): 607-610.

Siniscalco, D. and N. Antonucci (2013). "Involvement of dietary bioactive proteins and peptides in autism spectrum disorders." Curr Protein Pept Sci 14[[1](#_ENREF_1)]: 674-679.

Siniscalco, D. and N. Antonucci (2013). "Possible use of Trichuris suis ova in autism spectrum disorders therapy." Med Hypotheses 81[[1](#_ENREF_1)]: 1-4.

Siniscalco, D., et al. (2013). "Therapeutic role of hematopoietic stem cells in autism spectrum disorder-related inflammation." Front Immunol 4: 140.

Sipp, D. (2011). "Global Challenges in Stem Cell Research and the Many Roads Ahead." Neuron 70[[1](#_ENREF_1)]: 573-576.

Sirigu, A. (2011). "How oxytocin affects the human brain and behavior." Hormone Research in Paediatrics 76: 7.

Siva, N. (2012). "Thiomersal vaccines debate continues ahead of un meeting." The Lancet 379(9834): 2328.

Skripka-Serry, J. (2013). "The great neuro-pipeline 'brain drain' (and why Big Pharma hasn't given up on CNS disorders)." Drug Discovery World 14[[1](#_ENREF_1)]: 9-16.

Slomski, A. (2012). "Chronic mental health issues in children now loom larger than physical problems." JAMA - Journal of the American Medical Association 308[[2](#_ENREF_2)]: 223-225.

Smith, D. and B. J. Strupp (2013). "The scientific basis for chelation: animal studies and lead chelation." J Med Toxicol 9[[1](#_ENREF_1)]: 326-338.

Smith, J., et al. (2011). "Determination of plasma DMPS concentration and urine mercury excretion after dermal application of null TD-DMPS null." Clinical Toxicology 49(6): 543.

Soden, S. E., et al. (2008). "Authors' reply to letter regarding the original article "24-hour provoked excretion test for heavy metals in children with autism and typically developing controls, a pilot study"." Clinical Toxicology 46(10): 1098-1098.

Soden, S. E., et al. (2006). "Provoked urinary excretion testing for heavy metals in children with autism." Clinical Toxicology 44(5): 783-783.

Soden, S. E., et al. (2007). "24-hour provoked urine excretion test for heavy metals in children with autism and typically developing controls, a pilot study." Clin Toxicol (Phila) 45(5): 476-481.

Sogut, S., et al. (2003). "Changes in nitric oxide levels and antioxidant enzyme activities may have a role in the pathophysiological mechanisms involved in autism." Clin Chim Acta 331(1-2): 111-117.

Sohler, A., et al. (1977). "Blood levels in psychiatric outpatients reduced by zinc and vitamin C." Journal of Orthomolecular Psychiatry 6[[2](#_ENREF_2)]: 272-276.

Spooren, W. (2014). "EU-AIMS: A trans-dimensional approach for enhancing our understanding of autism spectrum disorders." European Neuropsychopharmacology 24: S130-S131.

Srivastava, C. (2013). "Autism spectrum disorders: Increasing prevalence and unmet need." Journal of Indian Association for Child and Adolescent Mental Health 9[[2](#_ENREF_2)]: 52-61.

Stamou, M., et al. (2013). "Neuronal connectivity as a convergent target of gene x environment interactions that confer risk for Autism Spectrum Disorders." Neurotoxicology and Teratology 36: 3-16.

Stamova, B., et al. (2011). "Correlations between gene expression and mercury levels in blood of boys with and without autism." Neurotox Res 19[[1](#_ENREF_1)]: 31-48.

Stangle, D. E., et al. (2007). "Succimer chelation improves learning, attention, and arousal regulation in lead-exposed rats but produces lasting cognitive impairment in the absence of lead exposure." Environmental Health Perspectives 115(2): 201-209.

Stanley, F. (2002). "From Susser's causal paradigms to social justice in Australia?" International Journal of Epidemiology 31[[1](#_ENREF_1)]: 40-45.

Stein, T. P., et al. (2013). "Autism and Phthalate Metabolite Glucuronidation." Journal of Autism and Developmental Disorders 43[[1](#_ENREF_1)]: 2677-2685.

Steinborn, M. and T. J. Knapp (1982). "Teaching an autistic child pedestrian skills." J Behav Ther Exp Psychiatry 13[[1](#_ENREF_1)]: 347-351.

Stephenson, J. (2002). "Will the current measles vaccines ever eradicate measles?" Expert Rev Vaccines 1[[2](#_ENREF_2)]: 355-362.

Stephenson, P. S. (1975). "The hyperkinetic child: some misleading assumptions." Canadian Medical Association Journal 113[[1](#_ENREF_1)]: 764-769.

Stewart, A. M. "When vaccine injury claims go to court." N Engl J Med.

St-Hilaire, S., et al. (2012). "An ecological study on childhood autism." International Journal of Health Geographics 11: 8.

Stoccoro, A., et al. (2013). "Epigenetic effects of nano-sized materials." Toxicology 313[[1](#_ENREF_1)]: 3-14.

Stoeckli, E. T. (2012). "What does the developing brain tell us about neural diseases?" European Journal of Neuroscience 35(12): 1811-1817.

Stokstad, E. (2003). "Vaccine-autism link dealt blow." Science 301(5639): 1454-1455.

Stoner, A. M., et al. (2013). "Ambient Air Toxics and Asthma Prevalence among a Representative Sample of US Kindergarten-Age Children." Plos One 8[[1](#_ENREF_1)]: 11.

Stoyanova-Beninska, V. V., et al. (2011). "The EU paediatric regulation: Effects on paediatric psychopharmacology in Europe." European Neuropsychopharmacology 21[[1](#_ENREF_1)]: 565-570.

Suaifan, G. A. R. Y., et al. (2014). "Ritodrine inhibits neuronal nitric oxide synthase, a potential link between tocolysis and autism." Medicinal Chemistry Research.

Sugamata, M., et al. (2006). "Maternal exposure to diesel exhaust leads to pathological similarity to autism in newborns." Journal of Health Science 52[[1](#_ENREF_1)]: 486-488.

Sugamata, M., et al. (2006). Maternal diesel exhaust exposure induces pathological findings similar to autism in newborn murine brains. 40128 Bologna, Medimond S R L.

Sullivan, K. M. (2008). "The interaction of agricultural pesticides and marginal iodine nutrition status as a cause of autism spectrum disorders." Environ Health Perspect 116[[1](#_ENREF_1)]: A155.

Sun, L. S. (2011). "Labor analgesia and the developing human brain." Anesthesia and Analgesia 112(6): 1265-1267.

Sundaram, S. K., et al. (2005). "Positron emission tomography methods with potential for increased understanding of mental retardation and developmental disabilities." Ment Retard Dev Disabil Res Rev 11[[1](#_ENREF_1)]: 325-330.

Sundaramoorthy, R., et al. (2013). "Genetic damage analysis of Autism Spectrum Disorder (ASD) patients of Tamilnadu, South India using Cytokinesis-block micronucleus cytome (CBMN cyt) assay." Research Journal of Biotechnology 8[[1](#_ENREF_1)]: 78-86.

Swaminathan, M., et al. (2013). "Nitric Oxide Mediated Colonic Motility Is Altered in the Neuroligin-3 R451c Mouse Model of Autism." Gastroenterology 144(5): S543-S543.

Sweeten, T. L., et al. (2004). "High nitric oxide production in autistic disorder: a possible role for interferon-gamma." Biol Psychiatry 55[[1](#_ENREF_1)]: 434-437.

Swiergiel, A. H., et al. (2010). "In silico analysis of expression of orphan G protein-coupled receptor 155 during inflammation-implications for autism." European Neuropsychopharmacology 20: S219-S220.

Swift, I., et al. (1999). "Pica and trace metal deficiencies in adults with developmental disabilities." British Journal of Developmental Disabilities 45(2): 111-117.

Szpir, M. (2006). "New thinking on neurodevelopment." Environmental Health Perspectives 114(2): A100-A107.

Tachibana, M., et al. (2012). "Long-term administration of intranasal oxytocin to early adolescents with autistic spectrum disorder." Developmental Medicine and Child Neurology 54: 66.

Takagi, J., et al. (1998). "Special considerations in dental treatment of the handicapped under general anesthesia (mainly day-case anesthesia) at Osaka University Dental Hospital." Journal of Japanese Dental Society of Anesthesiology 26[[1](#_ENREF_1)]: 56-64.

Takahashi, K., et al. (2001). "Dysfunction of the dopaminergic system in pervasive developmental disorder: A pet study with drug treatment." Society for Neuroscience Abstracts 27[[1](#_ENREF_1)]: 1195.

Takahashi, K., et al. (2002). "DYSFUNCTION OF DOPAMINERGIC SYSTEM IN INFANTILE AUTISM: LONGITUDINAL PET STUDY WITH TETRAHYDROBIOPTERIN TREATMENT." Society for Neuroscience Abstract Viewer and Itinerary Planner 2002: Abstract No. 207.201.

Talbott, E., et al. (2014). The Association of National Air Toxics Assessment Exposures and the Risk of Childhood Autism Spectrum Disorder: A Case Control Study. AAAR 33rd Annual Conference (American Association for Aerosol Research), Orlando, Florida, USA.

Tanaka-Nakadate, S., et al. (2000). "Ontogenetic change of GTP-cyclohydrolase I expression in the rat brain." Neuroscience Research Supplement(24): S56.

Tayebati, S. K., et al. (2013). "Intranasal drug delivery to the central nervous system: Present status and future outlook." Current Pharmaceutical Design 19[[2](#_ENREF_2)]: 510-526.

Taylor, E. and J. Warner Rogers (2005). "Practitioner review: Early adversity and developmental disorders." Journal of Child Psychology and Psychiatry and Allied Disciplines 46(5): 451-467.

Taylor, L. E., et al. (2014). "Vaccines are not associated with autism: An evidence-based meta-analysis of case-control and cohort studies." Vaccine 32(29): 3623-3629.

Testa, C., et al. (2012). "Di-(2-ethylhexyl) phthalate and autism spectrum disorders." ASN Neuro 4[[1](#_ENREF_1)]: 223-229.

Thapar, A., et al. (2013). "Practitioner Review: What have we learnt about the causes of ADHD?" Journal of Child Psychology and Psychiatry 54[[1](#_ENREF_1)]: 3-16.

Theoharides, T. C. (2012). "Activation of mast cells and neuroglia leads to brain inflammation inhibited by luteolin-implications for treatment of alzheimer's and autism." Annals of Nutrition and Metabolism 61[[1](#_ENREF_1)]: 324-325.

Theoharides, T. C., et al. (2009). "Mercury induces histamine and vascular endothelial growth factor (VEGF) release from human mast cells." Annals of Allergy, Asthma and Immunology 103(5): A75.

Thirtamara Rajamani, K., et al. (2013). "Prenatal and early-life exposure to high-level diesel exhaust particles leads to increased locomotor activity and repetitive behaviors in mice." Autism Res 6[[1](#_ENREF_1)]: 248-257.

Thirumala, S., et al. (2013). "Manufacturing and banking of mesenchymal stem cells." Expert Opinion on Biological Therapy 13(5): 673-691.

Thomas Curtis, J., et al. "Chronic inorganic mercury exposure induces sex-specific changes in central TNF&alpha; expression: importance in autism?" Neurosci Lett.

Thomas Curtis, J., et al. (2011). "Chronic inorganic mercury exposure induces sex-specific changes in central TNFalpha expression: importance in autism?" Neurosci Lett 504[[1](#_ENREF_1)]: 40-44.

Thompson, L. and L. M. Kaufman (2003). "The visually impaired child." Pediatric Clinics of North America 50[[1](#_ENREF_1)]: 225-239.

Thony, B., et al. (2000). "Tetrahydrobiopterin biosynthesis, regeneration and functions." Biochemical Journal 347: 1-16.

Thrasher, J. D. and K. H. Kilburn (2001). "Embryo toxicity and teratogenicity of formaldehyde." Archives of Environmental Health 56[[1](#_ENREF_1)]: 300-311.

Tian, Y., et al. (2011). "Correlations of gene expression with blood lead levels in children with autism compared to typically developing controls." Neurotox Res 19[[1](#_ENREF_1)]: 1-13.

Toal, G. and D. Nathan (2014). "A novel approach to NICE audit?" Archives of Disease in Childhood 99: A60.

Tobiasova, Z., et al. (2011). "Risperidone-Related Improvement of Irritability in Children with Autism Is not Associated with Changes in Serum of Epidermal Growth Factor and Interleukin-13." Journal of Child and Adolescent Psychopharmacology 21(6): 555-564.

Toe, T. T., et al. (2010). Emotion Indexing using Hidden Markov Expert Rule Model (HMER) for autism children. New York, Ieee.

Tonge, B., et al. (2011). "Testing the 'Extreme male brain theory'." Journal of Intellectual Disability Research 55(10): 954.

Topal, T. and A. Korkmaz (2008). "Hyperbaric oxygen therapy: Medical education." Turkiye Klinikleri Tip Bilimleri Dergisi 28(2): 206-216.

Toplak, N. and T. Avcin (2009). Influenza and autoimmunity. 1173: 619-626.

Torsdottir, G., et al. (2005). "Ceruloplasmin, superoxide dismutase and copper in autistic patients." Basic Clin Pharmacol Toxicol 96(2): 146-148.

Tostes, M. H., et al. (2012). "Altered neurotrophin, neuropeptide, cytokines and nitric oxide levels in autism." Pharmacopsychiatry 45(6): 241-243.

Toth, E. A., et al. (2000). "The crystal structure of adenylosuccinate lyase from Pyrobaculum aerophilum reveals an intracellular protein with three disulfide bonds." J Mol Biol 301(2): 433-450.

Tracey, M. P. and K. Koide (2014). "Development of a Sustainable Enrichment Strategy for Quantification of Mercury Ions in Complex Samples at the Sub-Parts per Billion Level." Industrial & Engineering Chemistry Research 53(38): 14565-14570.

Trainor, B. C., et al. (2007). "Impaired nitric oxide synthase signaling dissociates social investigation and aggression." Behavioral Neuroscience 121(2): 362-369.

Trasande, L. and P. J. Landrigan (2004). "The National Children's Study: A critical national investment." Environmental Health Perspectives 112[[1](#_ENREF_1)]: A789-A790.

Trasande, L. and Y. Liu (2011). "Reducing the staggering costs of environmental disease in children, estimated at $76.6 billion in 2008." Health Aff (Millwood) 30(5): 863-870.

Treem, W. R. (2006). "Liver transplantation for non-hepatotoxic inborn errors of metabolism." Current Gastroenterology Reports 8[[2](#_ENREF_2)]: 215-223.

Trentini, J. F., III, et al. (2009). "Prenatal exposure to low levels of carbon monoxide impairs interneuron migration into the neocortex." Society for Neuroscience Abstract Viewer and Itinerary Planner 39.

Troili, G. M., et al. (2013). "Investigation on a group of autistic children: risk factors and medical social considerations." Clinica Terapeutica 164[[1](#_ENREF_1)]: E273-E278.

Tsai, S. J. (2006). "Deltamethrin, a pyrethroid insecticide, could be a potential antidepressant agent." Medical Hypotheses 66[[2](#_ENREF_2)]: 605-608.

Tsjirkoff, A. M. (1950). "Psychopathological syndromes in chronic mercury poisoning." Giornale di psichiatria e di neuropatologia 19[[2](#_ENREF_2)]: 45-49.

Tsukahara, H. (2007). "Biomarkers for oxidative stress: Clinical application in pediatric medicine." Current Medicinal Chemistry 14[[2](#_ENREF_2)]: 339-351.

Tuormaa, T. E. (2000). "Chromium, selenium, copper and other trace minerals in health and reproduction." Journal of Orthomolecular Medicine 15[[2](#_ENREF_2)]: 145-156.

Turlejski, K. (2010). "Focus on autism." Acta Neurobiol Exp (Wars) 70(2): 117-118.

Turner, E. H. (2013). "Publication bias, with a focus on psychiatry: causes and solutions." CNS Drugs 27(6): 457-468.

Tyszka-Czochara, M., et al. (2014). "THE ROLE OF ZINC IN THE PATHOGENESIS AND TREATMENT OF CENTRAL NERVOUS SYSTEM (CNS) DISEASES. IMPLICATIONS OF ZINC HOMEOSTASIS FOR PROPER CNS FUNCTION." Acta Poloniae Pharmaceutica 71[[2](#_ENREF_2)]: 369-377.

Ulay, H. T. and A. Ertugrul (2009). "Neuroimaging Findings in Autism: A Brief Review." Turk Psikiyatri Dergisi 20(2): 164-174.

Ustun, O. and G. Ongen (2012). "Production and separation of dipeptidyl peptidase IV from Lactococcus lactis: scale up for industrial production." Bioprocess and Biosystems Engineering 35[[1](#_ENREF_1)]: 1417-1427.

Vaman Rao Diwan, P. and R. Kakalij (2014). "Erythropoietin ameliorates neurobehavioral aberrations against valproate induced autism in mice." Basic and Clinical Pharmacology and Toxicology 115: 191.

van Balkom, I. D., et al. (2012). "Paternal age and risk of autism in an ethnically diverse, non-industrialized setting: Aruba." PLoS One 7[[1](#_ENREF_1)]: e45090.

van den Hazel, P., et al. (2006). "Today's epidemics in children: possible relations to environmental pollution and suggested preventive measures." Acta Paediatr Suppl 95(453): 18-25.

Van Der Linde, A. A. A., et al. (2008). "Stevens-Johnson syndrome in a child with chronic mercury exposure and 2,3-dimercaptopropane-1-sulfonate (DMPS) therapy." Clinical Toxicology 46(5): 479-481.

van Kamp, I. and H. Davies (2013). "Noise and health in vulnerable groups: A review." Noise & Health 15(64): 153-159.

Van Meter, K. C., et al. (2010). "Geographic distribution of autism in California: a retrospective birth cohort analysis." Autism Res 3[[1](#_ENREF_1)]: 19-29.

Van Wieren, T. A., et al. (2012). "Autism spectrum disorders and intellectual disabilities: A comparison of ADA Title i workplace discrimination allegations." Journal of Vocational Rehabilitation 36[[2](#_ENREF_2)]: 159-169.

Van Wieren, T. A., et al. (2008). "Workplace discrimination and autism spectrum disorders: The National EEOC Americans with Disabilities Act Research project." Work 31[[2](#_ENREF_2)]: 299-308.

Vargas Marcos, F. (2005). "Environmental pollution as determinant factor of health." Revista Espanola de Salud Publica 79(2): 117-127.

Vatanoglu-Lutz, E. E., et al. (2014). "Medicine in stamps: History of autism spectrum disorder (ASD) through philately." Journal of Neurological Sciences 31(2): 426-434.

Ventrice, P., et al. (2013). "Phthalates: European regulation, chemistry, pharmacokinetic and related toxicity." Environ Toxicol Pharmacol 36[[1](#_ENREF_1)]: 88-96.

Vernekar, D. (2013). Is air pollution a plausible candidate for prenatal exposure in autism spectrum disorder (ASD)? : a systematic review. Masters of Public Health, University of Hong Kong.

Verstraeten, T. "Thimerosal, the Centers for Disease Control and Prevention, and GlaxoSmithKline." Pediatrics.

Villagonzalo, K. A., et al. (2010). "Oxidative pathways as a drug target for the treatment of autism." Expert Opinion on Therapeutic Targets 14(12): 1301-1310.

Vitiello, B. (2001). "Psychopharmacology for young children: Clinical needs and research opportunities." Pediatrics 108[[1](#_ENREF_1)]: 983-989.

Vitiello, B. (2008). "Recent developments and strategies in pediatric pharmacology research in the USA." Child and Adolescent Psychiatry and Mental Health 2.

Vitiello, B., et al. (2004). "The interface between publicly funded and industry-funded research in pediatric psychopharmacology: Opportunities for integration and collaboration." Biological Psychiatry 56[[1](#_ENREF_1)]: 3-9.

Vitiello, B. and A. Wagner (2004). "Government initiatives in autism clinical trials." CNS Spectr 9[[1](#_ENREF_1)]: 66-70.

Voelker, R. (2010). "FDA warning targets OTC chelation products." JAMA - Journal of the American Medical Association 304[[1](#_ENREF_1)]: 2112.

Volders, K., et al. (2011). "The autism candidate gene Neurobeachin encodes a scaffolding protein implicated in membrane trafficking and signaling." Curr Mol Med 11[[2](#_ENREF_2)]: 204-217.

von Muhlendahl, K. E. (2005). "Commentary regarding the article by Mutter et al. "Amalgam studies: disregarding basic principles of mercury toxicity" [int. J. Hyg. Environ. Health 207 (2004) 391-397]." Int J Hyg Environ Health 208(5): 435; author reply 437-438.

Vyas, U. and N. Ranganathan (2012). "Probiotics, prebiotics, and synbiotics: Gut and beyond." Gastroenterology Research and Practice.

Wadman, M. (2008). "Autism study panned by critics." Nature 454(7202): 259.

Waite, A., et al. (2009). "The neurobiology of the dystrophin-associated glycoprotein complex." Annals of Medicine 41(5): 344-359.

Wakefield, A. J. (2003). "Measles, mumps, and rubella vaccination and autism." N Engl J Med 348(10): 951-954; author reply 951-954.

Wakefield, J. (2002). "New centers to focus on autism and other developmental disorders." Environ Health Perspect 110[[1](#_ENREF_1)]: A20-21.

Walker, L. R., et al. (2011). "A case of isolated elevated copper levels during pregnancy." J Pregnancy 2011: 385767.

Walker, S. J., et al. (2006). "Cultured lymphocytes from autistic children and non-autistic siblings up-regulate heat shock protein RNA in response to thimerosal challenge." Neurotoxicology 27(5): 685-692.

Walsh, W. J. and A. I. Usman (2007). Nutrient supplements and methods for treating autism and for preventing the onset of autism, The Health Research Institute.

Walsh, W. J. and A. I. Usman (2009). Nutrient supplements and methods for treating autism and for preventing the onset of autism, The Health Research Institute.

Walton, J. C., et al. (2013). "Neuronal nitric oxide synthase and NADPH oxidase interact to affect cognitive, affective, and social behaviors in mice." Behavioural Brain Research 256: 320-327.

Waltz, M. (2012). "Images and narratives of autism within charity discourses." Disability & Society 27(2): 219-233.

Waly, M. and R. C. Deth (2003). "IGF - I and dopamine regulate methionine synthase: a sensitive target for neurodevelopmental toxins." Society for Neuroscience Abstract Viewer and Itinerary Planner 2003: Abstract No. 318.314.

Waly, M., et al. (2004). "Activation of methionine synthase by insulin-like growth factor-1 and dopamine: a target for neurodevelopmental toxins and thimerosal." Molecular Psychiatry 9[[1](#_ENREF_1)]: 358-370.

Waly, M. I.-A. and R. Deth (2008). "Neurodevelopmental Toxins Deplete Glutathione and Inhibit Folate and Vitamin B12-Dependent Methionine Synthase Activity: A Link between Oxidative Stress and Autism." FASEB Journal 22.

Wang, L., et al. (2011). "A review of candidate urinary biomarkers for autism spectrum disorder." Biomarkers 16(7): 537-552.

Warburton, D., et al. (2013). "Environmental pollution in Mongolia: Effects across the lifespan." Environmental Research 124: 65-66.

Wassili, J. H. and C. Baradaeus (2012). "Ghetto poverty and pollution in Egypt: Adeadly threat for western countries caused by new and infectious mutants. A cultural, social and microbiological synopsis." Inflammation and Allergy - Drug Targets 11(5): 406-419.

Weber, W. and S. Newmark (2007). "Complementary and Alternative Medical Therapies for Attention-Deficit/Hyperactivity Disorder and Autism." Pediatric Clinics of North America 54(6): 983-1006.

Weiss, B. (2011). "Endocrine disruptors as a threat to neurological function." Journal of the Neurological Sciences 305(1-2): 11-21.

Weiss, B. and P. J. Landrigan (2000). "The developing brain and the environment: An introduction." Environmental Health Perspectives 108(SUPPL. 3): 373-374.

Weisser, K., et al. (2004). "[Thiomersal and immunisations]." Bundesgesundheitsblatt Gesundheitsforschung Gesundheitsschutz 47(12): 1165-1174.

Weissmann, G. (2011). "Is Drug Development Too Slow? NIH to the Rescue!" FASEB Journal 25[[1](#_ENREF_1)]: 1119-1122.

Welch, M. G., et al. (2003). "Secretin activates visceral brain regions in the rat including areas abnormal in autism." Cellular and Molecular Neurobiology 23(4-5): 817-837.

Welch, M. G. and D. A. Ruggiero (2005). Predicted role of secretin and oxytocin in the treatment of behavioral and developmental disorders: Implications for autism. Gaba in Autism and Related Disorders. D. M. Dhossche. San Diego, Elsevier Academic Press Inc. 71: 273-315.

Wertz, P. W. (2009). "Essential fatty acids and dietary stress." Toxicology and Industrial Health 25(4-5): 279-283.

Wetterberg, L. (1999). "[Complex hereditary diseases with psychiatric symptoms]." Tidsskr Nor Laegeforen 119(6): 839-845.

Wetterberg, L. (2000). "[Mental disease a heritage. New genetic knowledge can reveal "public diseases" such as autism, dyslexia, alcoholism, anorexia, schizophrenia]." Lakartidningen 97(6): 558-562, 565-557.

Whelan, E. M. (2008). "The top ten unfounded health scares of the year." MedGenMed Medscape General Medicine 10(2).

White, T., et al. (2006). "The schizophrenia prodrome." American Journal of Psychiatry 163[[2](#_ENREF_2)]: 376-380.

Wignyosumarto, S., et al. (1992). "EPIDEMIOLOGICAL AND CLINICAL STUDY OF AUTISTIC CHILDREN IN YOGYAKARTA INDONESIA." Kobe Journal of Medical Sciences 38[[1](#_ENREF_1)]: 1-19.

Williams, A. L. and J. M. DeSesso (2014). "Gestational/ Perinatal chlorpyrifos exposure is not associated with autistic-like behaviors in rodents." Critical Reviews in Toxicology 44(6): 523-534.

Williams, E. L. and M. F. Casanova (2010). "Potential teratogenic effects of ultrasound on corticogenesis: implications for autism." Med Hypotheses 75[[1](#_ENREF_1)]: 53-58.

Williams, P. G., et al. (2008). "A controlled study of mercury levels in hair samples of children with autism as compared to their typically developing siblings." Research in Autism Spectrum Disorders 2[[1](#_ENREF_1)]: 170-175.

Williams, T. A., et al. (2007). "Risk of autistic disorder in affected offspring of mothers with a glutathione S-transferase Pl haplotype." Archives of Pediatrics & Adolescent Medicine 161[[1](#_ENREF_1)]: 356-361.

Wilson, D. A., et al. (2014). "Cortical odor processing in health and disease." Prog Brain Res 208: 275-305.

Wine, O., et al. (2014). "Using pollutant release and transfer register data in human health research: a scoping review." Environmental Reviews 22[[1](#_ENREF_1)]: 51-65.

Winneke, G. (2011). "Developmental aspects of environmental neurotoxicology: lessons from lead and polychlorinated biphenyls." J Neurol Sci 308(1-2): 9-15.

Witter, F. R., et al. (2010). "Reply." American Journal of Obstetrics and Gynecology 203[[1](#_ENREF_1)]: e14-e15.

Wolowiec, P., et al. (2013). "Hair analysis in health assessment." Clinica Chimica Acta 419: 139-171.

Wolterink, G., et al. (2001). "Early amygdala damage in the rat as a model for neurodevelopmental psychopathological disorders." European Neuropsychopharmacology 11[[1](#_ENREF_1)]: 51-59.

Woodruff, T. J., et al. (2010). "Research agenda for environmental reproductive health in the 21st century." Journal of Epidemiology & Community Health 64[[1](#_ENREF_1)]: 307-310.

Woods, A. G., et al. (2013). "Mass spectrometry as a tool for studying autism spectrum disorder." Journal of Molecular Psychiatry 1[[1](#_ENREF_1)].

Woods, J. S., et al. (2010). "Urinary porphyrin excretion in neurotypical and autistic children." Environ Health Perspect 118(10): 1450-1457.

Woodward, G. (2001). "Autism and Parkinson's disease." Med Hypotheses 56(2): 246-249.

Woolf, A. D. (2014). "Aluminum toxicity in infants & children: What's known and what isn't." Clinical Toxicology 52: 346-347.

Woolf, A. D., et al. (2007). "Update on the Clinical Management of Childhood Lead Poisoning." Pediatric Clinics of North America 54(2): 271-294.

Worth, J. (2002). "Organophosphate infantile poisoning [2]." Journal of Nutritional and Environmental Medicine 12(2): 118.

Worth, J. (2002). "Paraoxonase polymorphisms and organophosphates." Lancet (North American Edition) 360(9335): 802-803.

Wright, B., et al. (2012). "A comparison of urinary mercury between children with autism spectrum disorders and control children." PLoS One 7(2): e29547.

Wright, T. and M. Wolery (2011). "The effects of instructional interventions related to street crossing and individuals with disabilities." Research in Developmental Disabilities 32(5): 1455-1463.

Xia, G. H., et al. (2011). "Single Nucleotide Polymorphisms Analysis of Noise-Induced Hearing Loss Using Three-Dimensional Polyacrylamide Gel-Based Microarray Method." Journal of Biomedical Nanotechnology 7(6): 807-812.

Xu, J., et al. (2013). "Neurotoxic effects of nonylphenol: a review." Wiener Klinische Wochenschrift 125(3-4): 61-70.

Xue, H. and I. Gertner (2014). "Automatic Recognition of Emotions from Facial Expressions." Automatic Target Recognition Xxiv 9090: 12.

Yahraes, H. and D. J. Cohen (1979). "New light on autism and other puzzling disorders of childhood." FAMILIES TODAY, NIMH SCI. MONOGRAPH, WASHINGTON DC: 743-765.

Yamada, M. and K. Konagaya (1987). "ANESTHETIC MANAGEMENT IN DENTISTRY FOR SEVERELY HANDICAPPED PATIENTS IN OKINAWA-KEN JAPAN." Aichi-Gakuin Journal of Dental Science 25(2): 250-259.

Yasuda, H. and T. Tsutsui (2013). "Assessment of infantile mineral imbalances in autism spectrum disorders (ASDs)." Int J Environ Res Public Health 10[[1](#_ENREF_1)]: 6027-6043.

Yasuda, H. and T. Tsutsui (2013). "Zinc- and Magnesium-Deficiency and Toxic Metal Burdens in the Infants with Autistic Disorders." Journal of Pharmacological Sciences 121: 123P-123P.

Yasuda, H., et al. (2013). "Estimation of autistic children by metallomics analysis." Sci Rep 3: 1199.

Yasuda, H., et al. (2011). "Infantile zinc deficiency: association with autism spectrum disorders." Sci Rep 1: 129.

Yates, A., et al. (1987). "EFFECT OF NEGATIVE AIR IONIZATION ON HYPERACTIVE AND AUTISTIC CHILDREN." American Journal of Physical Medicine 66(5): 264-268.

Yau, V. M., et al. (2014). "Prenatal and neonatal peripheral blood mercury levels and autism spectrum disorders." Environ Res 133: 294-303.

Yeargin-Allsopp, M. and C. Boyle (2002). "Overview: the epidemiology of neurodevelopmental disorders." Ment Retard Dev Disabil Res Rev 8[[2](#_ENREF_2)]: 113-116.

Yochum, C. L., et al. (2010). "Animal model of autism using GSTM1 knockout mice and early post-natal sodium valproate treatment." Behavioural Brain Research 210(2): 202-210.

Yoo, H. J. (2013). "Recent Increase in Autism and ADHD: True or Inflated?" Journal of Korean Medical Science 28(7): 974-975.

Yoo, J. H., et al. (2007). "Relevance of donepezil in enhancing learning and memory in special populations: A review of the literature." Journal of Autism and Developmental Disorders 37(10): 1883-1901.

Yorbik, O., et al. (2004). "Zinc status in autistic children." Journal of Trace Elements in Experimental Medicine 17(2): 101-107.

Yorbik, O., et al. (2010). "Chromium, cadmium, and lead levels in urine of children with autism and typically developing controls." Biol Trace Elem Res 135(1-3): 10-15.

Yoshimasu Hygiene, K. (2013). "Childhood autism, ADHD, and mercury exposures: A meta-analysis." European Psychiatry 28.

Yoshimasu, K., et al. (2014). "A meta-analysis of the evidence on the impact of prenatal and early infancy exposures to mercury on autism and attention deficit/hyperactivity disorder in the childhood." Neurotoxicology 44: 121-131.

Youn, S. I., et al. (2010). "Porphyrinuria in Korean children with autism: correlation with oxidative stress." J Toxicol Environ Health A 73(10): 701-710.

Young, H. A., et al. (2008). "Thimerosal exposure in infants and neurodevelopmental disorders: An assessment of computerized medical records in the Vaccine Safety Datalink." Journal of the Neurological Sciences 271(1-2): 110-118.

Young-Chung, N., et al. (2010). "Two cases of critical airway management in extremely obese children." Critical Care Medicine 38: A274.

Zahir, F., et al. (2005). "Low dose mercury toxicity and human health." Environ Toxicol Pharmacol 20(2): 351-360.

Zeidan-Chulia, F., et al. (2014). "The glial perspective of autism spectrum disorders." Neuroscience and Biobehavioral Reviews 38: 160-172.

Zeliger, H. I. (2011). Autism: Effect of Maternal Exposure to Neurotoxic Chemicals, William Andrew Inc, 13 Eaton Ave, Norwich, Ny 13815 USA.

Zhang, H., et al. (2005). "High levels of nitric oxide in both serum and cerebrospinal fluid in individuals with autism." Neurology 64(6): A342-A342.

Zhang, J. and K. R. Smith (2003). "Indoor air pollution: a global health concern." Br Med Bull 68: 209-225.

Zhang, J. L., et al. (2013). "Neonatal citalopram exposure decreases serotonergic fiber density in the olfactory bulb of male but not female adult rats." Frontiers in Cellular Neuroscience 7: 8.

Zhang, L. and M. H. Wong (2007). "Environmental mercury contamination in China: sources and impacts." Environ Int 33[[1](#_ENREF_1)]: 108-121.

Zhu, C. B., et al. (2011). "Colocalization and regulated physical association of presynaptic serotonin transporters with A[[2](#_ENREF_2)] adenosine receptors." Mol Pharmacol 80[[2](#_ENREF_2)]: 458-465.

Zhu, H. P., et al. (2011). "Electroencephalogram evidence for mirror neuron activity during the observation of drawn hand motion." Neural Regeneration Research 6(18): 1398-1403.

Zilbovicius, M., et al. (1996). "Study of the neurobiological mechanisms of infantile autism by cerebral imaging techniques." Medecine Nucleaire 20(7-8): 469.

Zilbovicius, M., et al. (1989). "SPECT RCBF CHANGES AND EVOKED POTENTIAL RESPONSE DURING AUDITORY STIMULATIONS IN CHILDHOOD AUTISM." European Journal of Nuclear Medicine 15[[1](#_ENREF_1)]: 407.

Zilbovicius, M., et al. (1995). "Study of childhood autism using neurofunctional imaging." Circulation et Metabolisme du Cerveau 12[[1](#_ENREF_1)]: 27-38.

Zilbovicius, M., et al. (1993). "Abnormal rCBF response to auditory stimulation in childhood autism: A SPECT study." Journal of Nuclear Medicine 34(5 SUPPL.): 78P.

Zilbovicius, M., et al. (1995). "Delayed maturation of the frontal cortex in childhood autism." American Journal of Psychiatry 152(2): 248-252.

Zilbovicius, M., et al. (1992). "SPECT evidence of delayed frontal cortex maturation in childhood autism." Society for Neuroscience Abstracts 18(1-2): 1445.

Zilbovicius, M., et al. (1992). "REGIONAL CEREBRAL BLOOD FLOW IN CHILDHOOD AUTISM A SPECT STUDY." American Journal of Psychiatry 149(7): 924-930.

Zilbovicius, M., et al. (1991). "SPECT RCBF STUDY IN CHILDHOOD AUTISM." Journal of Nuclear Medicine 32(5 SUPPL): 1086.

Zimmerman, A. W., et al. (2005). "Cerebrospinal fluid and serum markers of inflammation in autism." Pediatric Neurology 33[[2](#_ENREF_2)]: 195-201.

Zimmerman, R. K., et al. (2005). "Vaccine criticism on the world wide web." Journal of Medical Internet Research 7(2).

Zingg, W. (2005). "[Does vaccination cause disease?]." Ther Umsch 62(10): 665-674.

Zoroglu, S. S., et al. (2003). "Pathophysiological role of nitric oxide and adrenomedullin in autism." Cell Biochem Funct 21[[1](#_ENREF_1)]: 55-60.
