# Supplementary material for: A Systematic Review and Meta-Analysis of Multiple Airborne Pollutants and Autism Spectrum Disorder
Source: PLoS One. 2016 Sep 21;11(9):e0161851. doi: 10.1371/journal.pone.0161851 (PMC5031428; doi:10.1371/journal.pone.0161851)
Supplement: S1 Table — (DOCX) [file pone.0161851.s005.docx]

**S1 Table. Database-specific search terms**

*PubMed search strategy:*

| **Search** | **PubMed** |
| --- | --- |
| #1  Exposure terms (air/inhalation exposure) | (“Air Pollution” [mh:noexp] OR "Air Pollution, Indoor" [mh]) OR  (“Air Pollutants” [mh:noexp] OR "Air Pollutants, Occupational" [mh]) OR  Inhalation [mh] OR  (“Air Pollution” [tiab] OR “Air Pollutant” [tiab] OR “Air Pollutants” [tiab]) OR  (“indoor air” [tiab] OR “indoor particles” [tiab]) OR  “Ambient Air” [tiab] OR  (“Airborne Particle” [tiab] OR “Airborne Particles” [tiab]) OR  (“Airborne Pollutant” [tiab] OR “Airborne Pollutants” [tiab]) OR  (“Traffic Pollution” [tiab] OR “Traffic Pollutant” [tiab] OR “Traffic Pollutants” [tiab]) OR  “Air Quality” [tiab] OR  “HAPS” [tiab] OR  (“Air Toxic” [tiab] OR “Air Toxics” [tiab]) OR  (Inhalation [tiab] OR Inhale [tiab] OR Inhaled [tiab]) |

| **Search** | **PubMed** |
| --- | --- |
| #2  Exposure terms (chemical composition of air pollution) | Ozone [mh] OR  “Carbon Monoxide” [mh] OR  “Nitrogen Dioxide” [mh] OR  “Sulfur Dioxide” [mh] OR  “Hydrogen Sulfide” [mh] OR  “Particulate Matter” [mh:noexp] OR “Coal Ash” [mh] OR Dust [mh:noexp] OR Smog [mh] OR Smoke [mh:noexp] OR Soot [mh] OR  “Vehicle Emissions” [mh] OR  “Motor Vehicles” [mh] OR  "Polycyclic Hydrocarbons, Aromatic"[mh:noexp] OR  "Benzo(a)pyrene" [mh] OR  Benzene [mh] OR  "Fossil Fuels" [mh] OR  “Metals, Heavy” [mh] OR  “Volatile Organic Compounds” [mh] OR  "Pesticides" [mh] OR  (Ozone [tiab] OR O3 [tiab]) OR  “Carbon Monoxide” [tiab] OR  ("Nitrogen Dioxide" [tiab] OR “NO(x)” [tiab] OR NOx [tiab] OR NO2 [tiab]) OR  ("Nitrogen Oxide" [tiab] OR "Nitrogen Oxides" [tiab]) OR  “Nitric Oxide” [tiab] OR  (“Sulfur Dioxide” [tiab] OR SO2 [tiab]) OR  (“Hydrogen Sulfide” [tiab] OR H2S [tiab]) OR  “Particulate Matter” [tiab] OR  (PM2.5 [tiab] OR “PM(2.5)” [tiab]) OR  (PM10 [tiab] OR “PM(10)” [tiab]) OR  Smog [tiab] OR  Soot [tiab] OR  Dust [tiab] OR  ((Vehicle [tiab] OR Vehicles [tiab] OR Vehicular [tiab] OR Auto [tiab] OR Automobile [tiab] OR Bus [tiab] OR Buses [tiab] OR Car [tiab] OR Cars [tiab] OR Truck [tiab] OR Trucks [tiab] OR Engine [tiab] OR Transport [tiab] OR Transportation [tiab]) AND (Emissions [tiab] OR Exhaust [tiab] OR Fume [tiab] OR Fumes [tiab])) OR  (Traffic [tiab] NOT (Safety [tiab] OR Accident* [tiab] OR Injur* [tiab] OR Collision [tiab] OR Collisions [tiab] OR Crash*[tiab])) OR  ((Proximity [tiab] OR Near [tiab]) AND (Road [tiab] OR Roadways [tiab] OR Highway [tiab] OR Highways [tiab] OR Freeway [tiab] OR Freeways [tiab] OR Motorway [tiab] OR Motorways [tiab])) OR  “Polycyclic Aromatic Hydrocarbons” [tiab] OR  (“Benzopyrene” [tiab] OR "Benzo(a)pyrene"[ tiab] OR “3, 4-Benzopyrene” [tiab] OR Benzene [tiab]) OR  “Fossil Fuels” [tiab] OR  (“Carbon Black” [tiab] OR “Black Carbon” [tiab]) OR  “Elemental Carbon” [tiab] OR  ("Volatile Organic Compounds" [tiab] OR Gasoline [tiab] OR Diesel [tiab] OR Petrol [tiab] OR Petroleum [tiab] OR Petrochemical [tiab] OR Petrochemicals [tiab]) OR  (Metal [tiab] OR Metals [tiab]) OR  ((Air [tiab] OR Airborne [tiab] OR Coarse [tiab] OR Ultrafine [tiab] OR Fine [tiab]) AND (Particle [tiab] OR Particles [tiab] OR Particulate [tiab] OR Particulates [tiab])) OR  (Pesticide [tiab] OR Pesticides[tiab]) OR  (Industr* [tiab] OR Factory [tiab] OR Factories [tiab] OR Manufacturing-plant* [tiab] OR Smokestack* [tiab] OR Smoke-stack* [tiab] OR “Point source” [tiab] OR Power-plant* [tiab] OR “Residential proximity” [tiab] OR “Maternal residence” [tiab]) |

| **Search** | **PubMed** |
| --- | --- |
| #3 | "Occupational Exposure" [mh] OR  ("Occupational Exposure" [tiab] OR "Occupational Exposures" [tiab]) |

| **Search** | **PubMed** |
| --- | --- |
| #4  Outcome terms | “Child Development Disorders, Pervasive” [mh] OR  (Autism [tiab] OR Autistic [tiab]) OR  “Autism Spectrum Disorder” [tiab] OR  (Asperger [tiab] OR Asperger’s [tiab]) OR  “Pervasive Developmental Disorder” [tiab] OR  “PDD-NOS” [tiab] |

| **Search** | **PubMed** |
| --- | --- |
| **#5** | **#1 OR #2 OR #3** |
| **#6** | **#4 AND #5** |

*Web of Science and Biosis Previews:*

| **Search** | **Web of Science & Biosis Previews** |
| --- | --- |
| #1  Exposure terms (air/inhalation exposure) | (“Air Pollution” OR “Air Pollutant” OR “Air Pollutants”) OR  (“Indoor Air” OR “Indoor Particles”) OR  “Ambient Air” OR  (“Airborne Particle” OR “Airborne Particles”) OR  (“Airborne Pollutant” OR “Airborne Pollutants”) OR  (“Traffic Pollution” OR “Traffic Pollutant” OR “Traffic Pollutants”) OR  “Air Quality” OR  “HAPS” OR  (“Air Toxic” OR “Air Toxics”) OR  (Inhalation OR Inhale OR Inhaled) |

| **Search** | **Web of Science & Biosis Previews** |
| --- | --- |
| #2  Exposure terms (chemical composition of air pollution) | (Ozone OR O3) OR  “Carbon Monoxide” OR  ("Nitrogen Dioxide" OR “NO(x)” OR NOx OR NO2) OR  ("Nitrogen Oxide" OR "Nitrogen Oxides") OR  “Nitric Oxide” OR  (“Sulfur Dioxide” OR SO2) OR  (“Hydrogen Sulfide” OR H2S) OR  “Particulate Matter” OR  (“PM2.5” OR “PM(2.5)”) OR  (“PM10” OR “PM(10)”) OR  Smog OR  Soot OR  Dust OR  ((Vehicle OR Vehicles OR Vehicular OR Auto OR Automobile OR Bus OR Buses OR Car OR Cars OR Truck OR Trucks OR Engine OR Transport OR Transportation) AND (Emissions OR Exhaust OR Fume OR Fumes)) OR  (“Traffic” NOT (Safety OR Accident* OR Injur* OR Collision OR Collisions OR Crash*)) OR  ((Proximity OR “Near”) AND (Road OR Roadways OR Highway OR Highways OR Freeway OR Freeways OR Motorway OR Motorways)) OR  “Polycyclic Aromatic Hydrocarbons” OR  (“Benzopyrene” OR "Benzo(a)pyrene" OR “3, 4-Benzopyrene” OR Benzene) OR  “Fossil Fuels” OR  (“Carbon Black” OR “Black Carbon”) OR  “Elemental Carbon” OR  ("Volatile Organic Compounds" OR Gasoline OR Diesel OR Petrol OR Petroleum OR Petrochemical OR Petrochemicals) OR  (Metal OR Metals) OR  ((Air OR Airborne OR Coarse OR Ultrafine OR Fine) AND (Particle OR Particles OR Particulate OR Particulates)) OR  (Pesticide OR Pesticides) OR  (Industr* OR Factory OR Factories OR “Manufacturing plant” OR “Manufacturing Plants” OR Smokestack OR “Smoke Stack” OR “Smoke Stacks” OR “Point Source” OR “Power Plant” OR “Power Plants” OR “Residential Proximity” OR “Maternal Residence”) |

| **Search** | **Web of Science & Biosis Previews** |
| --- | --- |
| #3 | ("Occupational Exposure" OR "Occupational Exposures") |

| **Search** | **Web of Science & Biosis Previews** |
| --- | --- |
| #4  Outcome | (Autism OR Autistic) OR  “Autism Spectrum Disorder” OR  (Asperger OR Asperger’s) OR  “Pervasive Developmental Disorder” OR  “PDD-NOS” |

| **Search** | **Web of Science & Biosis Previews** |
| --- | --- |
| **#5** | **#1 OR #2 OR #3** |
| **#6** | **#4 AND #5** |

*Embase:*

| **Search** | **Embase** |
| --- | --- |
| #1  Exposure terms (air/inhalation exposure) | ('Air Pollution'/de OR 'Air Pollutant'/exp OR 'Indoor Air Pollution'/de) OR  'Ambient Air'/de OR  'Airborne Particle'/de OR  'Air Quality'/de OR  'Inhalation'/de OR  'Aerosol'/de OR  (‘Air Pollution’:ti,ab OR ‘Air Pollutant’:ti,ab OR ‘Air Pollutants’:ti,ab) OR  (‘Indoor Air’:ti,ab OR ‘Indoor Particles’:ti,ab) OR  ‘Ambient Air’:ti,ab OR  (‘Airborne Particle’:ti,ab OR ‘Airborne Particles’:ti,ab) OR  (‘Airborne Pollutant’:ti,ab OR ‘Airborne Pollutants’:ti,ab) OR  (‘Traffic Pollution’:ti,ab OR ‘Traffic Pollutant’:ti,ab OR ‘Traffic Pollutants’:ti,ab) OR  ‘Air Quality’:ti,ab OR  ‘HAPS’:ti,ab OR  (‘Air Toxic’:ti,ab OR ‘Air Toxics’:ti,ab) OR  (Inhalation:ti,ab OR Inhale:ti,ab OR Inhaled:ti,ab) |

| **Search** | **Embase** |
| --- | --- |
| #2  Exposure terms (chemical composition of air pollution) | 'Ozone'/de OR  'Carbon Monoxide'/de OR  ('Nitrogen Dioxide'/de OR 'Nitrogen Oxide'/de OR 'Nitrous Oxide Emission'/de) OR  ('Sulfur Dioxide'/de OR 'Hydrogen Sulfide'/de) OR  ('Particulate Matter'/de OR ‘Dust and Dust Related Phenomena'/exp) OR  ('Traffic'/de OR ‘Highway'/de) OR  'Polycyclic Aromatic Hydrocarbon'/de OR  ‘Benzo(a)pyrene'/de OR  'Benzene'/de OR  'Fossil Fuel'/de OR  'Heavy Metal'/exp OR  'Volatile Organic Compound'/de OR  'Pesticide'/exp OR  (Ozone:ti,ab OR O3:ti,ab) OR  ‘Carbon Monoxide’:ti,ab OR  (‘Nitrogen Dioxide’:ti,ab OR ’NO(x)’:ti,ab OR ‘NOx’:ti,ab OR ‘NO2’:ti,ab) OR  (‘Nitrogen Oxide’:ti,ab OR ‘Nitrogen Oxides’:ti,ab) OR  ‘Nitric Oxide’:ti,ab OR  (‘Sulfur Dioxide’:ti,ab OR ‘SO2’:ti,ab) OR  (‘Hydrogen Sulfide’:ti,ab OR ‘H2S’:ti,ab) OR  ‘Particulate Matter’:ti,ab OR  (‘PM2.5’:ti,ab OR ‘PM(2.5)’:ti,ab) OR  (‘PM10’:ti,ab OR ‘PM(10)’:ti,ab) OR  Smog:ti,ab OR  Soot:ti,ab OR  Dust:ti,ab OR  ((Vehicle:ti,ab OR Vehicles:ti,ab OR Vehicular:ti,ab OR Auto:ti,ab OR Automobile:ti,ab OR Bus:ti,ab OR Buses:ti,ab OR Car:ti,ab OR Cars:ti,ab OR Truck:ti,ab OR Trucks:ti,ab OR Engine:ti,ab OR Transport:ti,ab OR Transportation:ti,ab) AND (Emissions:ti,ab OR Exhaust:ti,ab OR Fume:ti,ab OR Fumes:ti,ab)) OR  (Traffic:ti,ab NOT (Safety:ti,ab OR Accident*:ti,ab OR Injur*:ti,ab OR Collision:ti,ab OR Collisions:ti,ab OR Crash*:ti,ab)) OR  ((Proximity:ti,ab OR Near:ti,ab) AND (Road:ti,ab OR Roadways:ti,ab OR Highway:ti,ab OR Highways:ti,ab OR Freeway:ti,ab OR Freeways:ti,ab OR Motorway:ti,ab OR Motorways:ti,ab)) OR  ‘Polycyclic Aromatic Hydrocarbons’:ti,ab OR  (‘Benzopyrene’:ti,ab OR ‘Benzo(a)pyrene’:ti,ab OR ‘3, 4-Benzopyrene’:ti,ab OR Benzene:ti,ab) OR  ‘Fossil Fuels’:ti,ab OR  (‘Carbon Black’:ti,ab OR ‘Black Carbon’:ti,ab) OR  ‘Elemental Carbon’:ti,ab OR  (‘Volatile Organic Compounds’:ti,ab OR Gasoline:ti,ab OR Diesel:ti,ab OR Petrol:ti,ab OR Petroleum:ti,ab OR Petrochemical:ti,ab OR Petrochemicals:ti,ab) OR  (Metal:ti,ab OR Metals:ti,ab) OR  ((Air:ti,ab OR Airborne:ti,ab OR Coarse:ti,ab OR Ultrafine:ti,ab OR Fine:ti,ab) AND (Particle:ti,ab OR Particles:ti,ab OR Particulate:ti,ab OR Particulates:ti,ab)) OR  (Pesticide:ti,ab OR Pesticides:ti,ab) OR  (Industr*:ti,ab OR Factory:ti,ab OR Factories:ti,ab OR ‘Manufacturing plant’:ti,ab OR ‘Manufacturing Plants’:ti,ab OR Smokestack:ti,ab OR ‘Smoke Stack’:ti,ab OR ‘Smoke Stacks’:ti,ab OR ‘Point Source’:ti,ab OR ‘Power Plant’:ti,ab OR ‘Power Plants’:ti,ab OR ‘Residential Proximity’:ti,ab OR ‘Maternal Residence’:ti,ab) |

| **Search** | **Embase** |
| --- | --- |
| #3 | 'Occupational Exposure'/exp OR  (‘Occupational Exposure’:ti,ab OR ‘Occupational Exposures’:ti,ab) |

*Toxline:*

| **Search** | **Toxline** |
| --- | --- |
| #1  Exposure Terms (air/inhalation exposure) | (“Air Pollution” [mh:noexp] OR  "Air Pollution, Indoor" [mh]) OR  (“Air Pollutants” [mh:noexp] OR "Air Pollutants, Occupational" [mh]) OR  Inhalation [mh] OR  (“Air Pollution” OR “Air Pollutant” OR “Air Pollutants”) OR  (“indoor air” OR “indoor particles”) OR  “Ambient Air” OR  (“Airborne Particle” OR “Airborne Particles”) OR  (“Airborne Pollutant” OR “Airborne Pollutants”) OR  (“Traffic Pollution” OR “Traffic Pollutant” OR “Traffic Pollutants”) OR  “Air Quality” OR  “HAPS” OR  (“Air Toxic” OR “Air Toxics”) OR  (Inhalation OR Inhale OR Inhaled) |

| **Search** |  | **Toxline** |
| --- | --- | --- |
| #2*  Specific chemicals and types of air pollution | #2a | Ozone [mh] OR 10028-15-6[RN]  “Carbon Monoxide” [mh] OR 630-08-0[RN]  “Nitrogen Dioxide” [mh] OR 10102-44-0[RN]  “Sulfur Dioxide” [mh] OR 7446-09-5[RN]  “Hydrogen Sulfide” [mh] OR 7783-06-4[RN]  “Particulate Matter” [mh:noexp] OR “Coal Ash” [mh] OR Dust [mh:noexp] OR Smog [mh] OR Smoke [mh:noexp] OR Soot [mh] OR  “Vehicle Emissions” [mh] OR  “Motor Vehicles” [mh] OR  "Polycyclic Hydrocarbons, Aromatic"[mh:noexp] OR 130498-29-2[RN]  "Benzo(a)pyrene" [mh] OR 50-32-8[RN]  Benzene [mh] OR 71-43-2[RN]  "Fossil Fuels" [mh] OR  “Metals, Heavy” [mh] OR  “Volatile Organic Compounds” [mh] OR  "Pesticides" [mh] OR  (Ozone OR O3) OR  “Carbon Monoxide” OR  ("Nitrogen Dioxide" OR NOx OR NO2) OR  ("Nitrogen Oxide" OR "Nitrogen Oxides") OR  “Nitric Oxide” OR  (“Sulfur Dioxide” OR SO2) OR  (“Hydrogen Sulfide” OR H2S) OR  “Particulate Matter” OR  PM2.5 OR  PM10 OR  Smog OR  Soot OR  Dust |
|  | #2b | ((Vehicle OR Vehicles OR Vehicular OR Auto OR Automobile OR Bus OR Buses OR Car OR Cars OR Truck OR Trucks OR Engine OR Transport OR Transportation) AND (Emissions OR Exhaust OR Fume OR Fumes)) OR  (Traffic NOT (Safety OR Accident* OR Injur* OR Collision OR Collisions OR Crash*)) |
|  | #2c | ((Proximity OR Near) AND (Road OR Roadways OR Highway OR Highways OR Freeway OR Freeways OR Motorway OR Motorways )) OR  “Polycyclic Aromatic Hydrocarbons” OR  (“Benzopyrene” OR "Benzoapyrene" OR “3, 4-Benzopyrene” OR Benzene) OR  “Fossil Fuels” OR  (“Carbon Black” OR “Black Carbon”)OR  “Elemental Carbon” OR  ("Volatile Organic Compounds" OR Gasoline OR Diesel OR Petrol OR Petroleum OR Petrochemical OR Petrochemicals) OR  (Metal OR Metals) |
|  | #2d | ((Air OR Airborne OR Coarse OR Ultrafine OR Fine) AND (Particle OR Particles OR Particulate OR Particulates)) OR  (Pesticide OR Pesticides) |
|  | #2e | (Industry OR industrial OR industries OR Factory OR Factories OR “Manufacturing plant” OR “manufacturing plants” OR “Smoke stack” OR “Smoke stacks” OR smokestack OR smokestacks OR “Point source” OR “Power plant” OR “Power plants” OR “Residential proximity” OR “Maternal residence”) |

*Search #2 was broken into 5 different subgroup searches due to hit retrieval limitations of the Toxline database

| **Search** | **Toxline** |
| --- | --- |
| #3 | "Occupational Exposure" [[1](#_ENREF_1)] OR ("Occupational Exposure" OR "Occupational Exposures") |

| **Search** | **Toxline** |
| --- | --- |
| #4  Outcome terms | “Child Development Disorders, Pervasive” [[1](#_ENREF_1)] OR (Autism OR Autistic) OR “Autism Spectrum Disorder” OR (Asperger OR Asperger’s) OR “Pervasive Developmental Disorder” OR “PDD-NOS” |

| **Search** | **Toxline** |
| --- | --- |
| #5 | ((#1 OR #2a OR #2b OR #2c OR #2d OR #2e OR #3) AND #4 |
