# Supplementary material for: A Systematic Review and Meta-Analysis of Multiple Airborne Pollutants and Autism Spectrum Disorder
Source: PLoS One. 2016 Sep 21;11(9):e0161851. doi: 10.1371/journal.pone.0161851 (PMC5031428; doi:10.1371/journal.pone.0161851)
Supplement: S2 Table — (DOCX) [file pone.0161851.s006.docx]

**S2 Table. Toxicological websites and grey literature databases searched**

| Toxicological websites | Grey literature databases |
| --- | --- |
| - ATSDR Interaction Profiles <http://www.atsdr.cdc.gov/interactionprofiles/index.asp> - ATSDR Toxicological Profiles <http://www.atsdr.cdc.gov/toxprofiles/index.asp> - CalEPA Office of Environmental Health Hazard Assessment <http://www.oehha.ca.gov/risk.html>, <http://oehha.ca.gov/air.html> - Chem ID <http://chem.sis.nlm.nih.gov/chemidplus/> - DART <http://toxnet.nlm.nih.gov/newtoxnet/dart.htm> - EPA Acute Exposure Guideline Levels <http://www.epa.gov/oppt/aegl/chemlist.htm> - EPA IRIS internet [www.epa.gov/iris](http://www.epa.gov/iris) - EPA NEPIS and NSCEP <http://www.epa.gov/nscep/> - EPA Science Inventory <http://cfpub.epa.gov/si/> - EPA Substance Registry System <http://ofmpub.epa.gov/sor_internet/registry/substreg/searchandretrieve/substancesearch/search.do> - Health Canada First Priority List Assessments [http://www.hcsc.gc.ca/hecs sesc/exsd/psl1.htm](http://www.hcsc.gc.ca/hecs%20sesc/exsd/psl1.htm) - Health Canada Second Priority List Assessments [http://www.hcsc.gc.ca/hecs sesc/exsd/psl2.htm](http://www.hcsc.gc.ca/hecs%20sesc/exsd/psl2.htm) - Hazardous Substances Data Bank <http://toxnet.nlm.nih.gov/cgi-bin/sis/htmlgen?HSDB> - IPCS INCHEM <http://www.inchem.org/> - NIOSHTIC 2 [http://www2.cdc.gov/nioshtic 2/Nioshtic2.htm](http://www2.cdc.gov/nioshtic%202/Nioshtic2.htm) - Toxicology Data Network <http://toxnet.nlm.nih.gov/> - Toxline <http://toxnet.nlm.nih.gov/cgi-bin/sis/htmlgen?TOXLINE> - RTECS Toxcenter <http://www.cdc.gov/niosh/rtecs/default.html> - WHO assessments – CICADS, EHC <http://www.who.int/ipcs/assessment/en/> - USEPA Health and Environmental Studies Online <http://hero.epa.gov/> - FIFRA docket: <http://www.regulations.gov> | - Google: <http://www.google.com> - Google Scholar: <http://scholar.google.com/> - Database of federally-funded scientific research: Science.gov - ScienceResearch.com (Science federated search engine by Deep Web Technologies): [http://scienceresearch.com/](http://scienceresearch.com/scienceresearch/search.html) - Oaister database (an open-source repository of difficult-to-access, academically-oriented digital resources): <http://www.oclc.org/oaister> - Open Grey: <http://www.opengrey.eu/> |
