# Supplementary material for: A Systematic Review and Meta-Analysis of Multiple Airborne Pollutants and Autism Spectrum Disorder
Source: PLoS One. 2016 Sep 21;11(9):e0161851. doi: 10.1371/journal.pone.0161851 (PMC5031428; doi:10.1371/journal.pone.0161851)
Supplement: S3 Table — (DOCX) [file pone.0161851.s007.docx]

**S3 Table. Data extraction fields and description.**

| **Field** | **Tab** | **Field Type** | **Instructions** |
| --- | --- | --- | --- |
| Study Design* | Gen. Study Info. | Combo Box | Study Design: Choose the study design type from the dropdown list. |
| Chemical | Gen. Study Info. | Combo Box | Choose the chemical evaluated in the study. If the chemical does not exist in the drop down list, it needs to be associated with the assessment in the "Assessment Management Tools" of the main form. |
| Source of Funding | Gen. Study Info. | Text Box | Study funding source details: Indicate the source of funding for this study. This text may appear in appendix tables so be as concise as possible by using acronyms for common funding sources (US EPA, NSF, NIH/NIEHS) and NOT including grant numbers. Examples: US EPA, NIH/NIEHS, NIH/NIDDK. More specific information on funding, e.g.., grant number, can be added as a field note (if desired) |
| Does the author report a COI or financial disclosure? | Gen. Study Info. | Combo Box | Authors' declared conflicts of interest: Indicate whether the authors report a conflict of interest (COI) or financial disclosure. |
| COI details | Gen. Study Info. | Text Box | Conflict of Interest Details: Enter details about the COI if reported by the authors. This will not happen often. |
| Source of Funding Type | Gen. Study Info. | Combo Box | Study funding source: Select the source of funding type. |
| General Study Objectives | Gen. Study Info. | Text Box | What are the study objectives?: Enter a brief description of the study objectives. |
| Description of Population | Study Population | Text Box | Cohort: Enter the official name of the cohort or another brief description of the population studied (e.g., distinguishing feature, job occupation, type of clinical population, or work site). Only capitalize proper nouns; separate phrases with semicolons; no ending punctuation. Examples: "HEALS" "Northwestern Taiwan residents" "NHANES" "glass industry workers employed for more than 10 yrs." "Yucheng residents over age 60 exposed to contaminated rice oil and similar unexposed subjects" |
| Lifestage | Study Population | Combo Box | Select a descriptor for the population that comprises the cohort. |
| Country(ies) | Study Population | Text Box | Sites of Data Collection, Country: Select the country where the study population was observed. If multiple countries were studied, use the "add multi" button to select more than one. To save time looking through the list, you can begin typing the country name and DRAGON will provide auto-fill options. |
| Region | Study Population | Text Box | Sites of Data Collection, Region: Enter the region(s) where the study population is observed; use the most specific region information available and separate multiple entries with a semicolon (;). List US locations as: City, ST (two letter state abbreviation). If just an area, write: northwest (direction with lower case). |
| State(s) | Study Population | Combo Box | Sites of Data Collection, State: Choose the state if in United States. |
| Demographic Data Type | Study Population | Text Box | Age and Co-morbidities, variable: Enter the name of the demographic variable, such as "age" or "education status". Enter for age for all experimental groups where data are available and also for any other comorbidities listed in the study. |
| Population/Sub-group | Study Population | Text Box | Age and Co-morbidities, population: Enter the population or subgroup you want to enter data for. This might be "total population", "exposed", "unexposed", "cases", etc. |
| Demographic Description | Study Population | Text Box | Age and Co-morbidities, description: Enter a description of the demographic being entered. For example, if you chose a demographic variable that is categorical, you enter each of the categories in different rows. An example might be the demographic variable "Smoking status" where the demographic descriptions would be "Currently smoked", "Stopped Smoking within last 5 years", "Never smoked", etc. |
| Format | Study Population | Combo Box | Age and Co-morbidities, format: Select the format of the data. This will indicate whether the data presented in the paper are means, means with standard deviations, ranges, etc. |
| Centrality | Study Population | Text Box | Autopopulates based on the demographic data format. |
| Centrality Value | Study Population | Text Box | Enter the demographic value. |
| Variance | Study Population | Text Box | Autopopulates based on the demographic data format. |
| Variance Value | Study Population | Text Box | Enter the demographic value. |
| Lower Limit | Study Population | Text Box | Autopopulates based on the demographic data format. |
| Upper Limit Value | Study Population | Text Box | Enter the demographic value. |
| Evaluation of Population | Study Population | Text Box | Enter any additional population information from related studies that supplement the information from this main study. |
| Description of Reference Pop | Study Population | Text Box | Description of reference group: Provide information on how the reference population was selected or recruited. For case-control study (one with exposed and unexposed individuals), how were controls or unexposed groups selected? "controls randomly selected to frequency match the cases by age and sex." If lowest exposure group selected as the reference group, describe and note any possible issues. Example, no issues: "all subjects recruited in same manner and group with water concentrations lower than 10 ppb used as reference group." Example, potential issues: "all subjects recruited in same manner; reference group consisted of subjects exposed to<10 ppb, however, group significantly younger than other exposure groups and age not adjusted for" |
| Exclusion Criteria | Study Population | Text Box | Inclusion/exclusion criteria/recruitment strategy: Describe the criteria used to exclude subjects in the study population. Complete sentences are not necessary. List separate elements with a semicolon in between; capitalize the very first letter; do not use a period at the end. If not specified in the paper, type "Not specified". Examples: "No glucose data available" |
| Number Eligible to Participate (N) - Cases or Exposed | Study Population | Text Box | Sample size of cohort, eligible, exposed: Enter the size of the exposed population or number of cases eligible to participate in the study, if provided. For example, a cohort study might have a total population of 10,000 members although only a subset of the population was invited to participate in the specific study. |
| Number Eligible to Participate (N) - Controls or Reference | Study Population | Text Box | Sample size of cohort, eligible, reference: Enter the size of the reference or control population eligible to participate in the study, if provided. |
| Number Eligible to Participate (N) - Total Population | Study Population | Text Box | Sample size of cohort, eligible, total: Enter the size of the population eligible to participate in the study, if provided. For example, a cohort study might have a total population of 10,000 members although only a subset of the population was invited to participate in the specific study. |
| Number Invited to Participate (N) - Cases or Exposed | Study Population | Text Box | Sample size of cohort, invited, exposed:Enter the size of the exposed population before any were eliminated, missing, or lost. For case-control, enter number of cases. |
| Number Invited to Participate (N) - Controls or Reference | Study Population | Text Box | Sample size of cohort, invited, reference:Enter the size of the reference or control population before any were eliminated, missing, or lost, if provided. |
| Number Invited to Participate (N) - Total Population | Study Population | Text Box | Sample size of cohort, invited, total:Primarily relevant to cohort and cross-sectional studies: Enter the size of the population invited to participate in the study, if provided. This will be compared to the study population size in order to determine the participation rates. For example, this population may include the entire region or occupational population prior to any applied inclusion and exclusion criteria. For case-control studies, SKIP and enter target population below in Exposed Population Metrics and Reference Population Metrics. |
| Number Who Participated (N) - Cases or Exposed | Study Population | Text Box | Sample size of cohort, included, exposed:Enter the total number of subjects in the exposed group, minus any exposed subjects that were identified as a "reference" group. The sum of the exposed population size and the control population size should equal the study size. |
| Number Who Participated (N) - Controls or Reference | Study Population | Text Box | Sample size of cohort, included, reference: Enter the total number of subjects in the control or "reference" group. The sum of the exposed population size and the control/reference population size should equal the study size. |
| Number Who Participated (N) - Total Population | Study Population | Text Box | Sample size of cohort, included, total:Enter the size of the population that was eventually evaluated for exposure and health outcomes. This number should be less than or equal to the "target population" and represents the number of subjects in the study after the inclusion and exclusion criteria were applied to the target population. For example, this population may include the occupational population minus any prior smokers. Or, it may include all inhabitants of a region minus those that did not answer the door on the day of interview. May not be available for all studies or could be the sum of study populations for exposed and reference below. |
| Description of Losses in Selection and Recruitment Process | Study Population | Memo | Participation/follow-up rates: If there was a follow-up period where the health status of subjects was followed but subjects were lost from the study, explain how this loss to follow-up was addressed in the statistical analysis. Use short phrases separated by semicolon (;). End with period (.). Example: "29 subjects lost to follow-up because they moved out of the region; excluded from statistical tests since health outcome could not be determined." |
| (none) | Study Population | Combo Box | Select a qualifier if the follow-up length was equal to, less, than, or greater than a specific duration. |
| Length of Follow-up | Study Population | Text Box | Study duration: Length of the follow-up period when subjects were followed and assessed for health outcome. Enter a single number or a range as a appropriate and add units (years, months, days); only enter for cohort (prospective or retrospective) studies or nested case-control studies. Examples: "5-17 years", "2 months" |
| Enrollment/recruitment Years | Study Population | Text Box | Study dates: Enter the year or range of years when participants were followed. |
| Evaluation of Selection/recruitment Process | Study Population | Text Box | Study design details and other relevant details: enter any additional details related to study design or to the population selection as a whole. |
| Exposure Surrogate | Exposure Measurement | Combo Box | Source of exposure data: specify whether the data are from biomonitoring-blood, biomonitoring-urine, environmental monitoring (include matrix in details), emissions-based models (include specific model in details), questionnaire (include specific determinant of exposure in details), or other (specify in details). |
| Exposure Surrogate Units | Exposure Measurement | Text Box | Select the units for the exposure surrogate. If the correct units are not here, enter them as a new entry. Do not convert the units; enter them exactly as they appear in the study. |
| Exposure Measurement Level | Exposure Measurement | Combo Box | Indicate whether exposure was measured at the group or individual level in the study. Blood, serum, plasma, urine, and hair measurements are often individual, while drinking water and diet measurements are often at the group level. Exceptions do occur. |
| Format | Exposure Measurement | Combo Box | Exposure values for study group: Select the format of the data. This will indicate whether the data presented in the paper are means, means with standard deviations, ranges, etc. |
| Centrality | Exposure Measurement | Text Box | Autopopulates based on the exposure format. |
| Centrality Value | Exposure Measurement | Text Box | Enter the exposure value. |
| Variance | Exposure Measurement | Text Box | Autopopulates based on the exposure format. |
| Variance Value | Exposure Measurement | Text Box | Enter the exposure value. |
| Lower Limit | Exposure Measurement | Text Box | Autopopulates based on the exposure format. |
| Lower Limit Value | Exposure Measurement | Text Box | Enter the exposure value. |
| Upper Limit | Exposure Measurement | Text Box | Autopopulates based on the exposure format. |
| Upper Limit Value | Exposure Measurement | Text Box | Enter the exposure value. |
| Exposure Category Definition | Exposure Measurement | Text Box | Enter a text descriptor that differentiates the current exposure category definition from others in the paper or others entered in DRAGON. Click the + to expand and see the exposure levels for a category. Should correspond to exposure surrogate units (i.e., they could be added to the end of this to make a phrase). This may include multiple descriptors in order to fully distinguish the entry. Usually, similar exposure metrics that were assessed separately for men and women should be entered as two separate exposure categories. Example: "Mean blood lead concentration, men". Example: "Mean drinking water concentration, women, no kidney disease". |
| Lifestage at exposure | Exposure Measurement | Combo Box | Exposure measurement timing: Select the life stage at which exposure occurred, if specified. Use "multiple" if exposure occurred over lifetime or if population ages vary. If not specified and you cannot extrapolate, choose "cannot be determined." |
| Source | Exposure Measurement | Text Box | Source of Information: Enter the table or figure (or text) from which the exposure category and level data was extracted. Example: "Table 6, upper portion". Example: "Text, page 667, second paragraph" |
| Sex | Exposure Measurement | Combo Box | Sex: Enter the sex of subjects in each exposure category. |
| Description of Exposure | Exposure Measurement | Text Box | Exposure source details, range of concentrations, chemicals, and frequency: Enter details about the source of exposure data, list the range of concentrations of air pollution measured (list any specific components of air pollution) with units, and indicate the frequency of exposure measurements (if taken more than once) and the number of replicate measurements taken. Also, list any other chemical information here related to the air pollutants measured. Use complete sentences. |
| Exposure Sampling Year Range | Exposure Measurement | Text Box | Exposure measurement timing details: enter information about when exposure was measured, especially relative to when the outcomes were assessed. |
| Group | Exposure Measurement | Text Box | Enter the population or subgroup you want to enter data for. This might be "total population", "exposed", "unexposed", "cases", etc. |
| Evaluation of Exposure | Exposure Measurement | Text Box | Enter any additional exposure information from related studies that supplement the information from this main study. |
| Search: | Outcome Measurement | Text Box | Type a search term and select the filter button to limit the endpoint list. |
| Outcome | Outcome Measurement | Text Box | Outcomes measured, controlled vocabulary: The health outcome name is from a controlled vocabulary list. Double click from the list above to add the outcome to the list. You can also use the search bar to help find a particular word in the outcomes list. The study may call the health outcome something slightly different. Please contact a manager if you are unsure what health outcome to select. Also find the checkboxes at the end of the row. Check the first if the health outcomes have no statistical results reported in the paper. In the second, check if the statistical results are not being entered in epiDRAGON at this time. |
| Outcome Name In Study | Outcome Measurement | Text Box | Outcomes measured, exactly as in study: Enter the exact wording of the health outcome used in the study. The outcome name as entered will appear in the output tables, so some longer names may be truncated by the data extractor if appropriate. |
| Diagnostic Method | Outcome Measurement | Combo Box | Method of autism assessment, general: Select the method of diagnosis determination. You may only enter one. If multiple diagnostic types were used, include that information in the diagnostic description field. |
| Diagnostic Description | Outcome Measurement | Text Box | Method of autism assessment, detailed: Use short phrases, all lower case, separated by semicolons. This may include more detail about the method used and conclusions about how reliable the method of diagnosis was compared with other available methods. Also, if ICD codes were used to as part of the outcome assessment, indicate the codes here. Example: "cancers determined through annual health exams, home visit interviews, household registration data checks, cancer registry, and death certification" "identified by review of death certificates with ICD 9 code 188" |
| Outcome Sampling Years | Outcome Measurement | Text Box | Enter range of years when outcomes were assessed. Example: "1990-1991" "1976" |
| ICD used | Outcome Measurement | Combo Box | Enter which version of the ICD codes was used. If the author originally used an older ICD list (e.g., ICD7) and converted to a newer list (e.g., ICD9), then enter the codes from the original list and note the conversion in the Diagnostic Description field. If ICD code not mentioned, choose not reported. |
| Evaluation of Outcome Measure | Outcome Measurement | Text Box | Number of subjects analyzed and number of missing participants: Include information about how many subjects were analyzed for each outcome and how many were missing. Also, enter any outcome information from related studies that may supplement the information from this main study. |
| Statistical Finding Selections | Confounding and Analysis | Combo Box | Choose an existing statistical finding from the drop down or click Add New to begin a new Statistical Finding. |
| Description of Population Matching | Confounding and Analysis | Text Box | This field should contain only information from the study, not an evaluation of matching. For most relevant study types, a control (or reference) population must be determined. This control population should have similar demographics to the exposed population. Describe how the control population and reference population demographic statistics were assessed to ensure the reference population demographics matched the exposed population demographics. Often the relevant demographics include sex, age, and ethnicity/race. For occupational studies, the occupation of the reference group is also important. For certain chemicals or for certain health outcomes, other demographics may be important, such as education status, smoking status, or others.   Example: "A population consisting of a similar ethnic and age demographics in a neighboring region in China was selected for the control population. The paper provides information showing that age and sex were both similar in the two populations, although smoking status was not evaluated."  Example: "The study made no mention of how matching was assessed, and no demographic information is provided separately by exposure group."  Example: "The study made no mention of how matching was assessed, although the reference population was selected from amongst other United States steel-mill workers who likely had the same age profile as the exposed population." |
| Confounding Considered in Study Design | Confounding and Analysis | Text Box | Were known confounders accounted for by study design?: List all confounders that were included in the study design. This is different than the confounders that were eventually included in the statistical analysis. These confounders are ones considered when choosing either the target population (the whole population considered for the study) or the study population (those that eventually participated in the study). Use short complete sentences. Example: "Smoking status was ascertained by survey and all smokers or those living in homes with smokers were excluded from the study population." This information may overlap somewhat with the exclusion criteria. |
| Adjustment factors considered in the analysis | Confounding and Analysis | Memo | Were known confounders accounted for by the analysis, considered: Click the "Add Factors" link to enter confounders that were considered as part of the statistical analysis. This list might not include all the ones that were eventually included in the statistical model if some were found to have no effect on the results |
| Adjustment factors included in the analysis | Confounding and Analysis | Memo | Were known confounders accounted for by the analysis, included: Click the "Add Factors" link to enter confounders that were included in the statistical analysis. This list should only include those that were actually included in the ultimate statistical model. If a confounder is not on the list, type it in to add it. |
| Reason for Excluding Adjustment Factors in Final Model | Confounding and Analysis | Text Box | If some of the confounders in the "considered" list did not make it into the final "included" list, enter a description of why they were excluded. Example: "Education status was considered as a potential confounder but did not significantly affect the model." |
| Source in Study | Confounding and Analysis | Text Box | Enter the location in the study where the statistical conclusion information can be found; Table X, Page Y in the study reference or section in text (section number/title, page number) |
| Confounder | Confounding and Analysis | Text Box | Indicate which confounder was considered. Enter a new row for each confounder. |
| Dichotomous/Continuous | Confounding and Analysis | Combo Box | Indicate whether the confounder is dichotomous (categorical) or continuous. |
| Included in Final Analysis | Confounding and Analysis | Check Box | Indicate whether the current confounder was included in the analysis. |
| Description | Confounding and Analysis | Text Box | Enter any additional information regarding this confounder. For example, if the confounder is dichotomous how many levels are considered. If the confounder is continuous, indicate the evaluated range. |
| Other Comments on Confounding | Confounding and Analysis | Text Box | Enter any additional comments on confounding (e.g., if the study is evaluating an occupational exposure, indicate any co-exposures that may have occurred.) |
| Evaluation of Confounding | Confounding and Analysis | Text Box | Enter any additional confounding information from related studies that supplement the information from this main study. |
| Description of Statistical Analysis | Confounding and Analysis | Text Box | Enter information about the statistical method, including the type of regression or analysis (by name) that was used and any other information that might be pertinent to assessing the reliability of the statistical method. |
| Other Method Notes | Confounding and Analysis | Text Box | Enter any other information from the study (other than confounders, matching, outcome/exposure timing, missing data, coexposures, exposure assessment, and outcome assessment) that may introduce bias into the study and how the bias was accounted for. |
| Evaluation of Statistical and Analytical Approaches | Confounding and Analysis | Text Box | Enter any additional statistical analysis and results information from related studies that supplement the information from this main study. |
| Level | Results | Text Box |  |
| Stat. Est. | Results | Text Box | Enter the numerical value for the statistical metric for the particular exposure level/group. For the reference group, the value will be either 1 or 0. |
| Lower CI | Results | Text Box | Enter the lower end of the confidence interval. |
| Upper CI | Results | Text Box | Enter the upper end of the confidence interval. |
| CI Units | Results | Combo Box | Enter whether the confidence interval is the 95%, 90%, etc. Leave blank for reference group. Enter "NA" if not applicable. |
| # w/ Outcome | Results | Text Box | Number of people in exposure group with outcome; leave blank if not available. If the value is calculated or inferred from the study, put the value in square brackets. |
| # w/o Outcome | Results | Text Box | Number of people in exposure group without outcome; leave blank if not available. If the value is calculated or inferred from the study, put the value in square brackets. |
| Total in Group | Results | Text Box | Enter total number of people in exposure group; leave blank if not available. If the value is calculated or inferred from the study, put the value in square brackets. |
| Prevalence/Incidence | Results | Text Box | Enter the prevalence or incidence as a percentage; leave blank if not available. If the value is calculated or inferred from the study, put the value in square brackets. After the value, enter (P) if the value is a prevalence and (I) if the value is an incidence. |
| Variance | Results | Text Box | Enter the standard error or standard deviation for the statistical metric value, if given. |
| Variance Type | Results | Combo Box | Choose standard deviation or standard error to correspond to value entered in variance column. |
| P Value Qualifier | Results | Text Box | Select the appropriate qualifier that describes the p-value in the next field ("<", ">", etc.) |
| P value | Results | Text Box | If a test was done to determine whether the exposure group is statistically different from the control group (as opposed to an overall trend test across all groups), enter the p-value from the analysis here. |
| Notes | Results | Memo | Miscellaneous comments by reviewer regarding data analysis: Use phrases separated by semicolons (;) to make note of any observations pertaining to a single exposure group. |
| Results | Did author test for trend? | Did author test for trend? | Indicate whether a test for trend was performed by the study authors. |
| Results | Trend Description | Trend Description | Select whether the authors found a positive, negative, null, or unknown trend. A trend is positive if the statistical test indicates that exposure led to an increase in risk of a health outcome. |
| Results | Test Value Type | Test Value Type | Select the type of metric provided for the trend test. If the selection is not on the list, add it. |
| Results | Test Value Modifier | Test Value Modifier | Select the inequality symbol associated with the test value. |
| Results | Test Value | Test Value | Enter a p-value (or other statistic, if reported) for the author’s trend test. |
| Results | Is trend monotonic? | Is trend monotonic? | Select if the trend is monotonic (consistently increasing or consistently decreasing) or non-monotonic (e.g., goes up with increasing exposure for low exposures and goes down with increasing exposure for high exposures). |
| Results | Author Conclusion on Trend |  | Enter a supporting description in the text box describing the authors' main conclusions about the health outcome for that exposure group. Use one or a few complete sentences. Example: "The authors conclude there is no evidence of a statistical relationship |
| Results | Data Extractor Observations on Trend |  | If the authors did not perform a trend test or provide conclusions on the trend, select the option that best describes the statistical results. |
| Results | Data Extractors Comments on Trend |  | Note any of your own conclusions that are not necessarily reported by the author. |
