# Supplementary material for: A Systematic Review and Meta-Analysis of Multiple Airborne Pollutants and Autism Spectrum Disorder
Source: PLoS One. 2016 Sep 21;11(9):e0161851. doi: 10.1371/journal.pone.0161851 (PMC5031428; doi:10.1371/journal.pone.0161851)
Supplement: S4 Table — (DOCX) [file pone.0161851.s008.docx]

**S4 Table. Factors for evaluating the overall quality of a body of evidence.**

| Downgrading Factors | Summary of criteria for downgrading |
| --- | --- |
| Risk of bias | Study limitations – a substantial risk of bias across body of evidence |
| Indirectness | Evidence was not directly comparable to the question of interest (i.e., population, exposure, comparator, outcome) |
| Inconsistency | Widely different estimates of effect in similar populations (heterogeneity or variability in results) |
| Imprecision | Studies had few participants and few events (wide confidence intervals as judged by reviewers) |
| Publication Bias | Studies missing from body of evidence, resulting in an over or underestimate of true effects from exposure |
|  | |
| Upgrading Factors | Summary of criteria for upgrading |
| Large magnitude of effect | Upgraded if modeling suggested confounding alone unlikely to explain associations with large effect estimate as judged by reviewers |
| Dose response | Upgraded if consistent relationship between dose and response in one or multiple studies, and/or dose response across studies |
| Confounding minimizes effect | Upgraded if consideration of all plausible residual confounders or biases would underestimate the effect or suggest a spurious effect when results show no effect |
