# Supplementary material for: A Systematic Review and Meta-Analysis of Multiple Airborne Pollutants and Autism Spectrum Disorder
Source: PLoS One. 2016 Sep 21;11(9):e0161851. doi: 10.1371/journal.pone.0161851 (PMC5031428; doi:10.1371/journal.pone.0161851)
Supplement: S5 Table — (DOCX) [file pone.0161851.s009.docx]

**S5 Table. Individual study characteristics**

**Table A. Characteristics of Windham et al. 2006 [1]**

| Study Element | Description |
| --- | --- |
| Design | Case-control study |
| Participants | Source population was children born in 1994 in 6 counties in the San Francisco Bay area. Study group included 284 children with ASD and 657 controls. |
| Exposure | Census tract of birth residence linked to concentration estimates from the 1996 US EPA NATA database for 29 HAPs. |
| Comparator | Randomly selected from CA 1994 linked birth-infant death certificate files, matched to cases in 2:1 ratio by sex and month of birth. |
| Outcomes | ASD cases identified from records-based surveillance of children conducted by California CADDRE. |

| Bias domain | Authors’ judgment | | Support for judgment |
| --- | --- | --- | --- |
| Source population representation | Probably low | | Cases from DDS and Kaiser. Controls from birth certificate ‐ random sample with some matching. Cases linked to birth certificates and verified to be from same cohort. |
| Blinding | | Probably low | Blinding is not mentioned, but methods for case status and exposure assignment are unlikely to be susceptible to knowledge of group assignment. |
| Exposure assessment | | Probably low^*^  Probably high^+^  High^#^ | Judgment supported by 1996 US EPA National Air Toxics Assessment Table A: Composite judgments to determine overall certainty. |
| Outcome assessment | | Probably low | Children with ASD were identified through electronic files of the California DDS or Kaiser. Prior research demonstrates good diagnostic validity of DDS-identified autism cases. |
| Confounding | | Probably low | Adjusted for maternal education, age, race, and match for calendar month of birth, which accounts for season of birth. No adjust for urban/rural (unlikely to be significant as study was San Francisco Bay Area, which is mostly urban). All one year ‐ 1994. |
| Incomplete outcome data | | Probably low | No missing outcome data noted, but methods could result in missing some individuals. |
| Selective outcome reporting | | Low | All pre-specified outcomes reported in the pre-specified way. |
| Conflict of interest | | Low | Authors declare no competing financial interests. |
| Other sources of bias | | Low | No other potential sources of bias identified. |

^*^methylene chloride, trichloroethylene, aromatic solvents

^+^ perchloroethylene, vinyl chloride, endocrine disruptors, developmental toxicants, chlorinated solvents

^#^ arsenic, cadmium, chromium, lead, manganese, mercury, nickel

**Table B. Characteristics of Roberts et al. 2007 [2]**

| Study Element | Description |
| --- | --- |
| Design | Case-control study |
| Participants | Study population was children born in 1996-1998 in 19 counties in California. Study group included 465 children with ASD and 6,975 controls. |
| Exposure | Census tract of birth residence linked to California DPR database to determine proximity to application of 54 pesticides between 1995-1998. |
| Comparators | Selected from study population among full-term, normal weight live births using incidence density sampling design with date of last menstrual period as time variable in 15:1 ratio. |
| Outcomes | ASD cases identified from records-based surveillance of children conducted by California DDS. |

| Bias domain | Authors’ judgment | | Support for judgment |
| --- | --- | --- | --- |
| Source population representation | Probably low | | Autism cases were identified through the California DDS, which captures a large portion of autism cases statewide. Due to services eligibility issues, however, reliance on DDS is likely to miss mild autism cases and those families who do not need or wish to access state-supported services. Controls were identified based on birth certificate data. |
| Blinding | | Probably low | Blinding is not mentioned, but methods for case status and exposure assignment are unlikely to be susceptible to knowledge of group assignment. |
| Exposure assessment | | Probably low* | While the modeling did not account for time-activity patterns, and wind and atmospheric variables, the use of California PUR data and land use regression has been used successfully in other studies, with good reliability and validation. |
| Outcome assessment | | Probably low | Children with ASD were identified from this study population through electronic files of the California Dept of Developmental Services (DDS). Children reported by DDS at any age as receiving services for autism or with an ASD diagnostic code (e.g., DSM‐ IV) were included in the case group. Prior research demonstrates good diagnostic validity of DDS-identified autism cases. |
| Confounding | | Probably low | Analyses were adjusted for maternal age, race/ethnicity and regional center of diagnosis; incidence density sampling for selection of controls minimized confounding by temporal changes in exposure; information about change in residence during pregnancy, which occurs frequently, is likely to co-vary with other potential autism risk factors; pattern of change in residence after birth assumed to be equal between cases and controls, with sensitivity analysis providing some support for this assumption. Although there was no adjustment for sex or urbanicity, overall, confounding was adequately addressed. |
| Incomplete outcome data | | Probably low | No missing outcome data noted, but methods could result in missing some individuals. |
| Selective outcome reporting | | Low | All pre‐specified outcomes were reported in the pre‐specified way. |
| Conflict of interest | | Low | Authors report no competing interests and are employed by the state of California or academic institution. |
| Other sources of bias | | Low | No other potential sources of bias identified. |

*Cholinesterase inhibitors as a group, copper containing compounds as a group, fumigants as a group, avermectins as a group, halogenated organics as a group, N-methyl carbamates as a group, organichlorines as a group, organophosphates as a group, pyrethroids as a group, thiocarbamates as a group, 1,3-dichloropropene, bromacil acid, bifenthrin, chloropicrin, chlorpyrifos, copper sulfates, cypermethrin, dazomet, diuron, fenarimol, glyphosate, metam-sodium, methyl bromide, molinate, myclobutanil, norflurazon, oxadiazon, paraquat, trifluralin.

**Table C. Characteristics of Windham et al. 2007 [3]**

| Study Element | Description |
| --- | --- |
| Design | Case-control study |
| Participants | Study population was children born between 1996-1998 in 4 counties in southern California. Study group included 3,400 children with ASD and controls selected in a 1:10 ratio. |
| Exposure | Census tract of birth residence linked to concentration estimates from the 1996 US EPA NATA database for 29 HAPs. |
| Comparator | Controls selected in a 1:10 ratio. |
| Outcomes | ASD cases identified from records-based surveillance of children conducted by California DDS. |

| Bias domain | Authors’ judgment | | Support for judgment |
| --- | --- | --- | --- |
| Source population representation | Probably high | | Very limited information provided describing the source population (conference abstract). |
| Blinding | | Probably low | Blinding is not mentioned, but methods for case status and exposure assignment are unlikely to be susceptible to knowledge of group assignment. |
| Exposure assessment | | High^*^ | Judgment supported by 1996 US EPA National Air Toxics Assessment Table A: Composite judgments to determine overall certainty. |
| Outcome assessment | | Probably low | Children with ASD were identified from this study population through electronic files of the California Dept of Developmental Services (DDS). Children reported by DDS at any age as receiving services for autism or with an ASD diagnostic code (e.g., DSM‐ IV) were included in the case group. Prior research demonstrates good diagnostic validity of DDS-identified autism cases. |
| Confounding | | Probably high | Did not appear to account for urban residence, or time, but difficult to judge because so little information provided (conference abstract). |
| Incomplete outcome data | | Probably high | Outcome numbers and details are not thoroughly described (conference abstract). |
| Selective outcome reporting | | Probably low | Outcomes outlined in the methods of the abstract are reported on, but because limited information, it is difficult to judge (conference abstract). |
| Conflict of interest | | Probably low | Information not provided, but based on another study by the same authors that did not appear to have financial conflicts of interest. |
| Other sources of bias | | Probably low | Although there are no particular concerns, it is difficult to evaluate (conference abstract). |

^*^ HAPS

**Table D. Characteristics of Lewandowski et al. 2009 [4]**

| Study Element | Description |
| --- | --- |
| Design | Ecological study |
| Participants | Study population was students in Texas school districts for academic years 2000-2001 through 2005-2006. Study group included 7,022 children with autism and 4,050,690 controls for 2001; numbers not reported for other years. |
| Exposure | US EPA TRI data obtained for mercury for each county in Texas for 2000-2005; TRI air data for 10 additional pollutants obtained for 2001. |
| Comparator | All other children in the Texas Education Agency not classified as Autism case. |
| Outcomes | Prevalence of autism and other special education categories obtained from the Texas Education Agency Academic Excellence Indicator System. |

| Bias domain | Authors’ judgment | | Support for judgment |
| --- | --- | --- | --- |
| Source population representation | Probably low | | Used population data counts (census) and school special education for autism, so likely to be representative of the population, although would not include those outside of public schools. |
| Blinding | | Probably low | Not possible for knowledge of outcome to influence exposure given the linkage of existing data. |
| Exposure assessment | | Probably high^*^ | US EPA toxic release inventory data used, which may not result in estimating true exposure. |
| Outcome assessment | | Probably high | Diagnosis by "community professional" (authors do not provide adequate details regarding outcome assessment). |
| Confounding | | Probably high | No adjustment for maternal age and this could be important. High possibility of residual confounding with only ecologic data. |
| Incomplete outcome data | | Probably high | Authors filled in missing data two different ways in sensitivity analyses, and these results varied greatly. Unclear if capture outcomes for everyone, as only accounting for students in public school. |
| Selective outcome reporting | | Low | Endpoints identified in method section (autism and other special education endpoints) were reported on. |
| Conflict of interest | | High | Author employed by foundation funded by electric utility [5] and study examined coal‐ burning power plants. |
| Other sources of bias | | High | Ecologic study introduces bias that cannot be fully captured by other domains. |

^*^ TRI data—coal fired power plant, mercury, antimony, lead, manganese, nickel, zinc, benzene, ethylbenzene, naphthalene, trichloroethylene, sulfuric acid

**Table E. Characteristics of Kalkbrenner et al. 2010 [6]**

| Study Element | Description |
| --- | --- |
| Design | Case-control study |
| Participants | Study population was children aged 8 years in North Carolina (born in 1994 and 1996) and West Virginia (born in 1992 and 1994). Study group included 383 children with ASD and 2,829 children with speech and language impairment as controls. |
| Exposure | Census tract of birth residence linked to concentration estimates from the 1996 US EPA NATA database for 35 HAPs. |
| Comparator | All children in the surveillance system in North Carolina and West Virginia with school designation of speech and language impairment without documentation of other serious developmental problems (i.e., autism spectrum disorder, intellectual disabilities, etc.). |
| Outcomes | ASD cases and controls with speech and language impairment identified from records-based surveillance of children conducted by ADDM in North Carolina and West Virginia. |

| Bias domain | Authors’ judgment | | Support for judgment |
| --- | --- | --- | --- |
| Source population representation | Low | | No comparison to source population, but ADDM methodology should be representative as these methods are robust and well‐described. Limiting participation to children residing in the state at birth limits potential selection bias. |
| Blinding | | Probably low | There was no blinding but methods of case ascertainment and exposure imputation are unlikely to be influenced by lack of blinding and no reason to think outcome status was known when exposure assigned. |
| Exposure assessment | | Probably low^*^  Probably high^+^  High^#^ | Judgment supported by 1996 US EPA National Air Toxics Assessment Table A: Composite judgments to determine overall certainty. |
| Outcome assessment | | Probably low | Case definition used ADDM methods, relying on developmental records documenting characteristics and behaviors meeting a standardized definition of autism spectrum disorder based on DSM‐IV‐TR. Although no validation by direct clinical assessment was performed in this study, prior validation of ADDM records‐based diagnoses indicate good agreement with ASD diagnostic instruments. |
| Confounding | | Low | Many important confounders were recognized and accounted for in statistical approaches, including sex, urbanicity, state (NC vs. WV), ethnicity, maternal education, imputed household income from census data, maternal smoking (but not year or season). Sample size was too small to adequately assess difference as a function of birth years (e.g., children born before NATA model used). |
| Incomplete outcome data | | Probably low | This was not a longitudinal study, so there is no attrition. Case finding methods are likely to miss some individuals, but this risk of bias is incorporated in assessment of study group representation of source population. No mention of missing data. |
| Selective outcome reporting | | Low | Primary aims were clearly described and results of analysis fully reported in cases where measurable levels of HAPs were found. All endpoints were covered. |
| Conflict of interest | | Low | No financial conflict of interest was reported (CDC and NIH grants). All authors have a primary academic affiliation. |
| Other sources of bias | | Probably low | Controls had a diagnosis that may be related to exposure (speech and language impairment) so could lead to under‐estimates of effects. Generally low levels of HAPs, so harder to find effects. Accounted for air as primary source of chemical (may decrease estimates). |

^*^ acrylonitrile, benzene, carbon tetrachloride, ethylene oxide, methylene chloride, styrene, toluene, trichloroethylene, xylenes

^+^ 1,3-butadiene, chloroform, coke oven emissions, diesel particulate matter, ethyl benzene, ethylene dibromide, ethylene dichloride, formaldehyde, perchloroethylene, propylene dichloride, 1,1,2,2 tetrachloroethane, vinyl chlorine

^#^ acetaldehyde, acrolein, arsenic compounds, beryllium compounds, cadmium compounds, chromium compounds, 1-3 dichlorpropene, hexachlorobezene, hexane, hydrazine, lead compounds, manganese compounds, mercury compounds, methyl tert butyl ether (MTBE), nickel compounds, polychlorinated biphenyls, polycyclic aromatic hydrocarbons group (PAH7), propionaldehyde, quinoline

**Table F. Characteristics of Trousdale et al. 2010 [7]**

| Study Element | Description |
| --- | --- |
| Design | Cross-sectional study |
| Participants | Study population was all children aged 8 years by state (and by county for sub-analysis) during school years 2004-2005 and 2007-2008. Study group was not reported. |
| Exposure | Concentrations for 33 HAPs plus diesel particulate matter and 88 HAPs plus diesel particulate matter were obtained from the 1996 and 1999 US EPA NATA database, respectively. |
| Comparator | All children in education records not classified as autism case. |
| Outcomes | ASD prevalence calculated using data from the U.S. Department of Education, Office of Special Education Programs and control numbers using data from the National Center for Education Statistics enrollment data (Maryland sub-analysis from Maryland State Department of Education). |

| Bias domain | Authors’ judgment | | Support for judgment |
| --- | --- | --- | --- |
| Source population representation | Probably high | | DOE data from federal sources and state sources is prone to bias ‐ disability code "autism" used inconsistently across states and also no standard practice on how it is evaluated. |
| Blinding | | Probably low | No mention of blinding, but used special education data which was collected for a different purpose. |
| Exposure assessment | | High^*^ | Exposure aggregated at the state level--poor resolution. Regression models isolated ~4 exposures that accounted for most of the variance. |
| Outcome assessment | | Probably low | Based on special education classification; while they used educational records, which should have relatively robust methods for assessing outcome, this data mixed in terms of how it was obtained. |
| Confounding | | Probably high | Authors do not address some important cofounders, such as maternal age and calendar time. |
| Incomplete outcome data | | Probably low | No information available, but prevalence data available so likely have all outcome data. |
| Selective outcome reporting | | Probably high | Not enough information provided to determine whether all outcomes were reported |
| Conflict of interest | | Probably low | Not reported, but a dissertation thesis, so unlikely. |
| Other sources of bias | | High | Study based on statewide prevalence rates and exposures—this is likely to be a biased evaluation of the relationship. |

^*^ All modeled HAPS considered

**Table G. Characteristics of Blanchard et al. 2011 [8]**

| Study Element | Description |
| --- | --- |
| Design | Ecological study |
| Participants | Study population was students in Bexar County, TX (all ages) and Santa Clara County, CA (elementary school ages) in 2008. Study group was not reported. |
| Exposure | Block-level estimates of ambient mercury levels from the 2002 US EPA NATA database mapped to school district. |
| Comparator | City blocks in the counties with lower estimated mercury emissions compared to those with higher estimated mercury emissions . |
| Outcomes | Autism rates obtained from the Texas Education Association for Texas cases and from <http://www.kidsdata.org> for California cases. |

| Bias domain | Authors’ judgment | | Support for judgment |
| --- | --- | --- | --- |
| Source population representation | High | | No information provided about the selection of the counties evaluated in the study, and no information provided about the characteristics of the counties and school districts. No individual level data were collected. |
| Blinding | | Probably low | Authors do not address blinding, but case status and exposure assessment performed independently, minimizing bias. |
| Exposure assessment | | High^*^ | Judgment supported by 1996 US EPA National Air Toxics Assessment Table A: Composite judgments to determine overall certainty. |
| Outcome assessment | | Probably high | Autism rates were calculated based on state reported diagnoses within school districts; no information provided on how assessments were made or on the validity of the assessments. |
| Confounding | | High | No confounders accounted for. |
| Incomplete outcome data | | Probably high | No information provided to assess incomplete outcome data. |
| Selective outcome reporting | | Probably low | No information provided to support comparing association between mercury and autism between counties. |
| Conflict of interest | | Low | No COI statement, but no evidence to support risk of bias. |
| Other sources of bias | | High | Ecologic study introduces bias that cannot be fully captured by other domains. |

^*^ Mercury compounds

**Table H. Characteristics of Volk et al. 2011 [9]**

| Study Element | Description |
| --- | --- |
| Design | Case-control study |
| Participants | Study population was children enrolled in the CHARGE study and born between 1997-2006 in California . Study group was 304 children with autism and 259 typically developing controls. |
| Exposure | Distance to freeways and major roads from mother’s addresses throughout pregnancy and at birth used as a proxy for traffic-related pollutant exposure. |
| Comparator | Typically developing general-population from CHARGE study. |
| Outcomes | Autism cases recruited by California DDS and children evaluated and diagnosed using the ADI-R and ADOS tools; controls were selected based on SCQ. |

| Bias domain | Authors’ judgment | | Support for judgment |
| --- | --- | --- | --- |
| Source population representation | Probably low | | CHARGE study uses DDS recruitment, and cases and controls have similar characteristics and description of the case population and enrollment procedures are sufficiently detailed. However, there is limited information on the control population, as well as sampling and participation rates. |
| Blinding | | Probably low | No acknowledgment of blinding. As exposure assessment was added to an existing study, assume it was assigned without knowing case status. |
| Exposure assessment | | Probably high^*^ | Overall exposure assessment is crude; surrogate with no temporal variation. No dose response and odd that fairly distant from freeway shows risk but not much closer to major roadway. |
| Outcome assessment | | Low | In-person evaluation using ADOS/ADI-R, with controls evaluated using SCQ. Robust method of assessing outcome. |
| Confounding | | Probably high | Authors frequency-matched for gender, age and region. Although some important cofounders were considered, authors did not address season, year or urbanicity. |
| Incomplete outcome data | | Probably low | Not a cohort study but children were 24‐60 months old so controls could still be diagnosed. No acknowledgment of response or completion rates. |
| Selective outcome reporting | | Low | No evidence of selective outcome reporting. |
| Conflict of interest | | Probably low | One study author worked for a private company that provides closure assessment, but no financial interests declared. |
| Other sources of bias | | Low | No other potential sources of bias identified. |

^*^ Distance to freeway, distance to major road

**Table I. Characteristics of McCanlies et al. 2012 [10]**

| Study Element | Description |
| --- | --- |
| Design | Case-control study |
| Participants | Study population was children enrolled in the CHARGE study and born between 1998-2003 in California. Study group was 93 children with ASD and 81 unaffected controls. |
| Exposure | Self-reported and industrial hygenist-assessed parental occupational exposures to 49 chemical agents for the time period 3 months prior to pregnancy through either birth of child or until weaning if child was breastfed. |
|  | Children in CHARGE study that were categorized as typical development (TD). |
| Outcomes | ASD (combined autism and ASD) cases recruited by California DDS and children evaluated and diagnosed using the ADI-R and ADOS tools; controls were selected based on SCQ. |

| Bias domain | Authors’ judgment | | Support for judgment |
| --- | --- | --- | --- |
| Source population representation | Probably low | | CHARGE study uses DDS recruitment, and cases and controls have similar characteristics and description of the population and enrollment procedures are sufficiently detailed. However, there is limited information on non-respondents and a low control participation rate. |
| Blinding | | Low | Authors explicitly state that exposure assessment was blinded to outcome. |
| Exposure assessment | | High^*^ | Exposure assessed using surrogate, indirect measure of “occupational exposure”. |
| Outcome assessment | | Low | Consistency and accuracy of case definition, as well as ascertainment procedures, were robust (two standardized instruments used by well-trained staff to diagnose). |
| Confounding | | Probably high | Controls for confounders through matching, which may lead to potential bias. |
| Incomplete outcome data | | Probably low | No missing outcome data noted, but methods could result in missing some individuals. |
| Selective outcome reporting | | Low | All of the study’s pre‐specified (primary and secondary) outcomes outlined in the protocol, methods, abstract, and/or introduction that are of interest in the review were reported in the pre‐specified way. |
| Conflict of interest | | Low | Funding provided by National Institutes of Health, U.S. Environmental Protection Agency through the Science to Achieve Results (STAR) program. No indication that there are financial conflicts of interest. |
| Other sources of bias | | Low | No other potential sources of bias identified. |

^*^asphalt, ethylene oxide, lead, mercury, nickel, chromium, metal dust fumes, pain/lacquer/varnish, solvents

**Table J. Characteristics of Becerra et al. 2013 [11]**

| Study Element | Description |
| --- | --- |
| Design | Case-control study |
| Participants | Study population was children born in 1994-2006 in Los Angeles County. Study group was 7,603 children with autism and 75,782 controls. |
| Exposure | Birth address linked to measurements for CO, NO2, NO, O3, PM10, and PM2 obtained from nearest monitoring stations and exposures averaged for periods of pregnancy. |
| Comparator | Controls from California birth certificates matched by case, birth year, and minimum gestational age in a 10:1 ratio. |
| Outcomes | Autistic disorder cases identified from records-based surveillance of children conducted by California DDS. |

| Bias domain | Authors’ judgment | | Support for judgment |
| --- | --- | --- | --- |
| Source population representation | Probably low | | No evidence of selection bias. Some mismatch in case numbers and cases more severe so not completely representative. Small percent of cases could not be linked to birth certificate and controls matched to cases, so not representative of unaffected population per se. |
| Blinding | | Probably low | Authors do not address blinding, but case status and exposure assessment performed independently, minimizing bias. |
| Exposure assessment | | Low^*^  Probably low^+^  Probably high^#^ | NO and NO_2_ monitoring data more likely to be driven by local sources such as roadways when compared to PM. PM_10_ and PM_2.5_ monitoring data should be treated similarly; monitoring network in the L.A. area is reasonably dense, which means less bias for these measurements. Seasonalized estimates are adjusted for seasons, which is a potential confounder. Model/monitor comparisons show that modeling can explain a reasonably high percentage of variation. |
| Outcome assessment | | Probably low | Used California Department of Developmental Services database. Dichotomous outcome based on community diagnosis documented in medical records, educational records, or a health registry, but no record review to verify and eligibility can vary by regional center. |
| Confounding | | Low | Most relevant confounders considered. No adjustment for seasonality, but authors considered this in their model. No adjustment for urbanicity, but study focuses on LA county (limited rural area). |
| Incomplete outcome data | | Probably high | Some mismatch in case numbers between methods and tables; cases were young and may include children not yet diagnosed with autism. About 6% of cases were not matched to birth certificate. |
| Selective outcome reporting | | Low | No evidence of reporting bias. |
| Conflict of interest | | Low | Authors declare no financial conflicts of interest, and no reason to suspect otherwise. |
| Other sources of bias | | Low | No other potential sources of bias identified. |

^*^seasonalized-land use regression modeled- nitrogen oxide, seasonalized-land use regression modeled-nitrogen dioxide

^+^ozone, PM_10_, PM_2.5_

^#^carbon monoxide, nitrogen oxide, nitrogen dioxide, land use regression modeled-nitrogen oxide, land use regression modeled-nitrogen dioxide

**Table K. Characteristics of Jung et al. 2013 [12]**

| Study Element | Description |
| --- | --- |
| Design | Cohort study |
| Participants | Study population was children aged less than 3 years in 2000 enrolled in prospective cohort study in Taiwan. Study group was 342 children with ASD and 48,731 non-ASD controls. |
| Exposure | Residence postal code linked to measurements for CO, NO2, SO2, PM10, and O3 from three nearest monitoring stations and averaged yearly for 1-4 years preceding diagnosis. |
| Comparator | Controls from longitudinal health insurance database not categorized as ASD cases. |
| Outcomes | ASD and non-ASD children in cohort identified based on diagnosis codes provided in the Taiwan National Insurance Research Database. |

| Bias domain | Authors’ judgment | | Support for judgment |
| --- | --- | --- | --- |
| Source population representation | Low | | Sufficient information provided regarding study selection and study population so that representation is not likely a concern. |
| Blinding | | Probably low | The study outcome was records based so the researchers had no part in the outcome assessment. The exposure assessment was modeled on data that the researchers had no part in collecting. |
| Exposure assessment | | Probably low^*^ | Although model uses methods that do not include time-activity patterns and spatial accuracy, they do include measurements that have evidence of good quality. |
| Outcome assessment | | Low | To ensure the diagnostic validity of ASD in present study, only children with at least two consensus diagnoses of autistic disorder, Asperger syndrome, or PDD‐NOS included. |
| Confounding | | Probably high | Controlled for parental age, gender, SES, and several ASD comorbidities. Used statistical analyses to identify statistically important confounders and also selected potential confounders a priori to include in the model. |
| Incomplete outcome data | | Probably low | No missing outcome data noted, but methods could result in missing some individuals. |
| Selective outcome reporting | | Low | All of the study’s pre‐specified (primary and secondary) outcomes outlined in the protocol, methods, abstract, and/or introduction that are of interest in the review were reported in the pre‐specified way. |
| Conflict of interest | | Low | The funders had no role in study design, data collection and analysis, decision to publish, or preparation of the manuscript. The authors have declared that no competing interests exist. |
| Other sources of bias | | Low | No other potential sources of bias identified. |

^*^carbon monoxide, nitrogen dioxide, sulfur dioxide, ozone, PM_10_

**Table L. Characteristics of Pino-Lopez and Romero-Ayuso 2013 [13]**

| Study Element | Description |
| --- | --- |
| Design | Case-control study |
| Participants | Study population was children aged 12-36 months evaluated by the Early Intervention Service between January 2009 and February 2011. Study group was 70 children with ASD and 136 unaffected controls. |
| Exposure | Self-reported parental occupation compared to NIOSH criteria to evaluate exposure to solvents. |
| Comparator | Controls from Early Intervention Service not categorized as Autism cases. |
| Outcomes | ASD cases and unaffected controls identified through the Early Intervention Service of Ciudad Real. |

| Bias domain | Authors’ judgment | | Support for judgment |
| --- | --- | --- | --- |
| Source population representation | Low | | All children who came to the Early Intervention Service during the period of study (January 1, 2009 and February 28, 2011) and who were between 16 and 36 months were equally eligible for inclusion in the study population. Exposure and outcome were ascertained in the same way among cases and controls. |
| Blinding | | Low | Authors explicitly state that two scientists independently coded for occupation, i.e., the exposure surrogate, and that both were blinded to the outcome assessment results when coding for exposure. The outcomes were assessed the same way for the cases and controls. |
| Exposure assessment | | High^*^ | “Air pollution” exposure was assessed using a surrogate, indirect measure of “occupational exposure to solvents” as follows: parent’s (both mothers and fathers) “current occupation” at the time the child was seen at the Early Intervention Service, which was when the child was 16 to 36 months of age. If the parent was unemployed they were asked for their “last occupation.” It can be assumed that there would be little to no recall bias in an individual knowing/reporting her or his current or last occupation. The timing of the exposure assessment preceded the outcome measurement, and occurred during a developmentally important period. However we do not know if parental exposure when the child is 16 to 36 months of age reflects peri or pre-natal exposure, which may be more relevant to a health outcome at 16 to 36 months. Above all, the relationship between “occupation” and “air pollution exposure” is very tenuous and likely to introduce bias into the exposure assessment. The “air pollutant” assessed in this study is a surrogate. |
| Outcome assessment | | Probably high | Outcome assessed via tool developed as autism risk screener i.e., MCHAT. Not a commonly used metric. |
| Confounding | | Probably low | The study controls for parental age and education. They excluded 5 children who were not the biological children of the parent’s, i.e., foster or adopted children. The authors note that all of the cases and controls are being seen at the Early Intervention Service, which makes the cases and controls equivalent for many social factors, including access to care and social class, education and income of the parents, and that this overcomes a shortcoming of many studies of ASD in which lower SES children are under‐ represented. They do not control for season of birth. There is potential uncontrolled for confounding in the exposure assessment, i.e., most of the “solvent exposed” workers were “persons who cleaned offices, hotels, and other similar establishments.” While it is a reasonable assumption that housekeeping personnel have exposure to cleaning products with solvents, it is also reasonable that [some of] these workers also have exposure to “night work” (also evaluated by the study) at the same time they had (or did not have) exposure to solvents in cleaning products. |
| Incomplete outcome data | | Low | No missing outcome data noted. |
| Selective outcome reporting | | Low | All of the study’s pre‐specified (primary and secondary) outcomes, i.e., ASD, outlined in the protocol, methods, abstract, and/or introduction that are of interest in the review were reported in the pre‐specified way. |
| Conflict of interest | | Low | The authors explicitly stated “No conflicts of interest exist;” the first author is from what appears to be a governmental health (city/municipality) organization and the second author is academic faculty. |
| Other sources of bias | | Probably high | The “source population” itself is not representative of the “general population” –all cases and controls were either self‐ referred by the parent, or more usually, referred to the Early Intervention Service by an educational, health, or social services provider for a  developmental delay of some kind. |

^*^parent’s occupation

**Table M. Characteristics of Volk et al. 2013 [14]**

| Study Element | Description |
| --- | --- |
| Design | Case-control study |
| Participants | Study population was children enrolled in the CHARGE study and born between 1997-2006 in California. Study group was 279 children with autism and 245 typically developing controls. |
| Exposure | Mother’s addresses throughout pregnancy and at birth linked to 1) CALINE4 line-source air quality dispersion model to estimate exposure to traffic-related air pollution for 1997-2008 and 2) regional air quality data for PM2.5, PM10, ozone and NO2 using US EPA AQS data and USC Children’s Health Study data for 1997-2009. |
| Comparator | Population-based control children recruited from sampling frame of birth files from CA and were frequency matched by sex, age, and broad geographic area to cases. |
| Outcomes | Autism cases recruited by California DDS and children evaluated and diagnosed using the ADI-R and ADOS tools; controls were selected based on SCQ. |

| Bias domain | Authors’ judgment | | Support for judgment |
| --- | --- | --- | --- |
| Source population representation | Probably low | | The data come from the CHARGE study, and the source population is thoroughly described, inclusion/exclusion criteria, procedures, etc. are provided, and population characteristics are included, all supporting the judgment that risk of selection effects are minimal. However, there is limited information on the control population, as well as sampling and participation rates. |
| Blinding | | Probably low | Although no mention of blinding, exposures were determined based on address and computer model for air pollution, which should minimize likelihood that there is a risk of bias due to lack of blinding. |
| Exposure assessment | | Probably low^*^ | Used monthly air quality data from US EPA Air Quality System monitoring stations located within 50 km of each residence. Based on address at time of birth and residential history questionnaire. No accounting for time-activity. For traffic-related air pollution, used CALINE4 line-source air quality dispersion model--appears to be a validated model. Used to estimate exposure for trimesters and first year of life based on child's residence. Includes data on mixing height, meteorological data, etc. Estimates based on concentrations of primary pollutants contributed by roads within a 5 km radius of each child's home. Based on address at time of birth and residential history questionnaire. No accounting for time-activity. Because models did not meet the criteria of including time-activity patterns and spatial accuracy, but do include measurements that have evidence of quality and the model appears robust with reasonably accurate data inputs, the judgment was "probably low risk" of bias. |
| Outcome assessment | | Low | In-person evaluation using ADOS/ADI-R, with controls evaluated using SCQ. Robust method of assessing outcome. |
| Confounding | | Probably low | The study adjusted for some of the potentially important cofounders as follows: 1) social class ‐ adjusted for maximum parental education; 2) urban residence ‐ adjusted for population density; 3) maternal (and paternal) age ‐ adjusted for maternal age, which was judged to be sufficient. The "probably low risk" of bias rating is due to a lack of adjustment for 1) season of conception/birth ‐ because air pollution data is reported based on trimester, this could be a potential source  of confounding; and 2) calendar time ‐ the study takes place over a long time period (1997‐2008) and does not adjust calendar time as a potential confounder. However, controls are frequency matched to cases based on age, so this could help to reduce potential bias for this confounder. |
| Incomplete outcome data | | Probably low | No missing outcome data noted, but methods could result in missing some individuals. |
| Selective outcome reporting | | Low | All pre-specified outcomes were reported in the pre-specified way. |
| Conflict of interest | | Probably low | One author (Rob McConnell) received support from an air quality air violations settlement agreement between a California State regulatory agency and BP. The grant is administered by the State regulatory agency. Although no information is provided about any conditions associated with this support, it is unlikely that the initial source of funds, BP, presents a conflict of interest with respect to the author's work on this study. Also, two authors are employed by a company (Sonoma Technology, Inc.); however, this does not appear to present a financial conflict of interest for this study. |
| Other sources of bias | | Low | No other potential sources of bias identified. |

^*^PM_2.5_, PM_10_, nitrogen dioxide, ozone, traffic-related air pollution

**Table N. Characteristics of Windham et al. 2013 [15]**

| Study Element | Description |
| --- | --- |
| Design | Case-control study |
| Participants | Study population was children born in 1994 in 6 counties in the San Francisco Bay area. Study group was 284 children with ASD and 659 controls. |
| Exposure | Self-reported parental occupation recorded on birth certificates were coded by occupational medicine-certified physician identify exposures to chemicals organized into 7 broad categories. |
| Comparator | Controls from same area randomly selected from CA’s 1994 linked birth-infant death files, matched in 2:1 ratio marched by genera and month of birth to cases. |
| Outcomes | ASD cases identified from records-based surveillance of children conducted by California CADDRE. |

| Bias domain | Authors’ judgment | | Support for judgment |
| --- | --- | --- | --- |
| Source population representation | Probably low | | The source and control populations were clearly described, as well as procedures, inclusion/exclusion criteria, etc. The paper includes a table with relevant population characteristics. Population is the same as for Windham 2006, and the paper refers to this paper for additional details. The details provided in Windham 2006 appear to support the summary found in this paper. |
| Blinding | | Low | Authors specifically state that study was blinded. |
| Exposure assessment | | High^*^ | Exposure assessed using surrogate, indirect measure of “occupational exposure”. |
| Outcome assessment | | Probably low | Some classifications were "low risk of bias" such as diagnosis of ASD from a qualified medical professional and behaviors meeting DSM‐IV. However, one classification was "qualification for special education under an autism exceptionality" and no criteria for this classification were described. |
| Confounding | | Probably low | Important confounders were listed, but not necessarily incorporated into the analysis. No mention of residential exposure. |
| Incomplete outcome data | | Probably low | No missing outcome data noted, but methods could result in missing some individuals. |
| Selective outcome reporting | | Low | All pre-specified outcomes were reported in the pre-specified way. |
| Conflict of interest | | Low | Authors declare no competing financial interests. |
| Other sources of bias | | Low | No other potential sources of bias identified. |

^*^exhaust, solvents, pesticides, metals

**Table O. Characteristics of Gong et al. 2014 [16]**

| Study Element | Description |
| --- | --- |
| Design | Case-control study |
| Participants | Study population was twins born after July 1, 1992 and enrolled in the CATSS longitudinal study. Study group was 109 children with ASD and 3,051 healthy controls. |
| Exposure | Mother’s addresses during pregnancy, child’s first year of life, and the year before assessment were linked to historical emission databases to estimate exposures to NOx and PM10. |
| Comparator | Cases from CATSS longitudinal study not categorized as Autism cases. |
| Outcomes | ASD cases and controls identified after assessment using A-TAC tool at 9 and 12 years of age conducted by the CATSS. |

| Bias domain | Authors’ judgment | | Support for judgment |
| --- | --- | --- | --- |
| Source population representation | Probably low | | Minimal selection bias, only concern about using twins for study, but unlikely to introduce bias in this case. |
| Blinding | | Probably low | Authors do not address blinding, but case status and exposure assessment performed independently, minimizing bias. |
| Exposure assessment | | Probably low^*^ | Historical emissions database, reconstructed emissions from dispersion model. Minimal bias anticipated. |
| Outcome assessment | | Probably low | Used Autism‐Tics, ADHD, and other Comorbidities inventory (A‐TAC) phone interviews, which is not a common instrument, but is based on DSM‐IV criteria. |
| Confounding | | Probably low | Most important cofounders considered, but seasonality and urban residence not addressed. |
| Incomplete outcome data | | Probably high | 1/3 of twin cohort did not respond, and this was related to SES, and a small percent of cases without autism data were excluded. |
| Selective outcome reporting | | Probably low | Authors propose evaluating other developmental disabilities (comorbidities), but did not report on these results. |
| Conflict of interest | | Low | No COI statement, but no evidence to support risk of bias. |
| Other sources of bias | | Probably low | Reviewers note issues with small number of affected children despite relatively high affected rate and potential correlation due to genetic basis for autism. |

^*^nitrogen oxides, PM_10_

**Table P. Characteristics of Kalkbrenner et al. 2015 [17]**

| Study Element | Description |
| --- | --- |
| Design | Case-control study |
| Participants | Study population was Children born in North Carolina in 1994 (8 counties), 1996 (8 counties), 1998 (9 counties), and 2000 (10 counties) and born in 6 San Francisco Bay area counties in 1996. Study group was 645 children with autism and 12,434 controls for North Carolina and 334 children with autism and 2,232 controls for California. |
| Exposure | Child’s birth address linked to PM10 levels from the US EPA AQS database starting 1 year before child’s birth to 1 year following birth. |
| Comparator | Controls randomly selected from study counties and birth years, removing multiple births, infant deaths, and known autism cases. |
| Outcomes | Autism cases identified from records-based surveillance of children conducted by the ADDM in North Carolina and California. |

| Bias domain | Authors’ judgment | | Support for judgment |
| --- | --- | --- | --- |
| Source population representation | Low | | Authors do not go into great detail about population, but ADDM methodology should be representative as these methods are robust and well‐described. Unlikely that selection based on outcome would be directly related to exposure, though there could be an association with exposure (e.g. SES). |
| Blinding | | Probably low | Although no mention of blinding, exposures were determined based on geocoded address and estimated using modeling of US EPA Air Quality System data from regulatory monitors, which should minimize likelihood that there is a risk of bias due to lack of blinding. |
| Exposure assessment | | Probably low^*^ | Model based on measurements from US EPA Air Quality System. Although the model does not account for temporal specificity, time and activity patterns, or meterological variables, the model does include sensitivity analyses and appears robust with reasonable accurate data inputs indicating that it is of good quality. |
| Outcome assessment | | Probably low | Case definition used ADDM methods, relying on developmental records documenting characteristics and behaviors meeting a standardized definition of autism spectrum disorder based on DSM‐IV‐TR. Although no validation by direct clinical assessment was performed in this study, prior validation of ADDM records‐based diagnoses indicate good agreement with ASD diagnostic instruments. |
| Confounding | | Low | The study adjusted for each of the potentially important cofounders as follows: 1) social class ‐ adjusted for maternal education; 2) urban residence ‐ adjusted for 2000 Census block group urbanization; 3) maternal (and paternal) age ‐ adjusted for maternal age; 4) season of conception/birth ‐ adjusted for calendar week of birth; 5) calendar time ‐ all cases and controls were births occurring between 1994‐2000 for NC and in 1996 for CA. |
| Incomplete outcome data | | Probably low | No missing outcome data noted, but methods could result in missing some individuals. |
| Selective outcome reporting | | Low | All pre‐specified outcomes were reported in the pre‐specified way. |
| Conflict of interest | | Low | The study does not have a statement regarding conflicts of interest; however, all funding was from the NIH, and the authors are affiliated with academic universities and the CDC, suggesting that it was not supported by any entities with a financial interest in the outcome. |
| Other sources of bias | | Low | No other potential sources of bias identified. |

^*^PM_10_

**Table Q. Characteristics of Roberts et al. 2013 [18]**

| Study Element | Description |
| --- | --- |
| Design | Case-control study |
| Participants | Study population was children of Nurses’ Health Study II participants born between 1987-2002. Study group was 325 children with ASD and 22,098 controls. |
| Exposure | Census tract of mailing address for study questionnaire at approximate time of child’s birth linked to concentration estimates from the 1990, 1996, 1999, and 2002 US EPA NATA database for 14 HAPs. |
| Comparator | Controls were children of Nurses’ Health Study II participants born during 1987-2002 where parents indicated they never had a child with ASD. |
| Outcomes | ASD cases identified based on Nurses’ Health Study II participant’s response to questionnaire, and validated by administration of the ADI-R to a random subset of case mothers. |

| Bias domain | Authors’ judgment | | Support for judgment |
| --- | --- | --- | --- |
| Source population representation | Probably low | | Descriptions of source population, inclusion/exclusion criteria, recruitment and enrollment procedures, participation, and follow‐up rates sufficiently detailed. |
| Blinding | | Probably low | Exposure was assigned after cases/controls identified. |
| Exposure assessment | | Probably low^*^  Probably high^+^  High^#^ | Judgment supported by 1996 US EPA National Air Toxics Assessment Table A: Composite judgments to determine overall certainty. |
| Outcome assessment | | Probably low | A subgroup analysis validated parent reported diagnosis, and criteria reported in paper. |
| Confounding | | Probably low | Controlled for social class, year  (calendar time), maternal age. Season not likely to influence because air pollution exposures are averages over a year. Adjusted urbanicity in subanalyses of metals metric. |
| Incomplete outcome data | | Probably low | No missing outcome data noted, but methods could result in missing some individuals. |
| Selective outcome reporting | | Low | No evidence of selective outcome reporting. |
| Conflict of interest | | Low | No evidence of financial conflict of interest. |
| Other sources of bias | | Low | No other potential sources of bias identified. |

^*^methylene chloride, styrene, trichloroethylene

^+^diesel particulate, vinyl chloride

^#^quinoline, overall metals, antimony, arsenic, cadmium, chromium, lead, manganese, mercury, nickel

**Table R. Characteristics of Shelton et al. 2014 (**[**Shelton et al. 2014**](#_ENREF_52)**)**

| Study Element | Description |
| --- | --- |
| Design | Case-control study |
| Participants | Study population was children enrolled in the CHARGE study and born after 2003. Study group was 486 children with ASD and 315 typically developing children as controls. |
| Exposure | Residential addresses from 3 months before conception to birth linked to California DPR database to determine proximity to application of 4 classes of pesticides between 1997-2008. |
| Comparator | Controls were children enrolled in CHARGE not categorized as Autism cases. |
| Outcomes | Autism cases recruited by California DDS and children evaluated and diagnosed using the ADI-R and ADOS tools; controls were selected based on SCQ. |

| Bias domain | Authors’ judgment | | Support for judgment |
| --- | --- | --- | --- |
| Source population representation | Probably low | | CHARGE study uses DDS recruitment, and cases and controls have similar characteristics and description of the case population and enrollment procedures are sufficiently detailed. However, there is limited information on the control population, as well as sampling and participation rates. |
| Blinding | | Probably low | Authors do not address blinding, but case status and exposure assessment performed independently, minimizing bias. |
| Exposure assessment | | Probably low^*^ | Spatial resolution around 1km. |
| Outcome assessment | | Low | ADOS/ADI confirmed, which is an adequately robust method for assessing outcome and sufficient for minimizing bias. |
| Confounding | | Probably low | Nearly all important factors were considered, except for urban residence, which seems adequate for minimizing confounding bias. |
| Incomplete outcome data | | Probably low | No missing outcome data noted, but methods could result in missing some individuals. |
| Selective outcome reporting | | Low | No evidence of selective outcome reporting. |
| Conflict of interest | | Low | No evidence of financial conflict of interest. |
| Other sources of bias | | Low | No other potential sources of bias identified. |

^*^organophosphates, chlorpyrifos, pyrethroids, type 2 pyrethroids, carbamates

**Table S. Characteristics of Volk et al. 2014 [19]**

| Study Element | Description |
| --- | --- |
| Design | Case-control study |
| Participants | Study population was children enrolled in the CHARGE study. Study group was 251 children with ASD and 156 controls. |
| Exposure | Mother’s addresses throughout pregnancy, at birth, and any place of residence for child were linked to 1) CALINE4 line-source air quality dispersion model to estimate exposure to traffic-related air pollution and 2) regional air quality data for PM2.5, PM10, ozone and NO2 using US EPA AQS data and USC Children’s Health Study data for 1997-2009. |
| Comparator | Controls selected from CHARGE study that were defined as typically developing as assessed by Social Communication Questionnaire and Millen Scales of Early Learning and Vineland Adaptive Behavior Scales. |
| Outcomes | ASD cases recruited by California DDS and children evaluated and diagnosed using the ADI-R and ADOS tools; controls were selected based on SCQ. |

| Bias domain | Authors’ judgment | | Support for judgment |
| --- | --- | --- | --- |
| Source population representation | Probably low | | The reliance on DDS for identifying autism cases will not include milder cases and/or those from autism families with higher SES who may not need to access services through DDS. Enrollment rates were lower from families with normally developing children compared to those families with autism and there were demographic differences between cases and controls. Finally, CHARGE participants who agreed to be genotyped differed somewhat from those who declined. |
| Blinding | | Probably low | No mention of blinding (other than for genotyping), but exposure imputation methods and diagnostic assessments are not likely to be influenced by lack of blinding. |
| Exposure assessment | | Probably low^*^ | Used monthly air quality data from US EPA Air Quality System monitoring stations located within 50 km of each residence. Based on address at time of birth and residential history questionnaire. No accounting for time-activity. For traffic-related air pollution, used CALINE4 line-source air quality dispersion model--appears to be a validated model. Used to estimate exposure for trimesters and first year of life based on child's residence. Includes data on mixing height, meteorological data, etc. Estimates based on concentrations of primary pollutants contributed by roads within a 5 km radius of each child's home. Based on address at time of birth and residential history questionnaire. No accounting for time-activity. Because models did not meet the criteria of including time-activity patterns and spatial accuracy, but do include measurements that have evidence of quality and the model appears robust with reasonably accurate data inputs, the judgment was "probably low risk" of bias. |
| Outcome assessment | | Low | Autism cases were based on clinical administration of ADOS and ADI. Normally developing children were screened using SCQ. These methods constitute a robust outcome assessment. |
| Confounding | | Probably high | Analyses were adjusted for child's sex and ethnicity, maximum education level in the home, maternal age, home ownership and prenatal smoking. Differences in demographics of cases and controls suggest there may be other unknown factors that could act as confounders. No information was provided regarding matching of CHARGE cases and controls that contributed to this particular study for local residential area  (e.g., urbanicity) and season of birth. |
| Incomplete outcome data | | Probably low | No missing outcome data noted, but methods could result in missing some individuals. |
| Selective outcome reporting | | Low | Pre-specified outcomes were reported in the pre-specified way. |
| Conflict of interest | | Probably low | One author (Rob McConnell) received support from an air quality air violations settlement agreement between a California State regulatory agency and BP. The grant is administered by the State regulatory agency. Although no information is provided about any conditions associated with this support, it is unlikely that the initial source of funds, BP, presents a conflict of interest with respect to the author's work on this study. |
| Other sources of bias | | Low | No other potential sources of bias. |

^*^traffic related air pollution, PM_2.5_, PM_10_, nitrogen dioxide, ozone

**Table T. Characteristics of von Ehrenstein et al. 2014 [20]**

| Study Element | Description |
| --- | --- |
| Design | Case-control study |
| Participants | Study population was children born between 1995-2006 in Los Angeles County. Study group was 768 children with autism and 147,954 controls. |
| Exposure | Birth address linked to measurements for 24 air pollutants obtained from air-monitoring stations within a 5-km radius and averaged for periods of pregnancy. |
| Comparator | Controls were those within LA County birth records not categorizes as Autism case. |
| Outcomes | Autism cases identified from records-based surveillance of children conducted by California DDS. |

| Bias domain | Authors’ judgment | | Support for judgment |
| --- | --- | --- | --- |
| Source population representation | Probably low | | Generally, sufficient information provided regarding study selection and study population. All births in a given area that could be geocoded were included. However, there is no full explanation for how numbers were narrowed down to the final numbers. |
| Blinding | | Probably low | Not explicitly discussed, but no way for knowledge of outcome status to influence exposure value or vice versa. |
| Exposure assessment | | Probably low^*^ | Used monthly air quality data from US EPA Air Quality System monitoring stations located within 50 km of each residence. Based on inverse distance-squared weighting of data from 4 closest stations and address at time of birth and residential history questionnaire. No accounting for time-activity. Because model did not meet the criteria of including time-activity patterns and spatial accuracy, but do include measurements that have evidence of quality and the model appears robust with reasonably accurate data inputs, the judgment was "probably low risk" of bias. |
| Outcome assessment | | Probably low | Based on records from California DDS. Diagnoses are based on DSM IV-R. Prior research demonstrates good diagnostic validity of DDS-identified autism cases. |
| Confounding | | Probably high | Although controlled for some confounding factors, did not control for urbanicity, season of conception, or calendar year. |
| Incomplete outcome data | | Probably low | No missing outcome data noted, but methods could result in missing some individuals. |
| Selective outcome reporting | | Low | All pre-specified outcomes reported in the pre-specified way. |
| Conflict of interest | | Low | No evidence of financial conflict of interest. |
| Other sources of bias | | Low | No other potential sources of bias. |

^*^benzene, perchloroethylene, 1,3-butadiene, toluene, ortho-xylene, meta/para-xylene, ethylbenzene, methylene chloride, polyaromatic hydrocarbons, lead, vanadium, chromium, manganese, nickel, selenium, acetaldehyde, formaldehyde, ortho-dichlorobenzene, para-dichlorobenzene, chloroform, trichloroethylene, copper, hexavalent chromium, molybdenum

**Table U. Characteristics of Raz et al. 2014 [21]**

| Study Element | Description |
| --- | --- |
| Design | Case-control study |
| Participants | Study population was children of Nurses’ Health Study II participants born between 1990-2002. Study group was 245 children with ASD and 1,522 controls. |
| Exposure | Mailing address for study questionnaire at approximate time of child’s birth linked to concentration estimates of PM10 and PM2.5 obtained from US EPA AQS and averaged for periods of pregnancy. |
| Comparator | Controls selected from parous women not reporting a child with ASD in 2005, matched to cases by birth year. |
| Outcomes | ASD cases identified based on Nurses’ Health Study II participant’s response to questionnaire, and validated by administration of the ADI-R to a random subset of case mothers. |

| Bias domain | Authors’ judgment | | Support for judgment |
| --- | --- | --- | --- |
| Source population representation | Probably low | | Descriptions of source population, inclusion/exclusion criteria, recruitment and enrollment procedures, participation, and follow‐up rates sufficiently detailed. |
| Blinding | | Probably low | Exposure was assigned after cases/controls identified. |
| Exposure assessment | | Probably low^*^ | Modeling included spatial accuracy, distance, population density, point sources, and meteorology. |
| Outcome assessment | | Probably low | A subgroup analysis validated parent reported diagnosis, and criteria reported in paper. |
| Confounding | | Probably low | Controlled for most potential confounding factors, other than season of birth and urbanicity. |
| Incomplete outcome data | | Probably low | No missing outcome data noted, but methods could result in missing some individuals. |
| Selective outcome reporting | | Low | No evidence of selective outcome reporting. |
| Conflict of interest | | Low | No evidence of financial conflict of interest. |
| Other sources of bias | | Low | No other potential sources of bias identified. |

^*^PM_10_, PM_2.3_

**Table V. Characteristics of Dickerson et al. 2015 [22]**

| Study Element | Description |
| --- | --- |
| Design | Ecological study |
| Participants | Study population was children 8 years of age in 2000, 2002, 2004, 2006 and 2008 from Arizona, Maryland, New Jersey, South Carolina, and Utah. Study group was 4,486 children with ASD. |
| Exposure | Census tracts from ADDM surveillance areas linked to US EPA TRI data from 1991-1999 obtained for air releases of arsenic, lead, and mercury from facilities in closest proximity. |
| Comparator | Comparator was children exposed to lower levels of air pollutant that more highly exposed. |
| Outcomes | ASD cases identified from records-based surveillance of children conducted by ADDM network. |

| Bias domain | Authors’ judgment | | Support for judgment |
| --- | --- | --- | --- |
| Source population representation | Low | | Authors do not go into great detail about population, but ADDM methodology should be representative as these methods are robust and well‐described. Unlikely that selection based on outcome would be directly related to exposure, though there could be an association with exposure (e.g. SES). |
| Blinding | | Probably low | Although no mention of blinding, exposures were determined based on address and proximity to industrial facilities using computer modeling, which should minimize likelihood that there is a risk of bias due to lack of blinding. |
| Exposure assessment | | High^*^ | US EPA toxic release inventory data used, which may not result in estimating true exposure. Not based on per person measurements and no accounting for variability due to differences in release between sites. |
| Outcome assessment | | Probably low | Case definition used ADDM methods, relying on developmental records documenting characteristics and behaviors meeting a standardized definition of autism spectrum disorder based on DSM‐IV‐TR. Although no validation by direct clinical assessment was performed in this study, prior validation of ADDM records‐based diagnoses indicate good agreement with ASD diagnostic instruments. |
| Confounding | | Probably high | Most potentially important confounding variables were not accounted for in the analysis. Also, cases were aggregated for the past five years, which is a problem considering prevalence changes over time. |
| Incomplete outcome data | | Probably low | No missing outcome data noted, but methods could result in missing some individuals. |
| Selective outcome reporting | | Low | All pre-specified outcomes were reported in the pre-specified way. |
| Conflict of interest | | Low | The study does not have a statement regarding conflicts of interest; however, all funding was from the NIH, and the authors are affiliated with academic universities and the CDC, suggesting that it was not supported by any entities with a financial interest in the outcome. |
| Other sources of bias | | Low | No other potential sources of bias. |

^*^arsenic, lead, mercury

**Table W. Characteristics of Dickerson et al. 2016 [23]**

| Study Element | Description |
| --- | --- |
| Design | Ecological study |
| Participants | Study population was Children 8 years of age in 2000, 2002, 2004, 2006 and 2008 from Arizona, Maryland, New Jersey, South Carolina, and Utah. Study group was 4,486 children with ASD. |
| Exposure | Census tracts from ADDM surveillance areas linked to concentration estimates from the 1999 US EPA NATA database for inorganic arsenic, lead, and mercury compounds. |
| Comparator | Comparator was children exposed to lower levels of air pollutant that more highly exposed. |
| Outcomes | ASD cases identified from records-based surveillance of children conducted by ADDM network. |

| Bias domain | Authors’ judgment | | Support for judgment |
| --- | --- | --- | --- |
| Source population representation | Low | | Authors do not go into great detail about population, but ADDM methodology should be representative as these methods are robust and well‐described. Unlikely that selection based on outcome would be directly related to exposure, though there could be an association with exposure (e.g. SES). |
| Blinding | | Probably low | Although no mention of blinding, exposures were determined based on census tract and estimated using computer modeling, which should minimize likelihood that there is a risk of bias due to lack of blinding. |
| Exposure assessment | | Probably high^*^ | Judgment supported by 1996 US EPA National Air Toxics Assessment Table A: Composite judgments to determine overall certainty. |
| Outcome assessment | | Probably low | Case definition used ADDM methods, relying on developmental records documenting characteristics and behaviors meeting a standardized definition of autism spectrum disorder based on DSM‐IV‐TR. Although no validation by direct clinical assessment was performed in this study, prior validation of ADDM records‐based diagnoses indicate good agreement with ASD diagnostic instruments. |
| Confounding | | Probably high | Many confounding variables listed not included in the analysis (excluding race and SES). Did not have the available data to include other risk factors including maternal age, gestational age, etc. |
| Incomplete outcome data | | Probably low | No missing outcome data noted, but methods could result in missing some individuals. |
| Selective outcome reporting | | Low | All pre-specified outcomes were reported in the pre-specified way. |
| Conflict of interest | | Low | The study does not have a statement regarding conflicts of interest; however, all funding was from the NIH, and the authors are affiliated with academic universities and the CDC, suggesting that it was not supported by any entities with a financial interest in the outcome. |
| Other sources of bias | | Low | No other potential sources of bias. |

^*^TRI data--arsenic, lead, mercury

**References**

1. Windham GC, Zhang L, Gunier R, Croen LA, Grether JK. Autism spectrum disorders in relation to distribution of hazardous air pollutants in the san francisco bay area. Environ Health Perspect. 2006;114(9):1438-44. PubMed PMID: 16966102; PubMed Central PMCID: PMC1570060.

2. Roberts EM, English PB, Grether JK, Windham GC, Somberg L, Wolff C. Maternal residence near agricultural pesticide applications and autism spectrum disorders among children in the California Central Valley. Environmental Health Perspectives. 2007;115:1482-9.

3. Windham GC, King G, Roberts E, Croen LA, Grether J. Autism and distribution of hazardous air pollutants at birth in California. Epidemiology. 2007;18(Suppl 5):S174.

4. Lewandowski TA, Bartell SM, Yager JW, Levin L. An evaluation of surrogate chemical exposure measures and autism prevalence in Texas. Journal of Toxicology and Environmental Health, Part A. 2009;72(24):1592-603.

5. Salvatori, C, Lepri, M, Marrozzini, L, et al. Epicutaneous allergen-specific immunotherapy as a treatment for grass pollen allergy. Allergy: European Journal of Allergy and Clinical Immunology. 2013;68:644.

6. Kalkbrenner AE, Daniels JL, Chen JC, Poole C, Emch M, Morrissey J. Perinatal exposure to hazardous air pollutants and autism spectrum disorders at age 8. Epidemiology. 2010;21(5):631-41. doi: 10.1097/EDE.0b013e3181e65d76. PubMed PMID: 20562626; PubMed Central PMCID: PMC2989602.

7. Trousdale K. Autism Spectrum Disorder and hazardous air pollutants in the U.S. and Maryland. College Park, Maryland: University of Maryland; 2010.

8. Blanchard KS, Palmer RF, Stein Z. The value of ecologic studies: mercury concentration in ambient air and the risk of autism. Reviews on environmental health. 2011;26(2):111-8.

9. Volk HE, Hertz-Picciotto I, Delwiche L, Lurmann F, McConnell R. Residential proximity to freeways and autism in the CHARGE study. Environ Health Perspect. 2011;119(6):873-7. doi: 10.1289/ehp.1002835. PubMed PMID: 21156395; PubMed Central PMCID: PMC3114825.

10. McCanlies EC, Fekedulegn D, Mnatsakanova A, Burchfiel CM, Sanderson WT, Charles LE, et al. Parental occupational exposures and autism spectrum disorder. Journal of autism and developmental disorders. 2012;42(11):2323-34.

11. Becerra TA, Wilhelm M, Olsen J, Cockburn M, Ritz B. Ambient air pollution and autism in Los Angeles county, California. Environ Health Perspect. 2013;121(3):380-6. doi: 10.1289/ehp.1205827. PubMed PMID: 23249813; PubMed Central PMCID: PMC3621187.

12. Jung CR, Lin YT, Hwang BF. Air Pollution and Newly Diagnostic Autism Spectrum Disorders: A Population-Based Cohort Study in Taiwan. PloS one. 2013;8(9). doi: 10.1371/journal.pone.0075510. PubMed PMID: WOS:000325218700093.

13. Pino-López M, Romero-Ayuso DM. Autism Spectrum Disorders and Parental Occupational Exposures. Revista Española de Salud Pública. 2013;87(1):73-85.

14. Volk HE, Lurmann F, Penfold B, Hertz-Picciotto I, McConnell R. Traffic-related air pollution, particulate matter, and autism. JAMA psychiatry. 2013;70(1):71-7. doi: 10.1001/jamapsychiatry.2013.266. PubMed PMID: 23404082; PubMed Central PMCID: PMC4019010.

15. Windham GC, Sumner A, Li SX, Anderson M, Katz E, Croen LA, et al. Use of birth certificates to examine maternal occupational exposures and autism spectrum disorders in offspring. Autism Research. 2013;6(1):57-63.

16. Gong T, Almqvist C, Bolte S, Lichtenstein P, Anckarsater H, Lind T, et al. Exposure to Air Pollution From Traffic and Neurodevelopmental Disorders in Swedish Twins. Twin Research and Human Genetics. 2014;17(6):553-62. doi: 10.1017/thg.2014.58. PubMed PMID: WOS:000345707600007.

17. Kalkbrenner AE, Windham GC, Serre ML, Akita Y, Wang XX, Hoffman K, et al. Particulate Matter Exposure, Prenatal and Postnatal Windows of Susceptibility, and Autism Spectrum Disorders. Epidemiology. 2015;26(1):30-42. doi: 10.1097/ede.0000000000000173. PubMed PMID: WOS:000345878000018.

18. Roberts AL, Lyall K, Hart JE, Laden F, Just AC, Bobb JF, et al. Perinatal air pollutant exposures and autism spectrum disorder in the children of Nurses' Health Study II participants. Environ Health Perspect. 2013;121(8):978-84. doi: 10.1289/ehp.1206187. PubMed PMID: 23816781; PubMed Central PMCID: PMC3734496.

19. Volk HE, Kerin T, Lurmann F, Hertz-Picciotto I, McConnell R, Campbell DB. Autism Spectrum Disorder: Interaction of Air Pollution with the MET Receptor Tyrosine Kinase Gene. Epidemiology. 2014;2014(25).

20. von Ehrenstein OS, Aralis H, Cockburn M, Ritz B. In Utero Exposure to Toxic Air Pollutants and Risk of Childhood Autism. Epidemiology. 2014;25(6):851-8.

21. Raz R, Roberts AL, Lyall K, Hart JE, Just AC, Laden F, et al. Autism spectrum disorder and particulate matter air pollution before, during, and after pregnancy: a nested case–control analysis within the Nurses’ Health Study II cohort. Environ Health Perspect. 2014;123(264-270).

22. Dickerson AS, Rahbar MH, Han I, Bakian AV, Bilder DA, Harrington RA, et al. Autism spectrum disorder prevalence and proximity to industrial facilities releasing arsenic, lead or mercury. Science of the Total Environment. 2015;536:245-51.

23. Dickerson AS, Rahbar MH, Bakian AV, Bilder DA, Harrington RA, Pettygrove S, et al. Autism Spectrum Disorder prevalence and associations with air concentrations of lead, mercury, and arsenic. Pediatric and Perinatal Epidemiology. 2016;Submitted.
